# Improved Rigidin-Inspired Antiproliferative Agents with Modifications on the 7-Deazahypoxanthine C7/C8 Ring Systems

**DOI:** 10.1021/acs.jmedchem.3c02473

**Published:** 2024-06-12

**Authors:** Aletta E. van der Westhuyzen, Naghmana Ashraf, Daleen Conradie, Leigh Loots, Catherine H. Kaschula, Stephen C. Pelly, Liliya V. Frolova, Taylor Landfair, Charles B. Shuster, Tania Betancourt, Alexander Kornienko, Willem A. L. van Otterlo

**Affiliations:** † Department of Chemistry and Polymer Science, Stellenbosch University, Stellenbosch 7600, South Africa; ‡ Department of Biology, New Mexico State University, Las Cruces ,New Mexico 88003, United States; § Department of Physiological Sciences, Stellenbosch University, Stellenbosch 7600, South Africa; ∥ Department of Chemistry, Emory University, 1515 Dickey Drive ,Atlanta ,Georgia 30322, United States; ⊥ Department of Chemistry and Biochemistry, Purdue University, 2101 East Coliseum Blvd. ,Fort Wayne ,Indiana 46805, United States; # Department of Chemistry and Biochemistry, Texas State University, San Marcos ,Texas 78666, United States

## Abstract

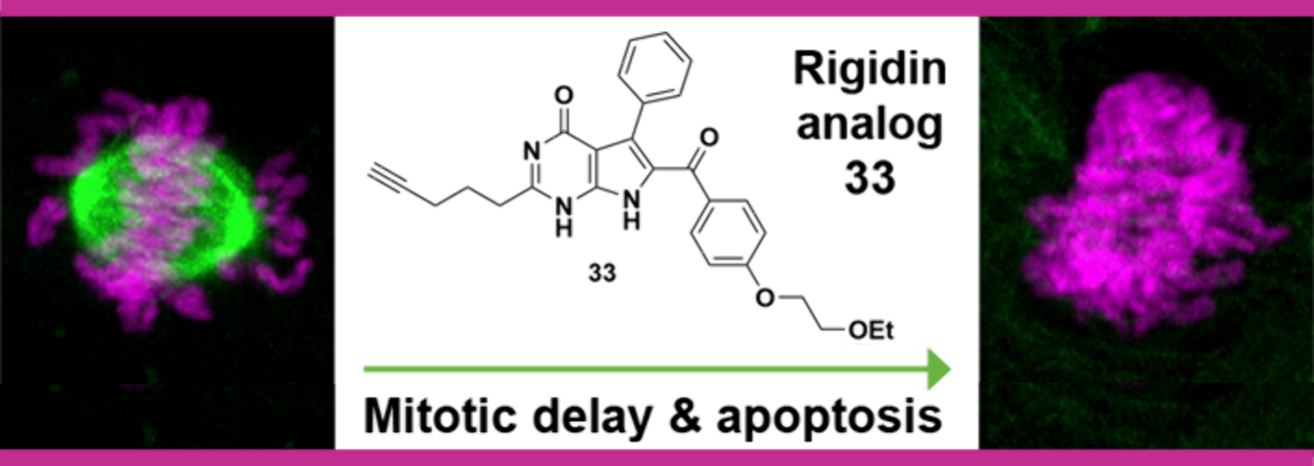

To improve their aqueous solubility characteristics, water-solubilizing groups were added to some antiproliferative, rigidin-inspired 7-deazahypoxanthine frameworks after molecular modeling seemed to indicate that structural modifications on the C7 and/or C8 phenyl groups would be beneficial. To this end, two sets of 7-deazahypoxanthines were synthesized by way of a multicomponent reaction approach. It was subsequently determined that their antiproliferative activity against HeLa cells was retained for those derivatives with a glycol ether at the 4′-position of the C8 aryl ring system, while also significantly improving their solubility behavior. The best of these compounds were the equipotent 6-[4-(2-ethoxyethoxy)benzoyl]-2-(pent-4-yn-1-yl)-5-phenyl-1,7-dihydro-4*H*-pyrrolo[2,3-*d*]pyrimidin-4-one **33** and 6-[4-(2-ethoxyethoxy)benzoyl]-5-(3-fluorophenyl)-2-(pent-4-yn-1-yl)-1,7-dihydro-4*H*-pyrrolo[2,3-*d*]pyrimidin-4-one **59**. Similarly to the parent **1**, the new derivatives were also potent inhibitors of tubulin assembly. In treated HeLa cells, live cell confocal microscopy demonstrated their impact on microtubulin dynamics and spindle morphology, which is the upstream trigger of mitotic delay and cell death.

## Introduction

**Figure 1:**
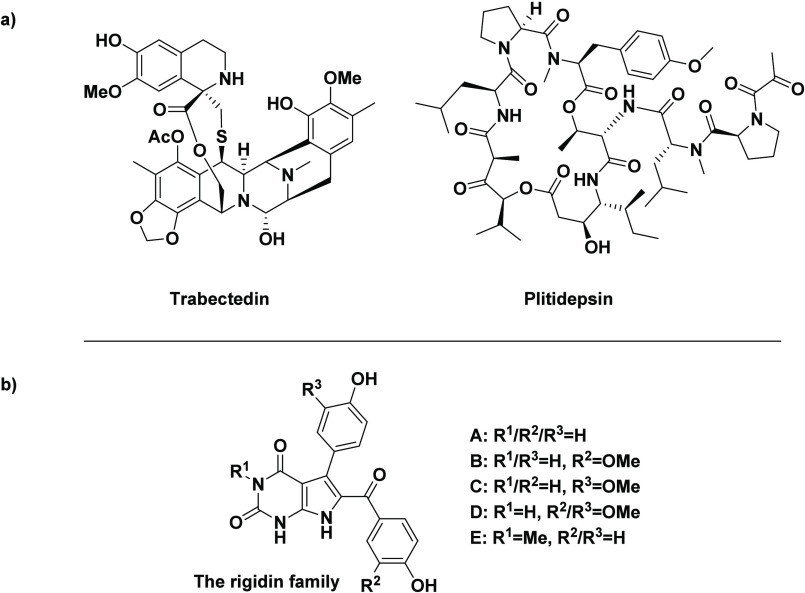
Structures of (a) trabectedin (Yondelis) and plitidepsin (dehydrodidemnin B, Aplidin); (b) the naturally occurring rigidins.

Despite significant advances in the field of oncology, cancer remains a leading cause of death.^1−3^ In response to the rapid emergence of chemoresistance, researchers are continually being challenged to develop more effective chemotherapies.^4^ Throughout history, natural products have been recognized as an invaluable contributor to drug discovery.^5^ Notably, these evolutionary refined molecules still play a dominant role in modern drug discovery, often serving as structural muses for the design of novel therapeutics.^6,7^ The marine environment offers a rich source of unexplored chemical biodiversity and potential.^8,9^ In this regard, marine-derived alkaloids, isolated from tunicates, have led to the discovery of interesting bioactive molecules.^10,11^ Two such examples include trabectedin (Yondelis) and plitidepsin (dehydrodidemnin B, Aplidin), which have received approval from the US Food and Drug Administration (FDA) as cancer treatments (Figure 1a).^12^ Another intriguing class of natural products isolated from marine tunicates are the pyrrolopyrimidine alkaloids, the rigidins, the structures of which are shown in Figure 1b.^13−15^

**Figure 2:**
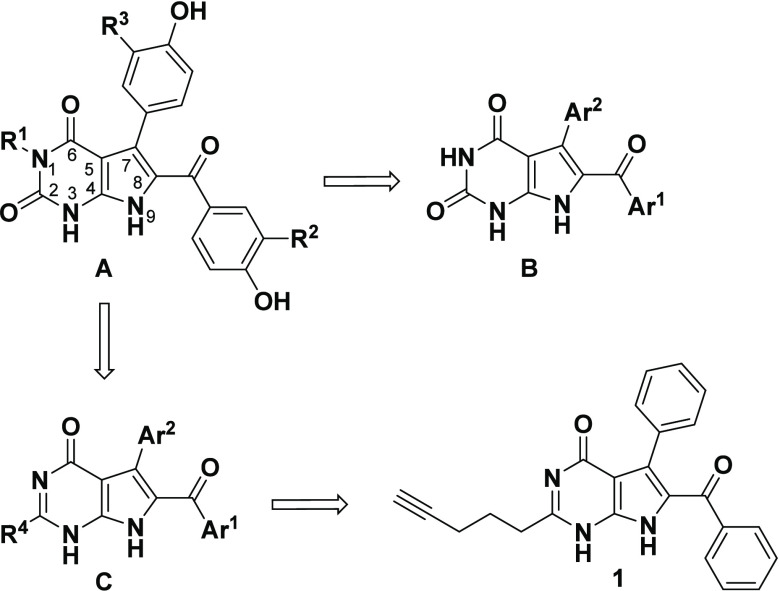
General structure of the rigidin alkaloids **A** (R^1^ = H, Me; R^2^ = H, OMe; R^3^ = H, OMe) and the rigidin mimetics: 7-deazaxanthines **B**, 7-deazahypoxanthines **C** (R^4^ = linear alkyl groups) and the potent C2-substituted analogue **1**.

Over the past decade, the rigidins have served as an inspiration toward the synthesis of a novel range of 7-deazahypoxanthine anticancer candidates.^16−21^ The isolation, refinement of total syntheses, biochemical evaluation, and development toward potent rigidin-inspired small molecules have been reviewed.^22^ Although the natural alkaloids **A** and their first-generation analogues, 7-deazaxanthines **B**, exhibited no significant antiproliferative activities, the rigidin-mimetic 7-deazahypoxanthines **C** display impressive anticancer activity against a range of cancer cell lines (Figure 2).^17^

According to initial mechanistic studies, the antiproliferative activity of the 7-deazahypoxanthines has been attributed to the suppression of microtubule dynamics during cell division by binding to the colchicine site of β-tubulin.^17^ In a recent study, examination of spindle morphology and chromosome alignment in rigidin-treated HeLa cells revealed the cells inability to establish proper bioriented chromosome attachment at the metaphase plate.^23^ This disruption was proposed to lead to the observed mitotic arrest and cell death.^23^ Another impressive feature of these rigidin analogues was their ability to maintain their potency against cells representing cancers with poor prognoses, as well as metastatic and multidrug resistant cancers. Remarkably, it has been shown that the multidrug resistant (MDR) proteins, ABCG2/ABCC1/ABCB1, do not confer resistance to the rigidin analogues, and it is postulated that these compounds are able to cross the MDR transporter-guarded epithelial barriers.^23^ Additionally, the modulatory effects of the 7-deazahypoxanthines on selected MDR proteins revealed no long-term effects on the expression or function of the MDR proteins studied.^23^ Collectively, these findings provide further impetus to expand on the further development of rigidin-inspired therapeutic agents.

Besides the promising anticancer activity exhibited by the rigidin-mimetic 7-deazahypoxanthines **C**, our interest toward these pyrrolo[2,3-*d*]pyrimidine scaffolds was triggered by the attractive synthetic strategy to pyrroles using a versatile multicomponent reaction (MCR).^16,24,25^ This innovative approach has enabled the rapid derivatization of this scaffold culminating in the discovery of lead compound **1** of which the optimum alkynyl-chain chain length was determined through a detailed structure–activity relationship (SAR) study (Figure 1).^19^ Biological evaluation of **1** against several human colon cancer lines demonstrated exceptional potency with GI_50_ values approaching single-digit nanomolar values, with normal cells being less sensitive.^19^ Moreover, while the natural rigidins showed only weak antiproliferative activity against human cancer cells (>100 μM against HeLa and MCF-7 cells),^17^ the rigidin-mimetic **1** is highly active with a GI_50_ of 0.022 and 0.035 μM, respectively, against the same cell lines.^19^ In addition, evaluation of **1** in a mouse model for colon cancer revealed significant tumor size reduction at a low dose (3 mg/kg), while maintaining body weight and with no observable side effects.^19^

**Figure 3:**
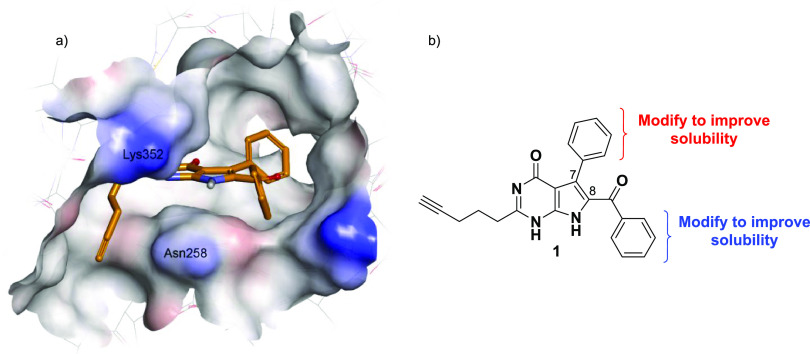
(a) Molecular modeling of rigidin-mimetic **1** within the colchicine site of β-tubulin.^19^ (b) Envisaged areas to make small structural changes to potentially improve solubility.

To date, **1** serves as a lead compound among the rigidin-mimetic series. Its design was based on the introduction of linear alkyl-substituents at C2 to circumvent possible photooxidation toward the C2–H-containing molecules.^19^ Molecular modeling simulations of the binding of **1** within the colchicine site of β-tubulin revealed a narrow hydrophobic channel in the region of the Asn258 and Lys352 amino acid chains, which appeared to accommodate the linear alkyl substituents at C2 (Figure 3a).^19^ Although the 7-deazahypoxanthines represent tremendous potential as therapeutic agents, these compounds suffer from poor aqueous solubility. The pyrrolo[2,3-*d*]pyrimidine scaffold and aryl groups constituting the core framework of the rigidin analogues contribute to its hydrophobicity, planarity, and strong π–π interactions. In addition to these tight crystal packing characteristics, a number of hydrogen bonding partners allow for a strong network of intermolecular hydrogen bonding in the solid state, requiring additional energy to solvate the molecules.^26^

The poor water solubility of the rigidin-mimetics poses a potential problem,^27−29^ especially in oncology where high dose requirements are often utilized.^30^ Solubility limitations are known to contribute to poor bioavailability and acute toxicity, which may lead to ultimate drug development failure.^31^ Although the strategy to improve aqueous solubility appears straightforward, the physical properties and structural elements which influence solubility also simultaneously control drug potency.^32,33^ Consequently, the dilemma is in finding the balance between solubility and potency.

While multiple tactics have been developed to improve aqueous solubility of poorly soluble compounds, such as formulation techniques and polymeric drug delivery carriers,^34,35^ modifying the chemical structure of the active molecule during early discovery stages can avoid potential risks and additional costs during clinical trials and manufacturing.^36^ Considering the proposed hydrophobic binding pocket of the 7-deazahypoxanthines and the solubility impediment associated with these rigidin-like structures, the primary endeavor of this research was directed toward improving their solubility via small structural modifications, while maintaining potency. To achieve this outcome, the research presented the following aims:

a) The synthesis of novel 7-deazahypoxanthines with structural modifications on the C7 or C8 aryl rings (see Figure 3b).
b) The evaluation of the new compounds’ antiproliferative effects against HeLa cervical cancer cells, and if still active,
c) The need to confirm the microtubule disruption mechanism of action of the modified compounds, and the use of live cell imaging to understand their impact on cancer cells.

## Results and Discussion

### (a-i) Incorporation of Water-Solubilizing Groups into a 7-Deazahypoxanthine Lead via the MCR-Based Strategy

The introduction of solubilizing appendages onto a core scaffold or making modifications, which can distort the extent of planarity (and π–π stacking) within a molecular framework, have proved successful strategies to enhance aqueous solubility.^26,37^ As mentioned, the main challenge regarding this approach is to incorporate solubilizing groups without sacrificing potency.

**Scheme 1:**
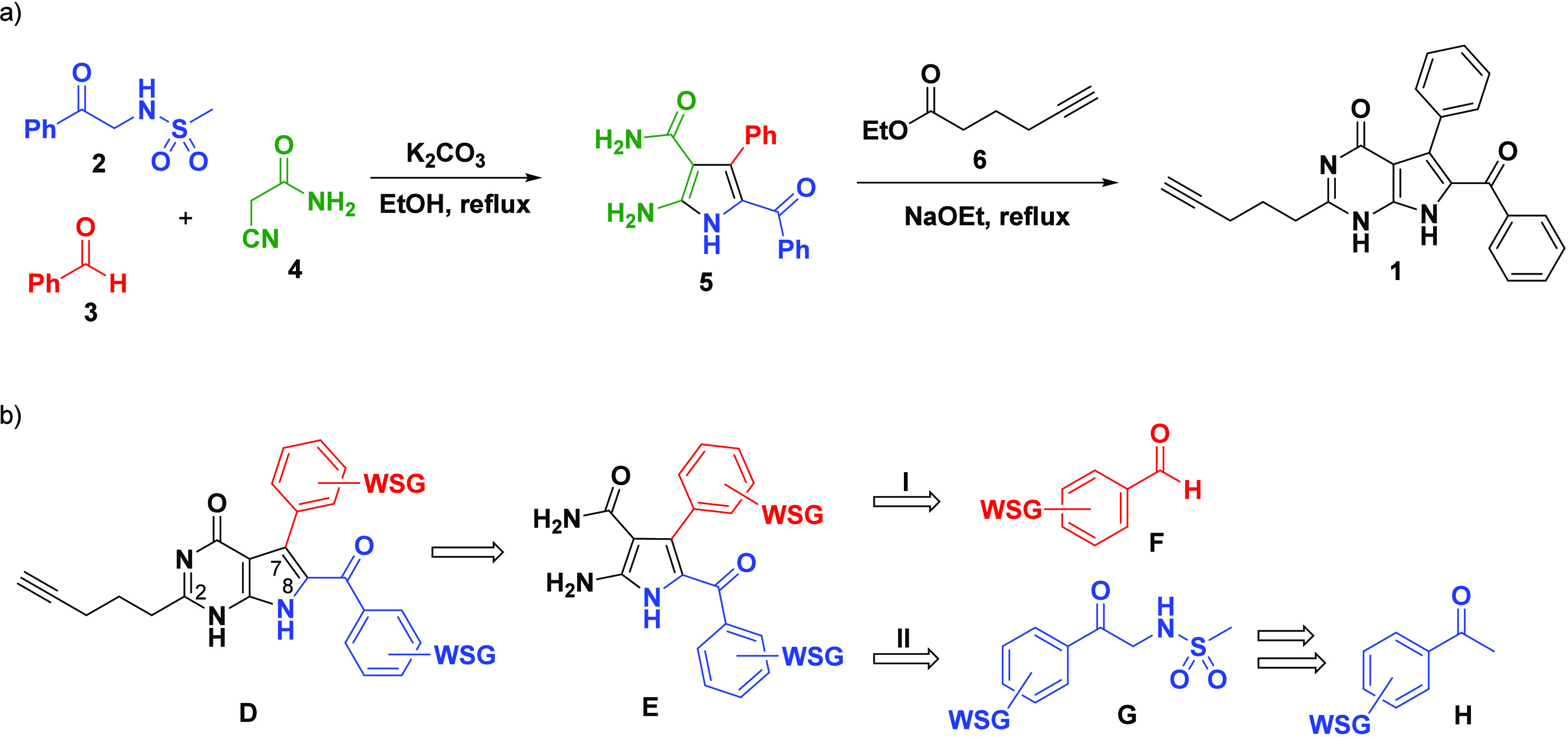
(a) Established Synthetic Strategy to Compound **1**(18) and (b) Proposed Retrosynthetic Strategy to Incorporate Water-Solubilizing Groups (WSGs) onto Target **D** via the Aldehyde Component **F** (Red) or Sulfonamido Acetophenone Component **G** (Blue) (Synthesised from Acetophenone **H** Starting Material), Prior to the MCR Affording Aminopyrrole **E**

Motivated by the excellent in vivo activity of lead compound **1**, the desire to emulate its scaffold and introduce solubilizing elements, which could be tolerated in the binding domain of β-tubulin, was reinforced. Also, the low toxicity indicates that these molecules are highly specific for potent tubulin binding. The synthesis of **1** is based on the MCR strategy, as developed by Magedov and co-workers,^16,24^ to afford the respective aminopyrrole precursor **5**. The latter reaction was achieved by treating sulfonamido acetophenone **2**, benzaldehyde **3**, and cyanoacetamide **4** with granular K_2_CO_3_ in ethanol at reflux (Scheme 1a). From the MCR-synthesized pyrrole **5**, the 7-deazahypoxanthine framework of **1** was then constructed via a ring-closing reaction with ethyl-hex-5-ynoate **6** in the presence of sodium ethoxide at elevated temperatures (Scheme 1a).^18^ Encouraged to maintain the optimized linear terminal alkyne chain at C2 of **1**, our synthetic strategy was directed toward the incorporation of water-solubilizing groups (WSGs) onto the aryl rings positioned at C7 and C8 of the 7-deazahypoxanthine structure **D**. As depicted in Scheme 1b, to avoid reduction of the alkyne functionality or unwanted side reactions, the strategy to incorporate the WSGs involved installation prior to the MCR. Thus, the synthetic routes relied on the incorporation of the WSGs either via the aldehyde component **F** (red) or the sulfonamido acetophenone component **G** (blue), affording the respective pyrrole **E** (Scheme 1b).

In general, route (I) was found to be relatively undemanding, as the aldehyde components **F** could mostly be obtained commercially. On the other hand, the introduction of the WSG on the sulfonamide-derived ring was more taxing. This route (II) required a lengthier synthesis to obtain the necessary sulfonamido acetophenone component **G**, starting from the respective acetophenone **H**. It was our opinion that the interchange of the WSGs on the aryl rings could provide insightful information regarding the accommodation of these groups within the target domain of β-tubulin.

**Scheme 2:**
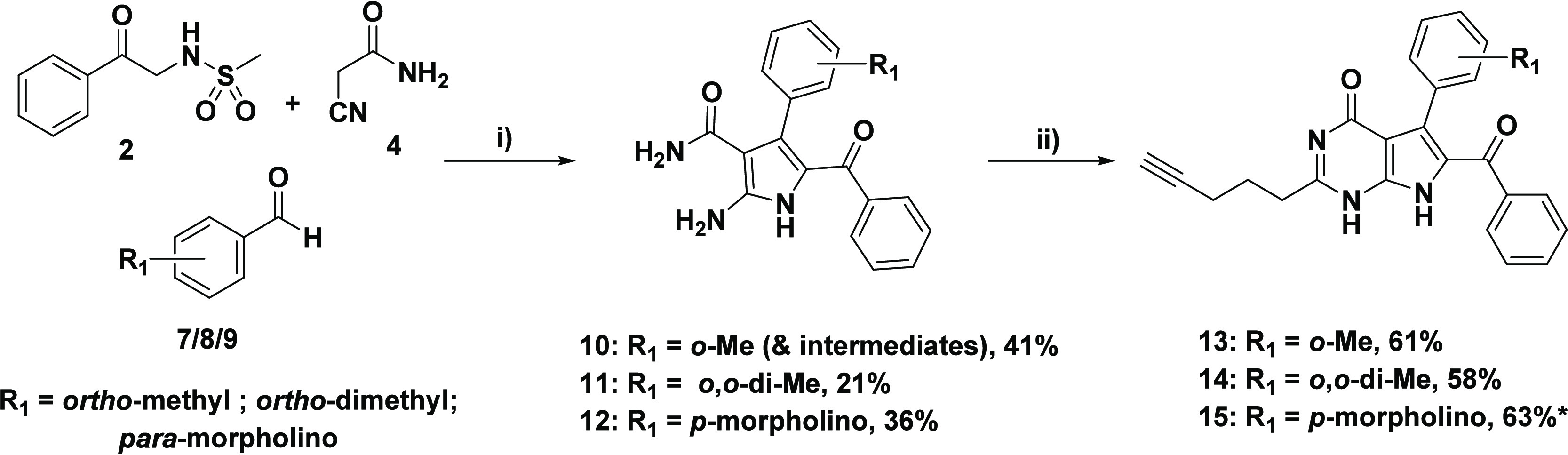
Incorporation of WSGs from the Aldehyde Component Reagents and conditions: (i) sulfonamido acetophenone **2** (1 equiv), cyanoacetamide **4** (1.3 equiv), benzaldehyde derivatives **7**/**8**/**9** (1.3 equiv), K_2_CO_3_ (0.6 equiv), EtOH, reflux, 20–24 h; (ii) ethyl hex-5-ynoate **6** (8–10 equiv), 0.65 mM NaOEt (20–25 equiv of Na), (DMSO added for **15**), 80 °C, 24–48 h (*note: some impurity in sample of compound **15**, see later).

**Scheme 3:**
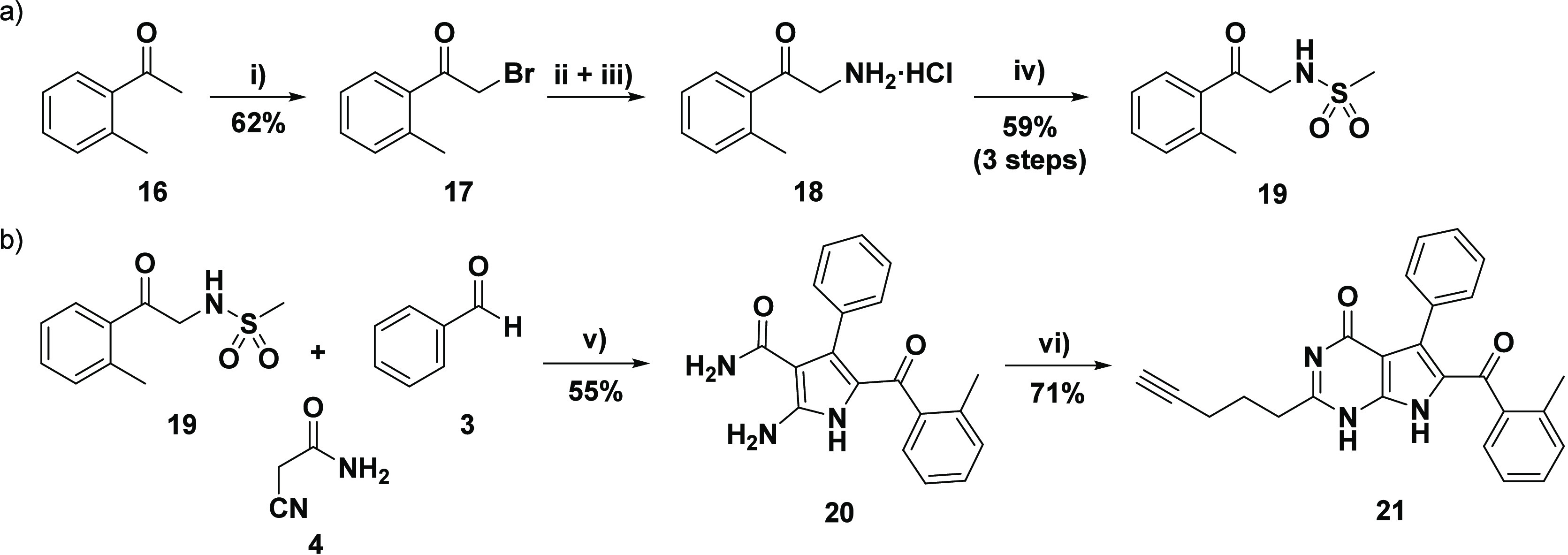
Synthesis of Target Compound **21** Reagents and conditions: (a) (i) PTAB (1.1 equiv), THF, RT, 1 h; (ii) hexamethylenetetramine (1.1 equiv), C_6_H_5_Cl, 30 °C, 12 h; (iii) EtOH/HCl (conc.), RT, 36 h; (iv) MsCl (1.5 equiv), Et_3_N (2.5 equiv), acetone/H_2_O (5:1), 0 °C–RT, 2 h; (b) (v) cyanoacetamide **4** (1.3 equiv), PhCHO **3** (1.3 equiv), K_2_CO_3_ (0.6 equiv), EtOH, reflux, 18 h; vi) ethyl hex-5-ynoate **6** (8 equiv), 1.30 mM NaOEt (20 equiv of Na), 80 °C, 48 h.

**Scheme 4:**
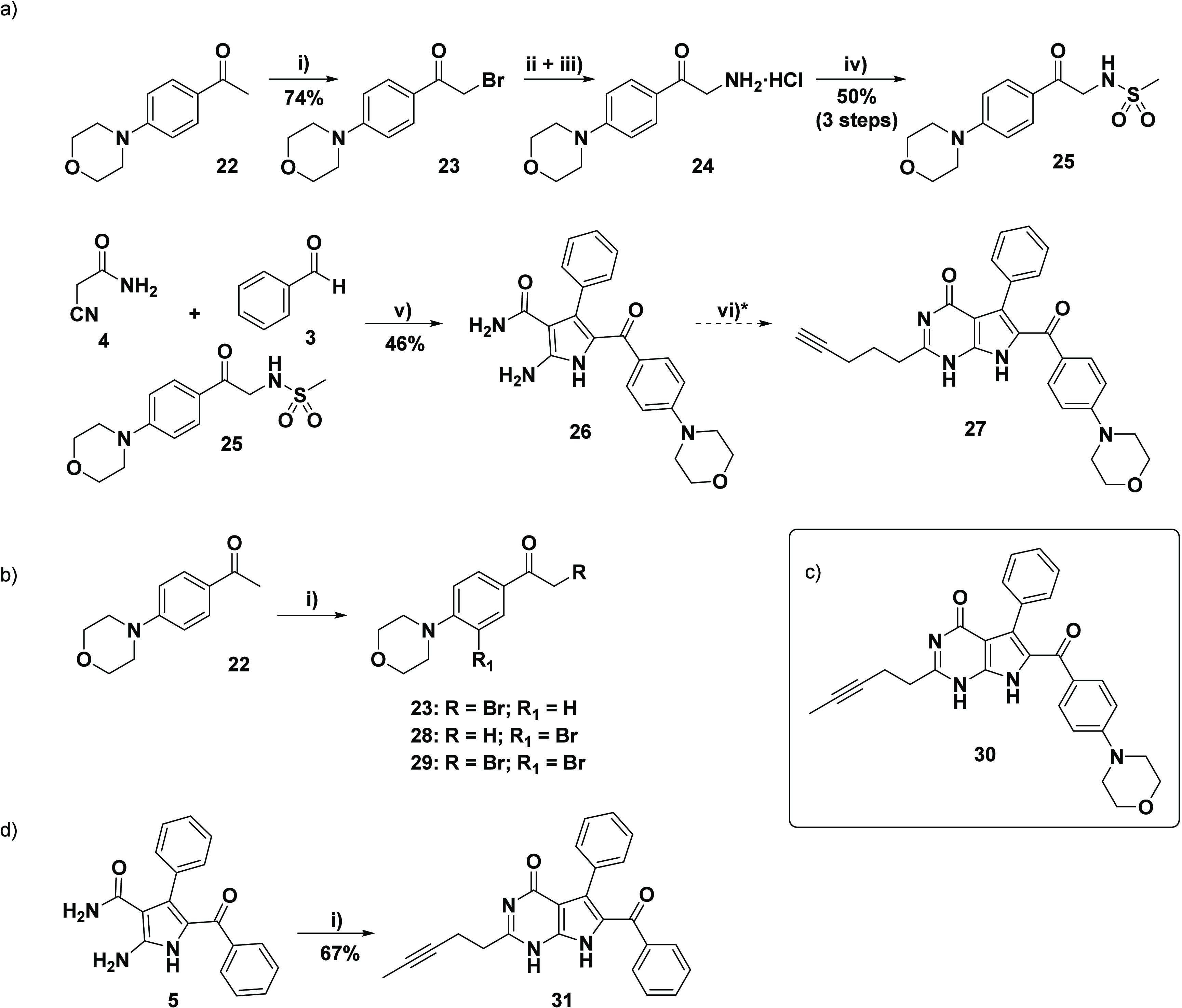
(a) Synthetic Overview toward Target Compound **27** Reagents and conditions: (a) (i) PTAB (1.1 equiv), THF, 30 °C, 1 h; (ii) hexamethylenetetramine (1.1 equiv), C_6_H_5_Cl, 30 °C, 5 h; (iii) EtOH/HCl (conc.), RT, 36 h; (iv) MsCl (2.5 equiv), Et_3_N (5.5 equiv), acetone/H_2_O, 0 °C–RT, 5 h; (v) cyanoacetamide **4** (1.3 equiv), PhCHO **3** (1.3 equiv), K_2_CO_3_ (0.5 equiv), EtOH, reflux, 18 h; (vi) ethyl hex-5-ynoate **6** (8 equiv), 1.30 mM NaOEt (20 equiv of Na), DMSO, 100 °C, 12 h (*reaction conditions vi) afforded compound **30** (see box c for structure) bearing the isomerized alkyne, while **27** was obtained at a lowered temperature of 55 °C); (b) (i) various conditions, resulted in α-monobromination (**23**) and substituted side-products (**28**/**29**) due to electrophilic aromatic side-reactions. Refer to experimental information for specific reaction conditions. (d) Synthesis of internal alkyne of lead compound (**31**); Reagents and conditions: (i) ethyl hex-5-ynoate **6** (8 equiv), NaOEt (21% in EtOH, 15 equiv), DMSO, 90 °C, 12 h.

The selected reagents required for the first set of targets included an *ortho*-substituted methyl group (**7**/**16**), as well as a *para*-morpholine group (**9**/**22**) (respective structures depicted in Schemes 2**–**4). The first set of target compounds were selected based on the commercial availability of their starting reagents. Furthermore, the utilization of these reagents did not demand any protecting group strategy, nor did we foresee the risk of encountering unwanted side reactions during the synthetic procedures. While morpholine is a well-known solubilizing element in medicinal chemistry,^38,39^ the introduction of the hydrophobic methyl group onto the *ortho*-position of aryl rings can lead to an increase in dihedral angle, effectively reducing π–π stacking and improving aqueous solubility.^26,36^ The set of aldehyde components utilized in this synthetic study also included the readily available *ortho*-dimethyl derivative **8**.

Unfortunately, the MCR of the respective aldehyde components (**7**–**9**) resulted in overall low yields of the 2-aminopyrroles **10**-**12**, with multiple side-products being observed by TLC (Scheme 2). Purification by silica gel column chromatography proved to be challenging, yet a few intermediate species, including the mesyl-protected pyrroles and α,β-unsaturated nitrile species (derived from the initial Knoevenagel condensation), were identified by ^1^H NMR spectroscopy and HRMS (see the Supporting Information for structures). As a result of purification difficulties via column chromatography, the yield obtained for pyrrole **10** included certain inseparable intermediates (∼10%). The low yields obtained from the *ortho*-methyl and dimethyl *ortho*-substituted aldehydes **7**/**8** could be ascribed to the steric clash induced by the *ortho*-methyl groups, which negatively affects the usually fast Michael-type addition of the α-carbon sulfonamido acetophenone (see ref (24)). An alternative reason could be due to the modest electron-donating effect of the methyl group(s), which increases electron density in the ring through the inductive effect, thereby making the conjugated unsaturated Michael acceptor less electrophilic. The low yield of the pure *para*-morpholine pyrrole **12** was also affected by purification difficulties. The final step involved the synthesis of the target 7-deazahypoxanthines **13**–**15** (Scheme 2). The ring-closing reactions were carried out in a similar manner to that of lead compound **1**, using a more concentrated solution of NaOEt prepared in situ to promote product formation. Another small, yet essential change in the reaction toward **15** was the addition of a small volume of DMSO to aid the solution of pyrrole **12** in the reaction mixture upon heating to 80 °C. After reaction completion, neutralization, and purification, the target compounds (**13**–**15**) were fully characterized. All compounds were fully characterized by way of their ^1^H and ^13^C NMR spectra, in addition to accurate mass analysis (HRMS)—this statement holds for all new compounds generated in this research. Hence, formation of the expected pyrrolo[2,3-*d*]pyrimidine cores were confirmed, together with their distinctive alkyl chains and morpholine/methyl group(s). However, for compound **15**, which required DMSO for solubilization, an unexpected singlet at 1.72 ppm and a shoulder to the broad -N*H* singlet at 12.50 ppm raised some concern in terms of possible alkyne isomerization. This aspect is discussed later.

Next, the synthesis of the 7-deazahypoxanthine target compounds, via introduction of an *ortho*-methyl and *para*-morpholine from the sulfonamido acetophenone-derived component (route II), was embarked on. Initial focus was directed toward the generation of the desired sulfonamido acetophenone components **G** from the respective acetophenone starting reagent **H** (Scheme 1b). The strategy involved α-bromination utilizing phenyltrimethylammonium tribromide (PTAB), followed by a Delépine reaction to generate the desired 2-aminoacetophenone hydrochloride derivative.^40^ The more stable amine salt was pursued to avoid possible intermolecular self-condensation of the phenacyl amine species (α-aminoketone). Finally, mesylation afforded the desired component **G** for subsequent MCR and ring closure (Scheme 1b).

Using this general approach, the synthesis of the target 7-deazahypoxanthine **21** was carried out without complications (Scheme 3a). α-Bromination of *ortho*-methyl acetophenone **16** afforded the brominated species **17** in a moderate yield. The Delépine reaction was then conducted by treating **17** with hexamethylenetetramine, followed by an acid-catalyzed ethanolysis (EtOH/HCl) to unveil the amine hydrochloride product **18**. Although some of the product **18** was found to precipitate, the majority of this material remained dissolved in the EtOH/HCl solution. Following solvent removal, the product **18** (and NH_4_Cl byproduct) was obtained and used without further purification. The mesylation reaction was carried out in a mixture of acetone/water using methanesulfonyl chloride and a slight excess of Et_3_N. Finally, the desired sulfonamido acetophenone component **19** was obtained from **17** in a satisfactory yield of 59% over three reaction steps. Having all three components in hand, the MCR was carried out utilizing sulfonamido acetophenone derivative **19**, cyanoacetamide **4**, and benzaldehyde **3** (Scheme 3b). The general MCR method was employed using granular anhydrous K_2_CO_3_ in refluxing ethanol to afford polysubstituted pyrrole **20**. The ^1^H and ^13^C NMR spectra of **20** proved to be very similar to that of unsubstituted pyrrole **5** with an additional methyl singlet located at 2.09 and 18.8 ppm in the respective ^1^H and ^13^C NMR spectra. Finally, the ring-closing reaction of **20** with the alkyne ester **6** was undertaken, generating 7-deazahypoxanthine **21** in a relatively high yield (Scheme 3b), the NMR spectra of which provided evidence of all the expected structural features.

The synthesis of target compound **27**, bearing a *para*-morpholine at C8 of the 7-deazahypoxanthine, involved a similar strategy to that described for **21** (Scheme 3). However, unlike the ease with which the previous synthetic endeavor (Scheme 3) was accomplished, several hurdles were encountered. Initial attempts at the bromination reaction suffered from poor regioselectivity, which was ascribed to the electron-donating ability of the morpholine group promoting aryl-bromination. Thus, in addition to the desired α-monobromination product **23**, the aryl-brominated side-product **28** and the overbrominated species **29** were identified (Scheme 4b). Characterization of the brominated species was done by inspection of the respective ^1^H NMR spectra with specific focus on the difference in chemical shift and integration of the methyl and methylene group, as well as the integration/loss in symmetry of the aromatic protons. Optimization of the α-monobromination reaction, employing PTAB at moderate temperature for a relatively short reaction time, eventually led to the formation of **23** in an acceptable yield (see Scheme 4a).

Application of the Délepine reaction on compound **23**, following the same procedure as described before, gave the amine HCl species **24**, which was used without further purification. Likewise, the mesylation reaction was carried out as before, albeit that more methanesulfonyl chloride and Et_3_N were required for complete consumption of the starting material. Overall, the sulfonamido acetophenone component **25** was obtained in an acceptable yield of 50% over the three reaction steps. The MCR was subsequently performed using the general procedure, yielding pyrrole product **26** in moderate yield (see Scheme 4a).

Finally, the pyrrole ring-closing reaction with ethyl hex-5-ynoate **6** could be embarked upon. As with its morpholine counterpart **12** (derived from the benzaldehyde component), pyrrole **26** lacked solubility in the NaOEt reaction solution. Initial use of diglyme^41^ as an alternative solvent (at 100 °C) was not successful, as solid pyrrole **26** remained undissolved in our hands and DMSO proved to be the only additive, which allowed for complete dissolution of **26**. Therefore, the ring-closing reaction was carried out in NaOEt prepared in situ, with the addition of the minimal volume of DMSO to dissolve **26** at 100 °C. Compared to the reaction with pyrrole **12**, the ratio of DMSO to NaOEt required for dissolution was much higher. To our surprise, the reaction proceeded exceedingly well, with full conversion to a ring-closed product within 12 h of stirring at 100 °C in a sealed microwave vial. Upon neutralization, the precipitated solid was collected and purified via column chromatography to give a product, which was obtained in high yield (83%) and excellent purity (99%).

Disconcertedly, close inspection of the NMR spectra of product (**27**) revealed the presence of unexpected signals, not entirely supporting the structure. Although the HRMS obtained (467.2078 amu obtained, expected 467.2083 amu) supported the required molecular formula, examination of the NMR spectra indicated that what we had in hand was a compound in which the alkyne had isomerized into the thermodynamically favored internal position (see Scheme 4c for structure of actual compound **30**).^42^ Examination of compound **30**’s ^1^H NMR spectrum showed all the pyrrolo[2,3-*d*]pyrimidine core signals, together with the morpholine methylene signals. However, a distinct change in the alkyne alkyl signals was evident. While two of the three methylene signals were present at 2.79 ppm and 2.62–2.56 ppm, one methylene signal was missing, together with the alkyne proton signal. At the same time, a new signal was identified upfield at 1.72 ppm. This peak appeared as a triplet and integrated for a total of three protons. With this information, we deduced that isomerization of the terminal alkyne to the internal alkyne had occurred, which supported the presence of the two remaining methylene signals and the additional methyl signal. Looking at the ^13^C NMR spectrum of **30**, this deduction was further verified by a shift in the alkyne carbon signals located at 78.0 and 76.6 ppm, together with an upfield shift to 3.2 ppm ascribed to the new methyl carbon. To validate the isomerization of the terminal alkyne, the reaction was repeated using the unsubstituted pyrrole **5** as starting material. In this case, commercially available NaOEt (21% in EtOH) was utilized together with the DMSO additive and the reaction was stirred at a slightly lower temperature (90 °C) (Scheme 4d). The NMR spectra of the purified product exhibited the anticipated internal alkyne alkyl signals, thereby confirming the structure of compound **31**. (For the distinctive terminal and internal alkyne alkyl chain proton signals of 7-deazahypoxanthine **1** vs **31**, as displayed in the expanded region of their respective ^1^H NMR spectra, see Figure S1).

**Figure 4:**
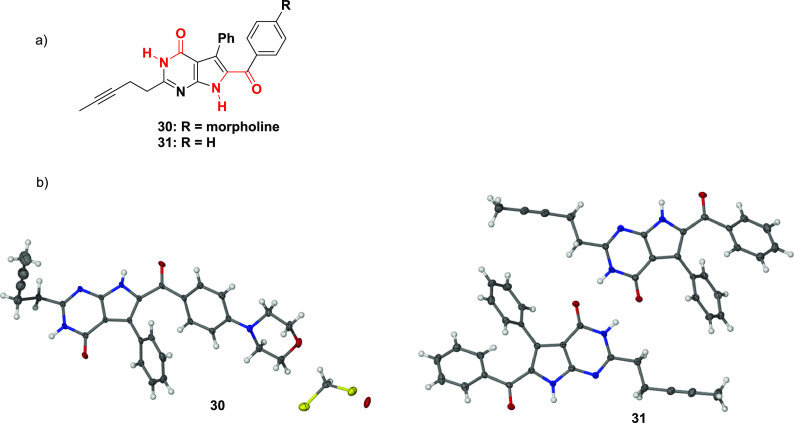
(a) Structural representation of 7-deazahypoxanthine tautomers with H-bonding acceptor/donor pairs highlighted in red. (b) Asymmetric units of the crystal lattices of 7-deazahypoxanthines **30** (CCDC submission: 2178967) and **31** (CCDC submission: 2178968), clearly showing the linear internal alkyne.

The presence of the internal alkyne in compounds **30** and **31** was further confirmed by their respective crystallographic structures, as determined through single-crystal X-ray diffraction studies (Figure 4b). Visual inspection of both crystal structures highlighted the increased rigidity exhibited by the internal alkyne. Corresponding to the crystal structure reported by Dasari et al.,^23^ the pyrrolo[2,3-*d*]pyrimidine tautomers shown were the favored constitutional isomers upon crystallization (Figure 4a). Notably, two distinct H-donor/acceptor pairs are present within the crystal structure, which could facilitate intra- and intermolecular H-bonding, providing possible reasons for the low solubility of the solid compounds. The crystal structure of **30** included cocrystallized solvent molecules of DCM and water, as seen in the asymmetric unit. In addition, the asymmetric unit of **31** comprised of two molecules, as the phenyl rings of the independent molecules were in slightly different orientations.

During the initial procedure to synthesize compound **27**, two changes were implemented to enable solubilization of the pyrrole starting material **26**, the inclusion of DMSO as a solvent and a slight increase in temperature (from 80 to 100 °C). These two alterations effectively resulted in the internalization of the alkyne, a process which was further verified during the synthesis of analogue **31**. In retrospect, the distinctive proton signals supporting alkyne internalization suggests that the C7 *para*-morpholine sample **15** also contains a fraction (approximately 25% as determined from ^1^H spectra integration) of its internal alkyne isomer.^42^ Notably, solubilization of pyrrole **12** required only a small volume of DMSO without further temperature increase, thereby resulting in alkyne internalization to a much lesser extent. The migration toward the internal alkyne was not completely unexpected, since it is the more stable and thermodynamically favored species.^43^ This type of isomerization is generally achieved through use of moderately strong bases at high temperatures (>100 °C). A literature search revealed that this reaction has typically been achieved using potassium *tert*-butoxide in DMSO at low/moderate temperatures, or by the use of KOH in water or ethanol at high temperatures (for a noncomprehensive set of examples, see refs (43−52)). Reaction conditions involving the use of NaOEt in DMSO at elevated temperatures (90 °C–100 °C) for this type of isomerization were not found during the literature search. As shown for NaOMe, the base strength of NaOEt is significantly improved in DMSO as it is a good solvator of the sodium cation, thereby improving the effectiveness of the alkoxide base.^53^ We therefore concluded that the alkyne isomerization detected during the ring-closing reaction occurred as a result of a combination of the DMSO as solvent and temperature increase.

As efforts to form the original, unisomerized *para*-morpholine target compound **27** using a “contra-thermodynamic zipper reaction” were unsuccessful, an alternative strategy toward the desired terminal alkyne analogue **27** was performed. The “contra-thermodynamic” alkyne migration of the stable internal to the least stable terminal alkyne was attempted utilizing the lithium salt of 1,3-diaminopropane in the presence of potassium *tert*-butoxide.^54^ Unfortunately, multiple polar byproducts formed. Moreover, since the physical properties and R_f_ of the internal and terminal isomers were almost identical, tracking the progress of the reaction using TLC proved to be problematic. The second approach involved conducting the general ring-closing reaction of pyrrole **25** with ethyl hex-5-ynoate **6** at a much lower temperature (55 °C); however, the addition of DMSO remained crucial to ensure the solubility of **25**. Although the efficiency of the ring-closing reaction was hampered at the lower temperatures, leading to unreacted starting material **25**, enough target compound **27** was obtained for characterization and biological evaluation. The ^1^H and ^13^C NMR spectra of **27** was scrutinized, revealing indicative signals, which confirmed the expected chemical structure. Interest was particularly directed to the upfield region of the spectra, as the alkyne alkyl chain signals could provide support for the formation of the terminal alkyne. From the ^1^H NMR spectrum, the terminal proton was clearly visible as a triplet at 2.83 ppm, along with the three expected methylene signals at 2.71 ppm (triplet), 2.27 ppm (triplet of doublets), and 1.97–1.85 ppm (apparent pentet). Moreover, the absence of the internal methyl signal at 1.72 ppm further validated the formation of the terminal alkyne. Although only 20 carbon signals were observed in the ^13^C NMR spectrum of **27** (two missing quaternary carbon signals), this was ascribed to the slow relaxation time experienced by the quaternary carbons. Owing to the difficulty of separating the terminal isomer **27** from its minor internal product **30**, we were satisfied to obtain the product **27** in 91% purity, as determined by LC-MS.

Despite the few synthetic obstacles encountered, the first set of target 7-deazahypoxanthines (**13**–**15**, **21**, **27**, **30**, **31**) were successfully obtained in high purity as required for further biological evaluation.

### (b-i) Antiproliferative Activities of Initial Set of Compounds

To assess the effect that the structural changes have on antiproliferative activity, the synthesized 7-deazahypoxanthines (**13**–**15**, **21**, **27**, **30**, **31**) were evaluated against HeLa cells.^55^ The concentrations at which the analogues inhibit cancer cell growth by 50% (GI_50_) are presented in Table 1.^55^ The most prominent trend was observed between the analogues possessing substituents derived from the benzaldehyde component (**13**–**15**) and those derived from the sulfonamido acetophenone component (**21**, **27**, and **30**). Overall, an impairment in antiproliferative activity was recognized following the introduction of substituents on the C7-aryl ring. Among this group (**13**–**15**), the introduction of the *para*-morpholino WSG of analogue **15** led to a dramatic 500-fold decrease in antiproliferative activity, leading to a double-digit micromolar activity. The latter finding was not completely surprising, as it corroborated with the observation made by Frolova et al., whereby the introduction of hydrophilic pyridine and hydroxyl groups at C7 led to an unfavorable loss in solvation energy.^17^

**Table 1:**
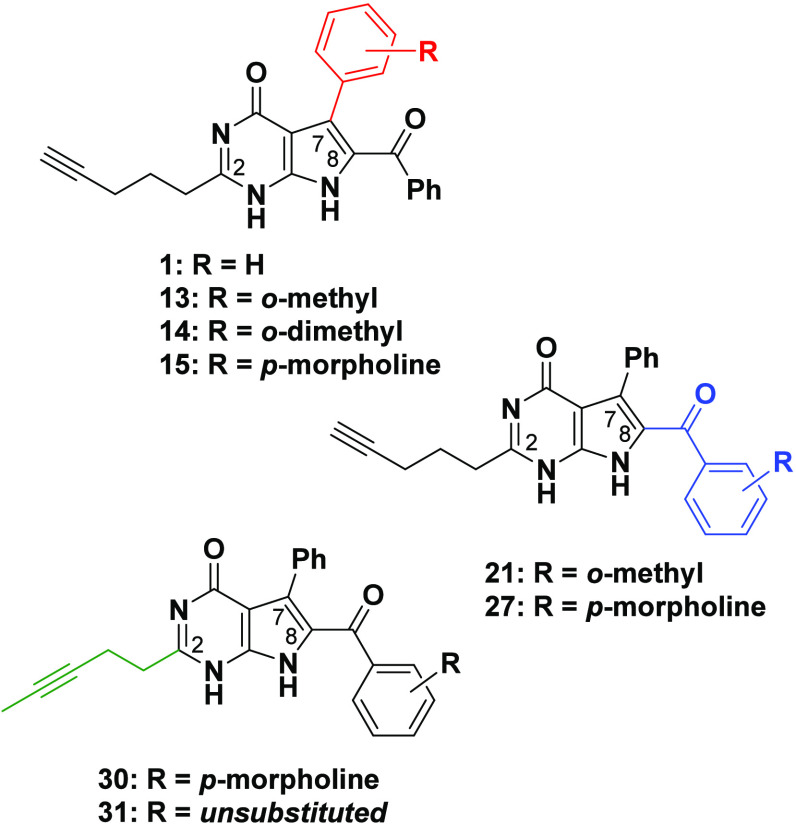
Antiproliferative Activities of 7-Deazahypoxanthine Analogues against HeLa Cells

**analogue**	**GI_50_ (μM)± SD**^a^
**1**	0.022 ± 0.002^b^
**13**	0.142 ± 0.044
**14**	2.30 ± 0.09
**15**	11.3 ± 0.65
**21**	0.062 ± 0.005
**27**	0.025 ± 0.002
**30**	0.043 ± 0.002
**31**	0.047 ± 0.002

a Concentration found to reduce the viability of HeLa cells by 50%; 48 h treatment period with respective compounds, relative to DMSO control. Data includes the average and standard deviation (SD), from two independent determinations, each performed in quadruplicate, as determined by the MTT assay.

b In agreement with results from our previous work.^19^

In contrast, the incorporation of substituents from the C8 sulfonamido acetophenone-derived component resulted in a retention of potent nanomolar activity. Analogues **21**, **27**, and **30**, bearing the *ortho*-methyl and *para*-morpholino substituents, displayed antiproliferative activity in the same range as lead compound **1**. Interestingly, the potency of compounds **27** and **30** demonstrated that the introduction of the relatively large morpholine group in the *para*-position could potentially be accommodated in the proposed binding pocket of β-tubulin. Moreover, a comparison of the terminal versus internal alkyne analogues (**1**, **27** vs **31**, **30**) showed a 2-fold reduction in activity following isomerization to the more stable internal alkyne. This may be ascribed to the increased side-chain rigidity upon alkyne internalization (see single-crystal X-ray structure in Figure 4b), which is expected to be less favored within the narrow channel of the β-tubulin’s binding domain.^18^ Gratifyingly, analogue **27**, bearing a *para*-morpholine at C8 and the terminal alkyne, displayed excellent antiproliferative activity (25 ± 2 nM), essentially being equipotent to the lead compound **1**, and this particular outcome inspired the design of a second set of target compounds.

### (a-ii) SAR-Guided Synthesis of a Second Set of 7-Deazahypoxanthine-Inspired Compounds containing WSGs Incorporated via a Sulfonamido Acetophenone-Derived Component in the MCR

**Figure 5:**
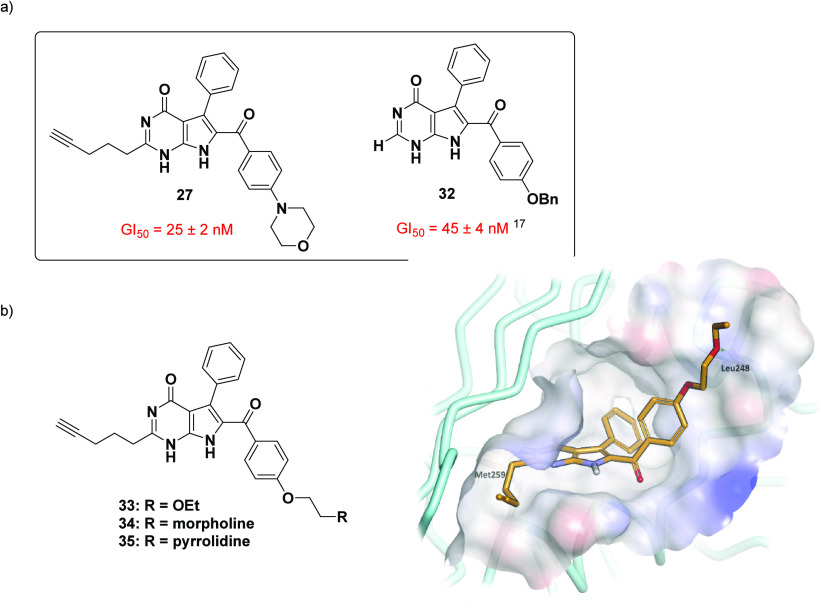
Structural representation of (a) potent 7-deazahypoxanthine analogues **27** and **32**;^17^ (b) chemical structure of the target compounds **33**–**35** bearing potential solubilizing appendages; molecular modeling of target **33** within the colchicine site of β-tubulin predicted that the orientation of the ethylene glycol ethyl ether would be toward the solvent exposed region.

Evaluation of the previously reported molecular modeling of the synthetic 7-deazahypoxanthines^19^ demonstrated the accommodation of the aryl groups in predominately hydrophobic pockets (see Figure 3 shown earlier). However, the sulfonamido acetophenone-derived (C8) aryl group was directed toward the solvent exposed region, rationalizing the tolerance in terms of the introduction of the *para*-morpholino appendage on analogues **27** and **30**. Building on this, interest was directed toward another rigidin-inspired 7-deazahypoxanthine (**32**), previously synthesized by Frolova et al.^17^ Compound **32** possessed a *para*-benzyl moiety and exhibited low nanomolar antiproliferative activity (GI_50_ = 45 ± 4 nM)^17^ (Figure 5). Encouraged by this potent activity, the introduction of solubilizing appendages at this position was envisaged, while maintaining the terminal alkyne alkyl chain. Therefore, motivated by the retention of activity displayed by the *para*-morpholine containing analogue **27** and the literature compound **32**,^17^ compared to the lead compound **1**, the synthesis of target compounds **33**–**35**, incorporating a proposed ethyl ether, morpholine or pyrrolidine solubilizing motif, was undertaken.

To verify whether the anticipated binding confirmation could be adopted by the new set of target compounds, the glycol ethyl ether analogue **33** was docked into the colchicine site of β-tubulin. In agreement with previous observations, molecular modeling suggested that the terminal alkyne alkyl chain would once again be accommodated in the hydrophobic groove (Asn258 and Lys352 amino acid chain channel). In addition, the modeling favored positioning the C7 phenyl group deeply within the binding pocket and that the *para*-ethylene glycol ethyl ether containing C8 aryl group would most likely be oriented toward the solvent exposed region (see Figure 5b).

**Scheme 5:**
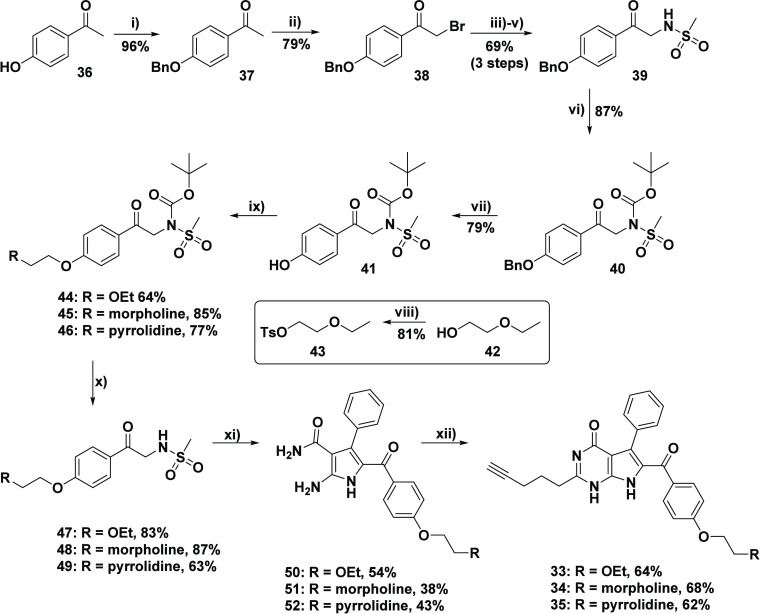
Synthesis of Target 7-Deazahypoxanthines **33**–**35** Possessing Solubilizing Appendages Reagents and conditions: (i) BnBr (1.1 equiv), K_2_CO_3_ (1.5 equiv), KI (0.2 equiv), acetone, RT, 12 h; (ii) PTAB (1 equiv), THF, RT, 1.5 h; (iii) hexamethylenetetramine (1.1 equiv), C_6_H_5_Cl, 30 °C, 6 h; (iv) EtOH/HCl (conc.), RT, 48 h; (v) MsCl (3 equiv), Et_3_N (5 equiv), acetone/H_2_O, 0 °C–RT, 12 h; (vi) Boc_2_O (1.2 equiv), DMAP (0.15 equiv), Et_3_N (1.1 equiv), THF, 0 °C–RT, 5 h; (vii) Pd/C (15 mol %), H_2_ (1 atm.), EtOAc, RT, 8 h; (viii) *p*-TsCl (1.1 equiv), Et_3_N (1.2 equiv), 0 °C–RT, 12 h; (ix) 2-chloro *N*-substituted ethanamine hydrochloride salt (2.5 equiv)/2-ethoxyethyl 4-methylbenzenesulfonate **43** (1.5 equiv), K_2_CO_3_ (2–3.5 equiv), KI (0.2 equiv), acetone, reflux, 24 h; (x) TFA/DCM (1:6), 0 °C–RT, 4 h; (xi) cyanoacetamide **4** (1.3 equiv), PhCHO **3** (1.3 equiv), K_2_CO_3_ (0.6 equiv), EtOH, reflux, 24 h; (xii) ethyl hex-5-ynoate **6** (10 equiv), 0.65 mM NaOEt (10 equiv of Na), 80 °C, 48–30 h.

To develop a route which would allow for synthetic diversification of the essential sulfonamido acetophenone components, a protecting group-assisted strategy was implemented (Scheme 5). Although this approach added additional reaction steps to the general strategy, the advantages offered by this route contributed to its synthetic feasibility, thereby justifying the lengthier path. This approach was further substantiated by the overall high yielding procedures developed earlier in the project. The first set of reactions relied on the use of a benzyl group to facilitate the synthesis of the mesylated species **39**. Therefore, the initial procedure involved benzyl protection of the 4-hydroxyacetophenone **36** affording **37**, followed by the general α-bromination (to give **38**), followed by Délepine and mesylation reactions to give **39**, as developed by Wang, Xiao and co-workers.^56^ In order to facilely introduce the solubilizing groups at the *para*-phenol position, the incorporation of a Boc-protecting group at the sulfonamide nitrogen was crucial. As such, the next two reactions involved a Boc-protection of the methanesulfonamide **40**, followed by benzyl-deprotection to expose the phenoxyl group in compound **41**. At this point, the various solubilizing groups could be introduced. To allow for introduction of a glycol ether moiety, commercially available 2-ethoxyethanol **42** was treated with *p*-toluenesulfonyl chloride to provide 2-ethoxyethyl 4-methylbenzenesulfonate **43** (see insert, Scheme 5). The solubilizing groups were then added by treatment of **41** with the respective 2-chloro *N*-substituted ethanamine hydrochloride salt or compound **43**. The reactions were carried out with potassium carbonate in acetone and heated to reflux and a catalytic amount of potassium iodide was added to promote the Finkelstein process. In this manner, the desired Boc-protected sulfonamido acetophenone analogues **44**–**46**, which possessed the desired solubilizing appendages, were obtained in satisfactory yields. Finally, the selective cleavage of the Boc group was achieved upon treatment of compounds **44**–**46** with TFA/DCM. This facile procedure generated the desired sulfonamido acetophenone components **47**–**49**. As for this process the reaction mixture could simply be concentrated *in vacuo*, and as such, the risk of losing material (water-soluble **48** and **49**) due to exposure to aqueous media during a workup procedure, was avoided. Overall, high yields were achieved for the Boc-deprotection steps and the successive products were fully characterized.

With the pure sulfonamido acetophenone components **47**–**49** in hand, the crucial MCR could be executed to assemble the respective pyrroles **50**–**52** in moderate yields. The final ring-closing reaction required the use of alkyne alkyl ester **6**, and allowed for construction of the pyrrolo[2,3-*d*]pyrimidine scaffolds bearing the desired terminal alkyne. Using the general procedure with NaOEt prepared *in situ* and heating to 80 °C, the target deazahypoxanthines **33**–**35** were furnished in reasonable yield and with high purity (95–98%) after silica gel column chromatography (Scheme 5). Notably, the respective pyrroles **50**–**53** did not experience the solubility challenges previously observed, therefore negating the need for DMSO and elevated temperatures which formerly led to alkyne isomerization issues.

**Scheme 6:**
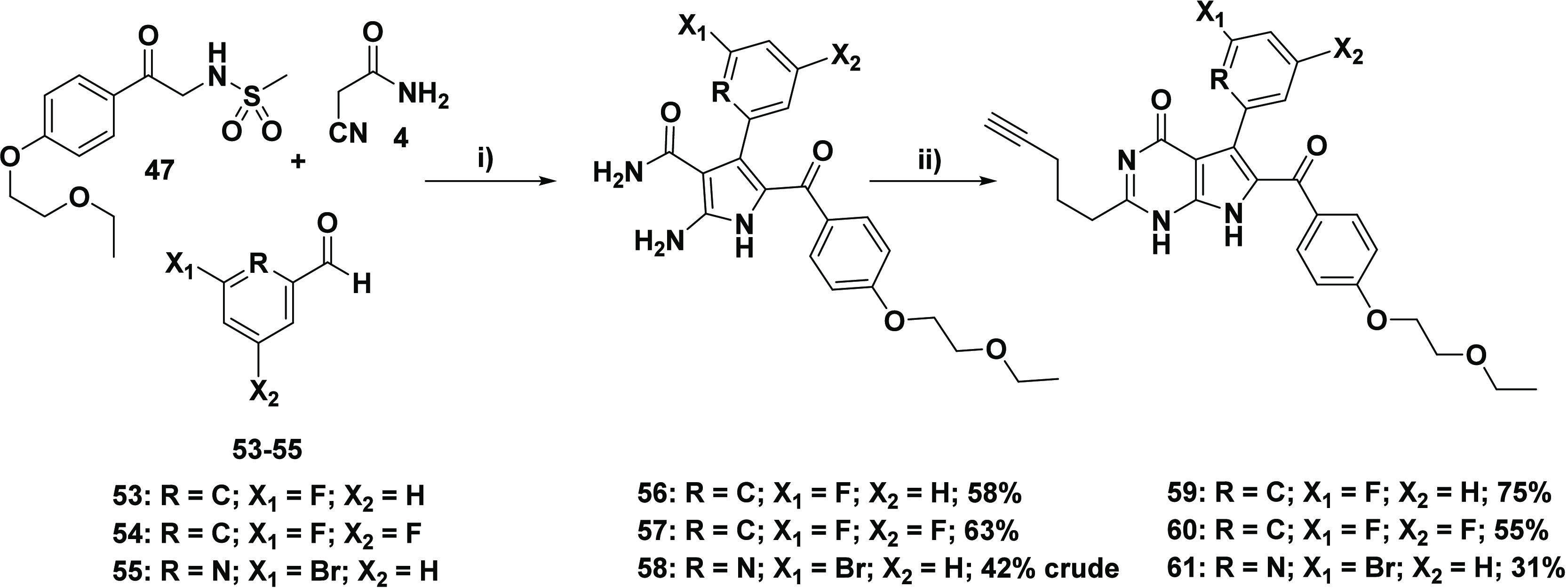
Synthesis of Target 7-Deazahypoxanthines **59**–**61** Reagents and conditions: (i) sulfonamido acetophenone **47**, cyanoacetamide **4** (1.3 equiv), substituted benzaldehyde **53/54/55** (1.3 equiv), K_2_CO_3_ (0.6 equiv), EtOH, reflux, 24 h; (ii) ethyl hex-5-ynoate **6** (10 equiv), 0.65 mM NaOEt (10 equiv), 80 °C, 24 h.

Furthermore, in continuation of our interest in the design of potent rigidin-inspired analogues with potential improved aqueous solubility, the derivatization of the C7 aryl-ring was also envisaged. As briefly alluded to by Frolova et al., the introduction of *meta*-halogens on the rigidin-framework phenyl group (that originates from the benzaldehyde component) could be beneficial for enhancing the solubility of these structures.^17^ Therefore, 3-fluorobenzaldehyde **53**, 3,5-difluorobenzaldehyde **54**, and 6-bromo-2-pyridine-carboxaldehyde **55** were selected for their incorporation onto the 7-deazahypoxanthine scaffold as advanced targets, while maintaining the favorable glycol ether moiety of **33**. Having the desired sulfonamido acetophenone component **47** in hand, the synthesis of the respective final compounds simply involved the MCR, followed by the second ring-closing reaction with ethyl hex-5-ynoate **6** (Scheme 6). These initial reactions were carried out seamlessly, affording the respective pyrrole intermediates **56** and **57** in a moderate yield and subsequently target compounds **59** and **60**. On the other hand, the lower yields obtained for the pyrrole **58** and 7-deazahypoxanthine **61** were attributed to potential instability of the 6-bromo-2-pyridine moiety, which was also presumed to be the culprit for the lower purity of **58**. As a result of this stability liability, target compound **61** was not evaluated in-depth. Compound **61** was tested once against the HeLa cell line, and an GI_50_ of 22.4 ± 3.8 nM was obtained (see Table 2 for related testing). However, due to clear degradation of the compound in solution, no further studies were done on this sample.

**Table 2:** Antiproliferative Activities of 7-Deazahypoxanthine Analogues against the HeLa Cell Line^f^

**analogue**	**GI**_**50**_ **(μM) ± SD**^a^
**1**	0.022 ± 0.002^b^,^c^
	0.061 ± 0.037^d^,^e^
**33**	0.015 ± 0.004^c^
**34**	0.444 ± 0.030^c^
**35**	2.865 ± 0.103^c^
**59**	0.016 ± 0.004^d^
**60**	0.022 ± 0.005^d^

a Concentration needed to inhibit the viability of HeLa cells by 50%. Refer to caption of Table 1 for more information.

b Initial activity of **1** from Table 1.

c Data includes the average and SD, from two independent determinations, each performed in quadruplicate.

d Average of eight repeats determined over 5 weeks.

e The large standard deviation is indicative of the poor reproducibility between assays, attributed to the peculiar aqueous solubility properties of compound **1**.

f Compound **61** was tested once against the HeLa cell line, and an GI_50_ of 22.4 ± 3.8 nM was obtained. Due to clear degradation of the compound in solution, no further studies were done on this sample.

### (b-ii) Antiproliferative Activities of Second Set of Compounds

**Figure 6:**
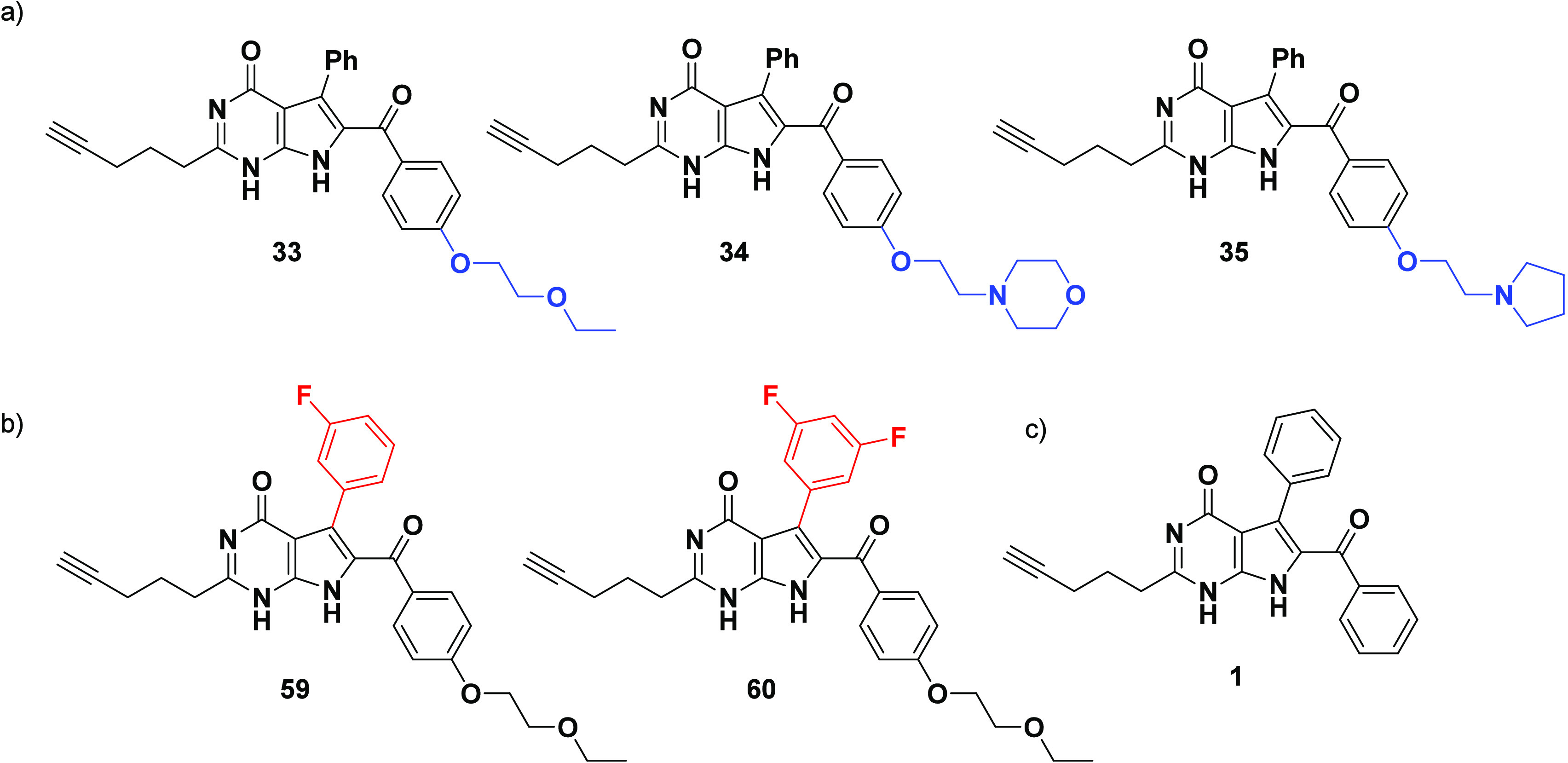
Chemical structures of target compounds; (a) **33**–**35** bearing a glycol ether and *N*-cyclic ethanamines (morpholine/pyrrolidine) as solubilizing appendages; (b) **59** and **60** optimized from derivative **33** to incorporate (di) *meta*-fluoro phenyl moiety; (c) Reference structure **1**.

Having successfully synthesized 7-deazahypoxanthines **33**–**35**, **59**, and **60** (Figure 6), their antiproliferative activities were evaluated against HeLa cancer cells, using an MTT proliferation assay,^55^ as presented in Table 2, and a time-resolved apoptosis assay (Figure S2) to directly assay for cytotoxicity. From the results shown, the activity of the 7-deazahypoxanthines **34** and **35**, bearing the *N*-cyclic ethanamines, proved to be rather disappointing. These compounds showed an approximate 20 and 150-fold reduction in antiproliferative activity, when compared to lead compound **1**, with the pyrrolidine-containing analogue displaying the greatest reduction. The *N*-cyclic ethanamines therefore did not function as desirable solubilizing appendages as their introduction onto the core scaffold sacrificed potency. We believe this to perhaps stem from unfavorable steric interactions of the larger *N*-cyclic groups interfering in the binding domain. Another rationale could be ascribed to the protonated morpholine/pyrrolidine (at physiological pH) not being tolerated within the active site. In addition, these charged entities could be interfering with the cellular permeability of these compounds. However, to our delight, the glycol ether solubilizing appendage-bearing 7-deazahypoxanthine **33** exhibited excellent antiproliferative activity of 15 nM, showing improved activity compared to our lead compound **1**. Indeed, this compound was also found to have the same GI_50_ value of 15 nM against the PANC-1 prostate cancer cell line (see Table S1). It was clear that in obtaining the cellular data, namely, 8 repetitions over 5 weeks, that compound **1**’s activity seemed to be effected negatively by repetitive rethawing from DMSO, while the new compounds fared much better in this regard (see data in Table 2).^57^ Initial aqueous solubility analysis of a subset of the compounds in hand, using laser nephelometry, indicated that most of them had a very similar solubility range (14–23 μM), apart from the amine-tethered compounds **34** and **35**, which were significantly more soluble. Due to the complex aspects involved in solubility, a better understanding of these properties will likely also require a thermodynamic investigation—this research will be included in the next stage of our project. Considering the potent activity and better compound behavior due to the presence of the solubilizing appendage, compound **33** therefore serves as the new front-runner in our research with regard to cancer cell cytotoxicity among this family of 7-deazahypoxanthines. In addition, as anticipated, the antiproliferative activity of the compounds having incorporated both the glycol ether at C8 and the C7 3-fluorobenzaldehyde **53**/3,5-difluorobenzaldehyde **54** was not jeopardized, with final compounds **59** and **60** retaining low nanomolar potencies comparable to **33**, while also behaving better in terms of the antiproliferative results obtained over the 5 weeks of testing (Table 2, last two entries). Compound **59**, essentially being equipotent to **33**, would thus be another compound to scrutinize in terms of potential for development. Direct detection of apoptosis using a time-resolved, high-throughput fluorescent annexin V binding assay showed slightly different trends among the lead compounds **1**, **33**, **59**, and **60**, but they were not statistically significant (Figure S2). A selection of the compounds **1**, **21**, **27**, **33**, **59**, and **60** were also tested for cytotoxicity against the normal epithelial cell line PNT1A and were found to have an approximate 2-fold therapeutic index, the most selective being compound **59** (Table S1). Of interest is that when compound **1** was previously tested for cytotoxicity against the WI38 embryonic lung fibroblast cell line, it was found to be approximately 1000-fold less active when compared to four colorectal cancer cell lines.^19^ The apparent selectivity differences of the synthetic rigidins for epithelial over fibroblast cells warrants further investigation.

### (c-i) Inhibition of Tubulin Assembly

**Figure 7:**
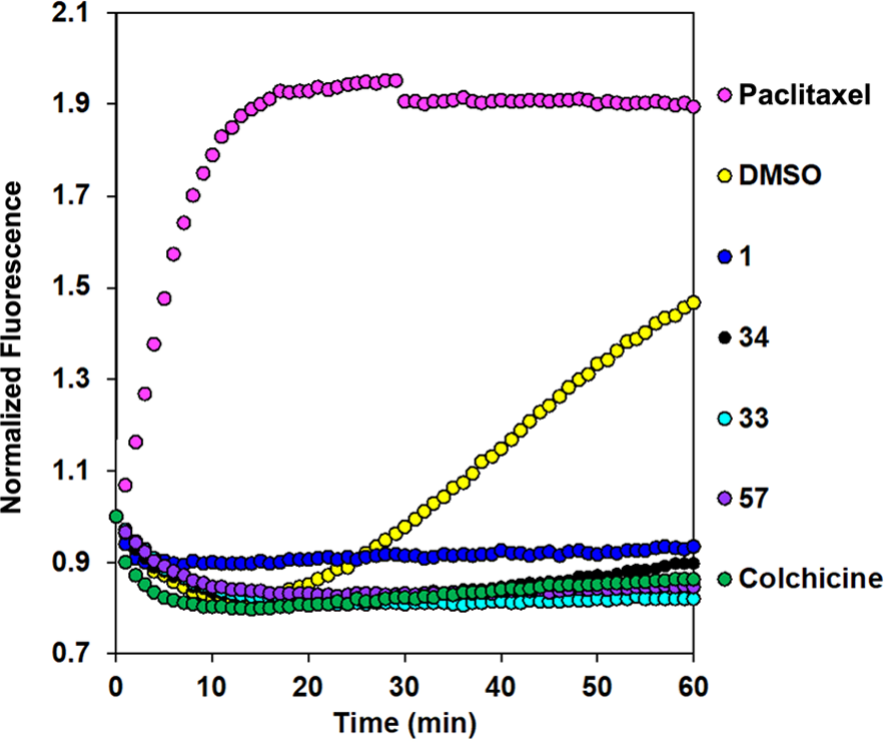
Effect of **1**, **33**, **34**, and **59** (averaged from two experiments) on in vitro tubulin polymerization. Paclitaxel (3 μM) promotes microtubule formation relative to 1% DMSO control. **1**, **33**, **34**, and **59** (all at 25 μM) and colchicine (6 μM) completely suppress tubulin polymerization.

To confirm that the mode of action had not changed with the structural variations in this series of rigidin analogues, the effects of some of them on microtubule polymerization were assessed.^58^ The test compounds, comprising samples **1**, **33**, and **59** and the less potent **34**, were compared to three controls: known microtubule stabilizer (paclitaxel) and destabilizer (colchicine), as well as a DMSO vehicle control. The data in Figure 7 clearly demonstrates that whereas paclitaxel induces potent enhancement of microtubule formation relative to the effect of the control, the rigidin analogues **1**, **33**, **34**, and **59** display potent inhibition of microtubule assembly in a manner similar to colchicine.

### (c-ii) Effects on Microtubule Organization

**Figure 8:**
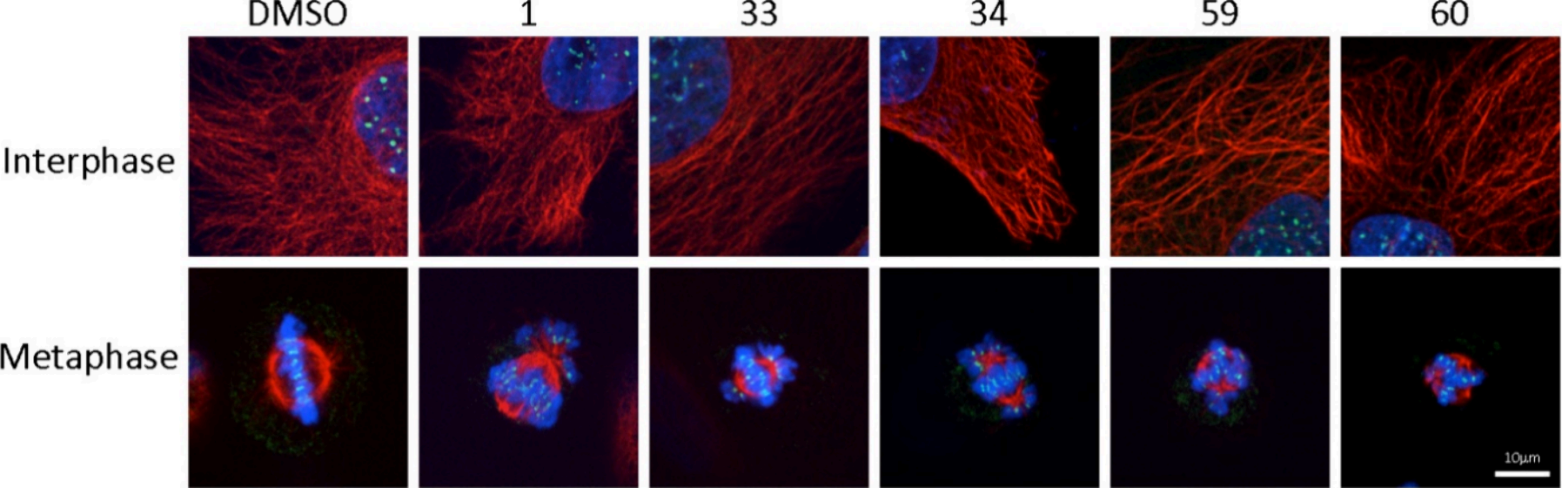
Interphase and mitotic microtubule organization in HeLa cells cultured in the presence of rigidin analogues. HeLa cells were treated for 4 h with compounds **1**, **33**, **34**, **59**, and **60** at their GI_50_ concentrations (20, 15, 450, 30, and 40 nM, respectively). Following drug treatment, cells were probed for microtubules (red), centromeres (green), and DNA (blue). Bar, 10 μm.

The synthesized rigidin analogues demonstrated excellent antiproliferative capabilities and disrupted microtubule polymerization (Table 2 and Figure 7, respectively). To confirm the biological effects of the tested compounds on the microtubule cytoskeleton, HeLa cells were treated with compound **1** and its selected analogues **33**, **34**, **59**, and **60** at a range of GI_50_ concentrations. While there were no striking effects on interphase microtubules (Figure 8, top row), all compounds tested had remarkable effects on the spindle morphology (Figure 8, bottom row). Spindle length was clearly decreased, and as reported previously for other 7-deazahypoxanthine analogues, spindle poles appeared to be differentially affected with one spindle pole of each spindle frequently lacking astral microtubules.^23^ Most notably, treated cells were characterized by paired sister chromatids clustered around the spindle poles. These effects were dose-dependent, with spindle morphology recovering in cells treated at lower concentrations (GI_25_), although the syntelic chromosome attachments were still prevalent in compounds **33** and **34** (Figure S3).

**Figure 9:**
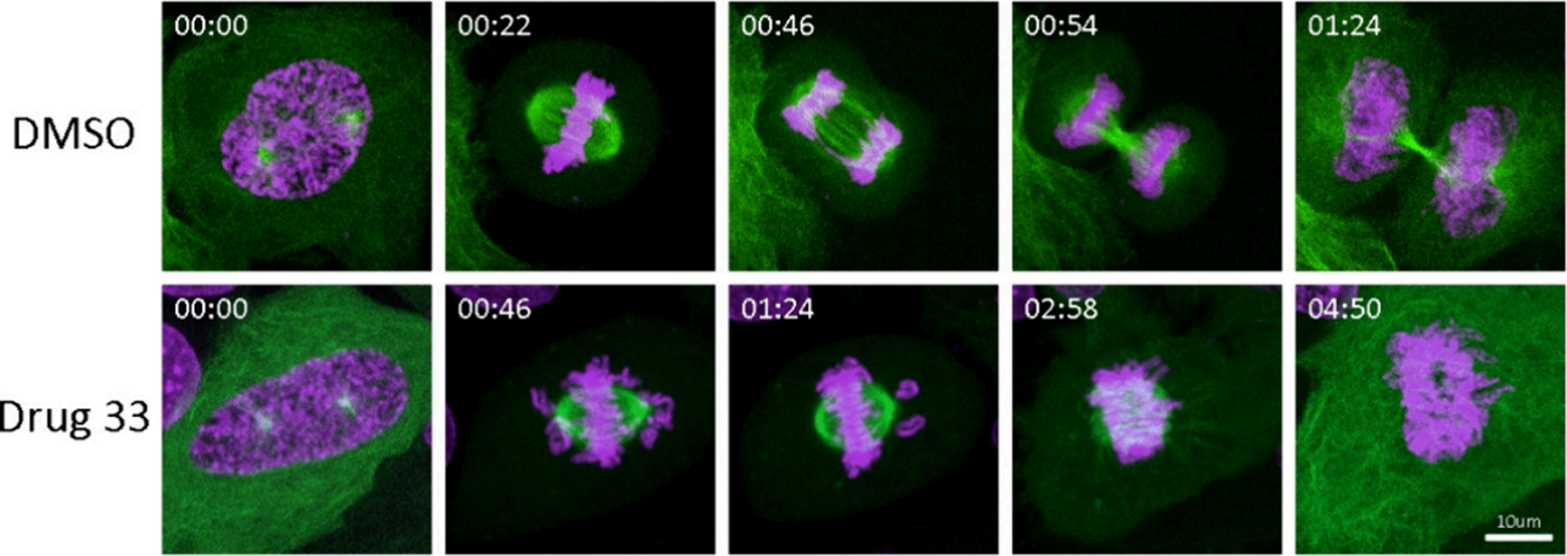
Time-lapse single cell analysis of HeLa cells undergoing mitosis. HeLa cells expressing EGFP-tubulin (green) were treated with either 0.1% DMSO (top row) or compound **33** at 7.5 nM (bottom row) for 4 h and stained for chromatin with NucSpot Live 650 (magenta) for 1 h prior to live-cell imaging. Image stacks (10 planes at 1.36 μm intervals) were acquired every 2 min, beginning at prophase. Time stamps begin at the initiation of nuclear envelope breakdown. Bar, 10 μm.

Antimitotic compounds act by stabilizing microtubule dynamics and activating the spindle assembly checkpoint;^59^ with a single monotelic or syntelic chromatid pair sufficient to activate the checkpoint.^60^ Given that the most common spindle defect identified by immunolabeling was the accumulation of chromosomes at the spindle poles, fluorescent EGFP-tubulin expressing HeLa cells were imaged live in the absence or presence of rigidin analogue **33**, at 50% of its determined GI_50_ concentration, to more carefully examine chromosome congression defects in treated cells. In controls, sister chromatid pairs rapidly congressed to the metaphase plate, and cells reached metaphase within 30 min (Figure 9, top row, Movie S1). In contrast, imaging of cells treated with compound **33** reflected various responses ranging from correcting the syntelic attachments and progressing through mitosis to prolonged prometaphase arrest, followed by mitotic exit and/or cell death (Figure 9 bottom row, Movie S2). Cells were able to correct most attachment errors over an extended period of time, but in the cell shown in Figure 9, two sister chromatid pairs could not re-establish proper microtubule attachments before the cell began to exit mitosis. Together, these data are consistent with the mechanism of taxanes and vinca drugs,^61,62^ where the stabilization of microtubule dynamics interferes with chromosomal congression, leading to mitotic delay and eventual cell death.

Results of immunolabeling (Figure 8) and live cell imaging (Figure 9) demonstrated that at the effective doses, rigidin analogues delayed mitosis by disrupting correction of mono- or syntelic attachments while not completely blocking spindle assembly. In addition, while long-term imaging using an annexin V-based apoptosis sensor indicated that cells underwent apoptosis in the presence of these compounds, quantification of annexin V fluorescence did not provide information how cells were dying in response to mitotic delay. Cancer cells commonly display a wide heterogeneity in response to mitotic arrest, not only between cell lines but also among individual cells.^63−66^ To better understand the phenotypes of cells treated with the lead compounds, the raw video data from the annexin V apoptosis assays (Figure S2) was mined to track individual cells over time to measure the frequency of different possible cell fates following mitotic arrest (Figure S4A). Analysis of >130 cells per treatment revealed that while cells were capable of either completing cell division or undergoing slippage back to interphase, the vast majority of cells that entered mitosis underwent apoptosis while still in mitosis (Figure S4B). In addition, while the total numbers of cells that died following mitotic arrest were similar for **1** (75%), **33** (86%), **59** (87%), and **60** (85%), compounds **1**, **59**, and **60** had higher levels of cells that either completed cytokinesis or slipped back into interphase before undergoing apoptosis (Figure S4B), providing further supporting evidence that **33** might be a more efficient cytotoxic agent.

## Conclusions

In this work, recognizing that a limitation of the previous rigidin mimic lead **1** was its poor water solubility, several new rigidin-inspired compounds were synthesized by way of a multicomponent reaction approach. After the development of two small libraries, it was found that molecules with a water-solubilizing glycol ether functional group on the C8 aryl ring system of the 7-deazahypoxanthine provided a very potent framework **33** with antiproliferative activities against HeLa cells in the low double digit nanomolar region. This scaffold also tolerated small fluoro-substituents on the C7-aromatic ring in that compounds **59** and **60** were also equipotent. From the antiproliferative cellular experiments, it was clear that the glycol ether functional group on the C8 aryl ring improved the solubility behavior of the compounds. The new compounds were shown to potently inhibit tubulin polymerization in an in vitro assay and as visualized by live cell confocal microscopy. These potent rigidin mimics have potent antiproliferative activity by impacting the cellular spindle morphology of HeLa cells, leading to mitotic delay and cell death. Future studies on this family of 7-deazahypoxanthine rigidin mimics will include further investigative approaches to ensure these potent antiproliferative agents are suitable as further leads to be evaluated in vivo.

## Experimental Section

### General Practices: Synthesis

Reactions were carried out in a fume hood and performed under a positive pressure of 2.8 kPa of 5.0 grade nitrogen or argon, unless otherwise stated. Glassware was oven-dried, placed under vacuum (∼ 0.8 mmHg), purged with nitrogen/argon, and evacuated until room temperature was reached.

### Solvents and Reagents

Reagents were purchased from Merck or Sigma-Aldrich and used without further purification, unless otherwise noted. Solvents used for reaction purposes were dried over the required drying agents and then distilled under nitrogen gas. Tetrahydrofuran (THF) was distilled from sodium wire using benzophenone as an indicator. Ethanol was freshly distilled from magnesium turnings and catalytic iodine. Solvents used for flash column chromatography was distilled in bulk under an open atmosphere. The MCRs required the use of granular K_2_CO_3_ and dried overnight at 120 °C. All other reagents that required purification were purified according to standard procedures.^1^

### Chromatography and Purification

Thin layer chromatography (TLC) was performed on Macherey Nagel aluminum TLC-plates, precoated with 0.20 mm silica gel and fluorescent indicator UV_254_. Compounds were visualized with UV light (λ = 254 nm) or KMnO_4_ stain followed by heating. pH was determined using universal pH test paper strips. Flash column chromatography was performed using Merck silica gel 60 (particle size 0.040–0.063 mm). Preparative TLC glass plates (silica gel 60 F254, 1 mm) were employed for small-scale purifications. Mobile phase and *R*_f_ value solvent ratios are reported as v/v ratios. A rotary evaporator was used to remove solvents in vacuo. Products were dried under high vacuum (∼0.8 mmHg). Purity was measured by the Central Analytical Facility (CAF) of Stellenbosch University, using a Waters Acquity UPLC system fitted with a photodiode array detector (system: Waters BEH C18 column (1.7 μm, 100 mm × 2.1 mm).

### Spectroscopic and Physical Analysis

Infrared (IR) spectra were recorded on a Thermo Nicolet FT-IR, using an Attenuated Total Reflectance (ATR) attachment. OMNIC 7.0 software was used for spectral analysis. NMR spectra were recorded at 25 °C on a 300 MHz Varian VNMRS (75 MHz for ^13^C NMR spectra), 400 MHz Varian Unity Inova (101 MHz for ^13^C NMR spectra), or 600 MHz Varian Unity Inova (151 MHz for ^13^C NMR spectra) at the CAF of Stellenbosch University.

Samples were dissolved in deuterated chloroform (CDCl_3_), dimethyl sulfoxide (DMSO-*d*_6_), and methanol (CD_3_OD). Chemical shifts (δ) are reported in parts per million (ppm). Signal multiplicities are indicated as br s (broad singlet), s (singlet), d (doublet), dd (doublet of doublets), t (triplet), q (quartet), p (pentet), and m (multiplet), and coupling constants (*J*) are expressed in Hertz (Hz). ^1^H and ^13^C spectra are referenced to the residual solvent signal DMSO-*d*_6_ (2.50 or 39.52 ppm), CDCl_3_ (7.26 or 77.16 ppm), or CD_3_OD (1.94 or 49.00 ppm). In cases were the CDCl_3_ solvent peak was masked by other peaks, the spectra were referenced to the internal standard tetramethylsilane (TMS) peak at δ 0.00 ppm. Although, DMSO-*d*_6_ was dried over 4 Å molecular sieves, and all ^1^H NMR spectra display a water peak at δ 3.33 ppm. NMR spectra were processed in MestreNova 11.0.2. Melting points were obtained using Lasany Melting Point Apparatus. High-resolution mass spectrometry was performed by the CAF at Stellenbosch University, utilizing a Waters SYNAPT G2 QTOF spectrometer, in ESI-positive mode.

#### *N*-(2-Oxo-2-phenylethyl)methanesulfonamide **2**

A 2-neck round-bottom flask was charged with commercially available 2-aminoacetophenone hydrochloride (3.00 g, 17.5 mmol, 1 equiv) and a mixture of acetone/H_2_O (60 mL/12 mL). Methanesulfonyl chloride (2.98 mL, 38.5 mmol, 2.2 equiv) was then added in one portion and the reaction mixture was subsequently cooled to 0 °C, followed by the slow dropwise addition of Et_3_N (8.56 mL, 61.2 mmol, 3.5 equiv). The resulting solution was removed from the icebath, and an additional volume of acetone (60 mL) was added. The reaction mixture was stirred at RT for 24 h during which time the solution changed in color from pink/orange to yellow. Upon completion, the acetone was removed under reduced pressure. The resulting slurry was then partitioned between EtOAc (100 mL) and a saturated solution of NH_4_Cl (80 mL). The organic layer was collected and washed with a saturated solution of NaHCO_3_ (80 mL), brine, and dried over MgSO_4_. The drying salts were filtered, and the solvent removed in vacuo. Purification was achieved by column chromatography (EtOAc/Hexane, 2:6 to 4:1), affording the desired product as a white solid (3.29 g, 88%). The NMR spectra of *N*-(2-oxo-2-phenylethyl)methanesulfonamide **2** was compared to that reported in the literature and corresponded well.^10^

***R*_f_** = 0.42 (EtOAc/hexane, 1:1); ^**1**^**H NMR (300 MHz, CDCl**_**3**_**) δ** 7.98–7.92 (m, 2H, 2 × Ar*H*), 7.68–7.62 (m, 1H, Ar*H*), 7.55–7.48 (m, 2H, 2 × Ar*H*), 5.42 (brs, 1H, N*H*), 4.68 (d, *J* = 4.8 Hz, 2H, C*H*_2_), 3.01 (s, 3H, C*H*_3_).

#### Ethyl Hex-5-ynoate **6**

To a 2-neck round-bottom flask was added 5-hexynoic acid (2.00 g, 17.8 mmol, 1 equiv) and DMF (40 mL), the resulting solution was then treated with K_2_CO_3_ (3.69 g, 26.7 mmol, 1.5 equiv) and ethyl bromide (1.99 mL, 26.7 mmol, 1.5 equiv) successively. The mixture was stirred at RT overnight. Upon completion, the reaction mixture was partitioned between H_2_O (20 mL) and diethyl ether (3 × 15 mL). The combined organic extracts were collected and washed with brine and dried over MgSO_4_. The drying salts were filtered, and the solvent removed in vacuo. Purification by flash column chromatography (diethyl ether/Hexane, 0.5:9.5) afforded ethyl hex-5-ynoate **6** (2.07g, 83%) as a colorless oil. The NMR spectra of **6** was compared to that reported in the literature and corresponded well.^11^

***R***_**f**_ = 0.47 (diethyl ether/hexane, 1:9); ^**1**^**H NMR (300 MHz, CDCl**_**3**_**) δ** 4.13 (q, *J* = 7.1 Hz, 2H, OC*H*_2_CH_3_), 2.43 (t, *J* = 7.4 Hz, 2H, C*H*_2_CH_2_CH_2_CCH), 2.26 (td, *J* = 7.2, 2.7 Hz, 2H, CH_2_CH_2_C*H*_2_CCH), 1.96 (t, *J* = 2.7 Hz, 1H, CC*H*), 1.90–1.78 (m [app. p], *J* = 7.2 Hz, 2H, CH_2_C*H*_2_CH_2_CCH), 1.25 (t, *J* = 7.1 Hz, 3H, OCH_2_C*H*_3_); ^**13**^**C NMR (75 MHz, CDCl**_**3**_**) δ** 173.2, 83.4, 69.2, 60.5, 33.1, 23.8, 18.0, 14.4.

### General Procedure A for the Synthesis of Tetrasubstituted 2-Aminopyrroles (Compounds **5**, **10**, **11**, **12**, **20**, **26**, **50**, **51**, **52**, **56**, **57**, and **58**)

A 2-neck pear-shaped flask was charged with the methanesulfonamide component (1 equiv), cyanoacetamide (1.3 equiv), and anhydrous granulated K_2_CO_3_ (0.5 or 0.6 equiv). The benzaldehyde component (1.3 equiv) was then added with EtOH. Note, for small-scale MCRs, a microwave vial was used instead, which was flushed with argon after addition of all reagents and clamped. The resulting reaction mixture was heated under reflux and stirred for 24 h. The reaction flask was then placed in a fridge overnight to promote precipitate formation. The precipitate was collected and purified via column chromatography. In cases where no precipitation occurred, the filtrate solvent was removed under reduced pressure and purified by way of column chromatography (although this proved to be more challenging).

#### 2-Amino-5-benzoyl-4-phenyl-1*H*-pyrrole-3-carboxamide **5**

The desired product was prepared according to the general procedure **A** with the following specific amounts: *N*-(2-oxo-2-phenylethyl)methanesulfonamide **7** (1.50 g, 7.03 mmol, 1 equiv), benzaldehyde (0.970 g, 9.14 mmol, 1.3 equiv), cyanoacetamide (0.769 g, 9.14 mmol, 1.3 equiv), and anhydrous granular K_2_CO_3_ (0.486 g, 3.52 mmol, 0.5 equiv) in EtOH (65 mL). After the reaction, the precipitate was collected and washed with EtOH/MeOH (5 mL/5 mL) resulting in a yellow solid. The desired product 2-amino-5-benzoyl-4-phenyl-1*H*-pyrrole-3-carboxamide **5** (1.32 g, 61%) was obtained as a yellow solid and required no further purification. The NMR spectra of 2-amino-5-benzoyl-4-phenyl-1*H*-pyrrole-3-carboxamide **5** were compared to that reported in the literature and corresponded well.^12^

***R***_**f**_ = 0.43 (EtOAc); ^**1**^**H NMR (300 MHz, DMSO-*d*_6_) δ** 10.91 (br s, 1H, N*H*), 7.20–7.01 (m, 8H, 8 × Ar*H*), 7.01–6.91 (m, 2H, 2 × Ar*H*), 6.74 (br s, 1H, N*H*_2_), 6.39 (s, 2H, N*H*_2_) 4.61 (br s, 1H, N*H*_2_); ^**13**^**C NMR (75 MHz, DMSO-*d*_6_) δ** 183.8 (*C*O), 167.2, 149.4, 139.2, 134.1, 132.1, 130.4 (2 × Ar*C*), 129.4, 127.9 (2 × Ar*C*), 127.9 (2 × Ar*C*), 127.5, 127.0 (2 × Ar*C*), 121.0, 99.0.

### General Procedure B for the Synthesis of 7-Deazahypoxanthines (Compounds **1**, **13**, **14**, **15**, **21**, **30**, **31**, **27**, **33**, **34**, **35**, **59**, **60**, and **61**)

A stock solution of NaOEt (0.65 mM) was prepared by dissolving Na (0.240 g, 10.4 mmol) in EtOH (16 mL). To a microwave vial charged with tetrasubstituted 2-aminopyrrole (1 equiv) was added Na (10 equiv) in EtOH from the NaOEt (0.65 mM) stock solution. The selected ethyl ester (8 or 10 equiv) was then added to the reaction solution. Finally, the microwave vial was purged with Ar, clamped, and then stirred at 80 °C for 24–30 h. Upon completion, H_2_O was added, and the reaction was neutralized using 2 M HCl and cooled to promote precipitate formation. In cases where precipitate formed, the precipitate was collected and purfied by column chromatography. During reactions where minimal precipitate was formed, the EtOH was removed under reduced pressure and DCM was added. The aqueous layer was then extracted twice with DCM. The organic layers were collected, washed with brine, and dried over MgSO_4_. The drying salts were then filtered and purification by column chromatography afforded the desired product.

#### 6-Benzoyl-2-(pent-4-yn-1-yl)-5-phenyl-1,7-dihydro-4*H*-pyrrolo[2,3-*d*]pyrimidin-4-one **1**

The desired product was prepared in a 2-neck round-bottom flask according to the general procedure **B** with the following specific amounts: 2-amino-5-benzoyl-4-phenyl-1*H*-pyrrole-3-carboxamide **5** (0.408 g, 1.34 mmol, 1 equiv), Na (0.308 g, 13.4 mmol, 10 equiv), 0.65 mM NaOEt (20.6 mL, 13.4 mmol), and ethyl hex-5-ynoate **6** (1.87 g, 13.4 mmol, 10 equiv). Yellow solid was precipitated from the reaction mixture. TLC indicated that some starting material remained unreacted even at longer reaction times. After 24 h of stirring, the reaction mixture was diluted with H_2_O (20 mL) and the precipitate went into solution. Precipitate formed again once the reaction solution was neutralized using 2 M HCl. The precipitate was collected and purified by column chromatography (EtOAc/Hexane/DCM/MeOH, 2.5:6:1.4:0.1) to afford 6-benzoyl-2-(pent-4-yn-1-yl)-5-phenyl-1,7-dihydro-4*H*-pyrrolo[2,3-*d*]pyrimidin-4-one **1** (0.419 g, 82%) as an off-white solid. NMR spectra of **1** was compared to that reported in the literature and corresponded well.^12^

***R***_**f**_ = 0.49 (EtOAc/hexane, 3:2); **MP** decomposes at ≥258 °C; ^**1**^**H NMR (300 MHz, DMSO-*d*_6_) δ** 12.61 (br s, 1H, N*H*), 11.91 (br s, 1H, N*H*), 7.41 (d, *J* = 8.4 Hz, 2H, 2 × Ar*H*), 7.34–7.27 (m [app. t], *J* = 7.4 Hz, 1H, Ar*H*), 7.20–7.08 (m, 4H, 4 × Ar*H*), 7.06–6.97 (m, 3H, 3 × Ar*H*), 2.83 (t, *J* = 2.6 Hz, 1H, CC*H*), 2.72 (t, *J* = 7.4 Hz, 2H, C*H*_2_CH_2_CH_2_CCH), 2.28 (td, *J* = 7.2, 2.6 Hz, 2H, CH_2_CH_2_C*H*_2_CCH), 1.98–1.85 (m [app. p], *J* = 7.2 Hz, 2H, CH_2_C*H*_2_CH_2_CCH); ^**13**^**C NMR (75 MHz, DMSO-*d*_6_) δ** 187.6 (CO), 159.3, 158.8, 149.8, 137.5, 132.6, 131.7, 131.1 (2 × Ar*C*), 129.0 (2 × Ar*C*), 127.6 (2 × Ar*C*), 127.2, 126.8, 126.8 (2 × Ar*C*), 126.6, 104.5, 83.9, 71.8, 32.9, 25.7, 17.3; **HRMS***m***/***z* (ESI^+^) calcd for C_24_H_20_N_3_O_2_ [M + H]^+^ 382.1556; found, 382.1537.

#### 2-Amino-5-benzoyl-4-(*o*-tolyl)-1*H*-pyrrole-3-carboxamide **10** and 2-Amino-5-benzoyl-1-(methylsulfonyl)-4-(*o*-tolyl)-1*H*-pyrrole-3-carboxamide **10b**

The desired product was prepared according to the general procedure **A** with the following specific amounts: *N*-(2-oxo-2-phenylethyl)methanesulfonamide **2** (0.100 g, 0.469 mmol, 1 equiv), 2-methylbenzaldehyde **7** (0.073 g, 0.61 mmol, 1.3 equiv), cyanoacetamide **4** (0.051 g, 0.61 mmol, 1.3 equiv), and anhydrous granular K_2_CO_3_ (0.039 g, 0.28 mmol, 0.6 equiv) in EtOH (6 mL). The reaction mixture appeared yellow in color with some precipitate evident. TLC analysis showed that both precipitate and filtrate contained product. The precipitate was collected and washed with EtOH/MeOH (1 mL/1 mL) resulting in an off-white solid after drying. The yellow filtrate was reduced in vacuo and purified by flash column chromatography (EtOAc/hexane/DCM/MeOH, 1.5:1.4:2:0.1). Purification proved to be very challenging. Several pyrrole intermediates, having similar *R*_f_ values, were isolated together with the mesylated-pyrrole, 2-amino-5-benzoyl-1-(methylsulfonyl)-4-(*o*-tolyl)-1*H*-pyrrole-3-carboxamide **10b**. The off-white solid obtained from the reaction precipitate and that provided after the filtrate purification were combined (0.062 g, 41%) and used in the next step toward the final product.

The *R*_f_ values of the product spots were very close, thereby complicating isolation of pure products. It must be noted that for this reaction the sample was not completely pure but comprised of a mixture of the major product **10** and **10b** (∼7.3:2.7).

#### 2-Amino-5-benzoyl-4-(*o*-tolyl)-1*H*-pyrrole-3-carboxamide **10**

**HRMS***m***/***z* (ESI^+^) calcd for C_19_H_18_N_3_O_2_ (M+H^+^) 320.1399; found, 320.1405.

#### 2-Amino-5-benzoyl-1-(methylsulfonyl)-4-(*o*-tolyl)-1*H*-pyrrole-3-carboxamide **10b**

***R***_**f**_ = 0.50 (EtOAc/DCM/MeOH, 2.9:2:0.1); ^**1**^**H NMR (300 MHz, DMSO-*d*_6_) δ** 10.91 (s, 1H, N*H*), 7.68–7.44 (m, 2H, 2 × Ar*H*), 7.29–6.90 (m, 7H, 7 × Ar*H*), 6.43 (s, 2H, N*H*_2_), 2.21 (s, 3H, C*H*_3_), 2.01 (s, 3H, C*H*_3_); ^**13**^**C NMR (75 MHz, DMSO) δ** 183.6 (*C*O), 167.2, 149.7, 139.2, 133.9, 131.2, 130.7, 129.7, 129.4, 128.0, 127.2 (2 × Ar*C*), 126.9 (2 × Ar*C*), 125.5, 125.2, 120.7, 98.6, 41.2 (*C*H_3_) 19.6 (*C*H_3_); **HRMS***m***/***z* (ESI^+^) calcd for C_20_H_22_N_3_O_4_S (M+H^+^) 400.1331; found, 400.1336.

#### 6-Benzoyl-2-(pent-4-yn-1-yl)-5-(*o*-tolyl)-1,7-dihydro-4*H*-pyrrolo[2,3-*d*]pyrimidin-4-one **13**

The desired product was prepared according to the general procedure **B** with some modifications. The following amounts were utilized: 2-amino-5-benzoyl-4-(*o*-tolyl)-1*H*-pyrrole-3-carboxamide (crude mixture **10** and **10b**, (∼7.3:2.7) (0.120 g, 0.302 mmol, 1 equiv), Na (0.172 g, 7.52 mmol, 25 equiv) from 1.30 mM NaOEt (5.7 mL, 7.5 mmol) stock solution, and ethyl hex-5-ynoate **6** (0.421 g, 3.01 mmol, 10 equiv). The reaction mixture was stirred at 80 °C for 48 h. Upon completion, the mixture was diluted with H_2_O (15 mL) and neutralized using 2 M HCl. Precipitate formation resulted upon neutralization. The precipitate was collected and washed with hexane (10 mL) and dried in vacuo. The reaction filtrate showed the presence of unreacted starting material **10**/**10b** by TLC. Purification of the precipitate was performed by flash column chromatography (EtOAc/hexane/DCM/MeOH, 1:3:0.9:0.1) and afforded 6-benzoyl-2-(pent-4-yn-1-yl)-5-(*o*-tolyl)-1,7-dihydro-4*H*-pyrrolo[2,3-*d*]pyrimidin-4-one **13** (0.073 mg, 61%) as an off-white solid.

***R*_f_** = 0.33 (EtOAc/hexane/DCM/MeOH, 1:3:0.9:0.1); **MP** decomposes at ≥260 °C; ^**1**^**H NMR (300 MHz, DMSO**-***d***_**6**_**) δ** 12.59 (br s, 1H, N*H*), 11.89 (br s, 1H, N*H*), 7.35 (d, *J* = 7.6 Hz, 2H, 2 × Ar*H*), 7.31–7.23 (m [app. t], *J* = 7.4 Hz, 1H, Ar*H*), 7.14–7.05 (m [app.t], *J* = 7.6 Hz, 2H, 2 × Ar*H*), 7.01–6.88 (m, 2H, 2 × Ar*H*), 6.82–6.71 (m, 2H, 2 × Ar*H*), 2.83 (t, *J* = 2.6 Hz, 1H, CC*H*), 2.71 (t, *J* = 7.4 Hz, 2H, C*H*_2_CH_2_CH_2_CCH), 2.32–2.24 (m, 2H, CH_2_CH_2_C*H*_2_CCH), 2.15 (s, 3H, C*H*_3_), 1.97–1.85 (m [app. p], *J* = 7.3 Hz, 2H, CH_2_C*H*_2_CH_2_CCH); ^**1**^**C NMR (75 MHz, DMSO-*****d***_**6**_**) δ** 187.3 (CO), 159.2, 159.0, 149.8, 137.8, 136.9, 133.1, 131.4, 131.4, 128.9, 128.4 (2 × Ar*C*), 127.6, 127.4 (2 × Ar*C*), 127.0, 126.1, 124.3, 105.9, 83.9, 71.8, 33.0, 25.6, 19.9, 17.3; **HRMS***m***/***z* (ESI^+^) calcd for C_25_H_22_N_3_O_2_ [M + H]^+^ 396.1712; found, 396.1700; purity: 93.5%

#### 2-Amino-5-benzoyl-4-(2,6-dimethylphenyl)-1*H*-pyrrole-3-carboxamide **11**

The desired product was prepared according to the general procedure **A** with the following specific amounts: *N*-(2-oxo-2-phenylethyl)methanesulfonamide **2** (0.100 g, 0.469 mmol, 1 equiv), 2,6-dimethylbenzaldehyde **8** (0.082 g, 0.61 mmol, 1.3 equiv), cyanoacetamide **4** (0.051 g, 0.61 mmol, 1.3 equiv), and anhydrous granular K_2_CO_3_ (0.039 g, 0.28 mmol, 0.6 equiv) in EtOH (6 mL). TLC indicated many side products generated during reaction. Only a small amount of precipitate was observed. Solvent was reduced in vacuo and material purified by flash column chromatography (EtOAc/hexane/DCM/MeOH, 3:1:0.9:0.1). The desired product 2-amino-5-benzoyl-4-(2,6-dimethylphenyl)-1*H*-pyrrole-3-carboxamide **11** (0.032 g, 21%) was obtained as a yellow/orange solid together with the intermediate 2-cyano-3-(2,6-dimethylphenyl)acrylamide **11b** (0.027 g, 29%) as a light yellow solid.

***R***_**f**_ = 0.42 (EtOAc/DCM/MeOH, 2:2:1);^**1**^**H NMR (400 MHz, DMSO**-***d***_**6**_**) δ** 10.86 (br s, 1H, N*H*), 7.16–7.09 (m, 3H, 3 × Ar*H*), 7.02–6.93 (m, 3H, 3 × Ar*H*), 6.85 (m, 2H, 2 × Ar*H*), 6.43 (s, 2H, N*H*_2_), 2.91 (s, 6H, 2 × C*H*_3_); ^**13**^**C NMR (101 MHz, DMSO-***d*_**6**_**) δ** 183.4 (*C*O), 167.2, 150.2, 139.3, 136.4 (2 × Ar*C*), 133.7, 130.1, 129.4, 127.9, 127.3 (2 × Ar*C*), 126.9 (2 × Ar*C*), 126.4 (2 × Ar*C*), 119.8, 97.6, 59.8, 20.1 (2 × *C*H_3_). Too many carbons. Very difficult to purify. **HRMS***m***/***z* (ESI^+^) calcd for C_20_H_20_N_3_O_2_ [M + H]^+^ 334.1556; found, 334.1561.

#### 2-Cyano-3-(2,6-dimethylphenyl)acrylamide **11b**

***R***_**f**_ = 0.36 (EtOAc/hexane/DCM/MeOH, 3:6:0.9:0.1); ^**1**^**H NMR (400 MHz, DMSO-*****d***_**6**_**) δ** 8.30 (s, 1H, C*H*), 8.05 (s, 1H, N*H*_2_), 7.82 (s, 1H, N*H*_2_), 7.24 (dd, *J* = 8.3, 6.8 Hz, 1H, Ar*H*), 7.14 (d, *J* = 7.6 Hz, 2H, 2 × Ar*H*), 2.22 (s, 6H, 2 × C*H*_3_); ^**13**^**C NMR (101 MHz, DMSO-*****d***_**6**_**) δ** 161.9, 152.6, 135.3 (2 × Ar*C*), 132.5, 129.1, 127.8 (2 × Ar*C*), 115.2, 115.1, 19.7 (2 × *C*H_3_).

#### 6-Benzoyl-5-(2,6-dimethylphenyl)-2-(pent-4-yn-1-yl)-1,7-dihydro-4*H*-pyrrolo[2,3-*d*]pyrimidin-4-one **14**

The desired product was prepared according to the general procedure **B** with some modifications. The following amounts were utilized: 2-amino-5-benzoyl-4-(2,6-dimethylphenyl)-1*H*-pyrrole-3-carboxamide **11** (0.100 g, 0.300 mmol, 1 equiv), Na (0.138 g, 6.00 mmol, 20 equiv) from 1.30 mM NaOEt (4.6 mL, 6.0 mmol) stock solution, and ethyl hex-5-ynoate **6** (0.336 g, 2.40 mmol, 8 equiv). The reaction mixture was stirred at 80 °C for 40 h. Upon completion, the reaction mixture was diluted with H_2_O (10 mL) and neutralized using 2 M HCl. Since precipitate formation was minimal, the volatile solvent was removed in vacuo, and an extraction with DCM (15 mL) was performed. The organic extract was collected, washed with brine, and dried over MgSO_4_. The drying salts were filtered, and the solvent was removed in vacuo. Purification by flash column chromatography (EtOAc/hexane/DCM/MeOH, 2:7:0.9:0.1) afforded 6-benzoyl-5-(2,6-dimethylphenyl)-2-(pent-4-yn-1-yl)-1,7-dihydro-4*H*-pyrrolo[2,3-*d*]pyrimidin-4-one **14** (0.071 mg, 58%) as an off-white solid.

***R***_**f**_ = 0.67 (EtOAc/Hexane/DCM/MeOH, 1:1.8:2:0.2); **MP** 225–230 °C; ^**1**^**H NMR (300 MHz, DMSO-*****d***_**6**_**) δ** 12.58 (br s, 1H, N*H*), 11.87 (br s, 1H, N*H*), 7.35 (d, *J* = 8.0 Hz, 2H, 2 × Ar*H*), 7.33–7.25 (m [app. t], *J* = 7.4 Hz, 1H, Ar*H*), 7.16–7.08 (m [app. t], *J* = 7.6 Hz, 2H, 2 × Ar*H*), 6.85 (dd, *J* = 8.2, 6.5 Hz, 1H, Ar*H*), 6.75 (d, *J* = 7.6 Hz, 2H, 2 × Ar*H*), 2.82 (t, *J* = 2.6 Hz, 1H, CC*H*), 2.71 (t, *J* = 7.4 Hz, 2H, C*H*_2_CH_2_CH_2_CCH), 2.28 (td, *J* = 7.2, 2.6 Hz, 2H, CH_2_CH_2_C*H*_2_CCH), 2.01–1.85 (m, 8H, CH_2_C*H*_2_CH_2_CCH/2(C*H*_3_)); ^**13**^**C NMR (75 MHz, DMSO-*****d***_**6**_**) δ** 187.2 (CO), 159.2, 159.1, 150.3, 137.8, 136.4 (2 × Ar*C*), 133.1, 131.3, 127.8 (2 × Ar*C*), 127.5, 127.3 (2 × Ar*C*), 126.8, 126.4 (2 × Ar*C*), 124.6, 105.7, 83.9, 71.8, 33.0, 25.6, 20.5 (2(*C*H_3_)), 17.4; **HRMS***m***/***z* (ESI^+^) calcd for C_26_H_24_N_3_O_2_ [M + H]^+^ 410.1869.; found, 410.1878.

#### 2-Amino-5-benzoyl-4-(4-morpholinophenyl)-1*H*-pyrrole-3-carboxamide **12**

The desired product was prepared according to the general procedure **A** with the following specific amounts: *N*-(2-oxo-2-phenylethyl)methanesulfonamide **2** (0.250 g, 1.17 mmol, 1 equiv), 4-morpholinobenzaldehyde **9** (0.291 g, 1.52 mmol, 1.3 equiv), cyanoacetamide **4** (0.128 g, 1.52 mmol, 1.3 equiv) and anhydrous granular K_2_CO_3_ (0.097 g, 0.70 mmol, 0.6 equiv) in EtOH 10 mL). Yellow precipitate was collected and washed with DCM/MeOH (1 mL/1 mL). The desired product was also identified in the filtrate. More solid material precipitated from filtrate upon standing. This precipitate was collected and showed a very close, but different *R*_f_ to the initial precipitate. Purification of the filtrate proved to be challenging as many side products were present in crude reaction mixture. The desired product 2-amino-5-benzoyl-4-(3-nitrophenyl)-1*H*-pyrrole-3-carboxamide **12** (0.165 g, 36%) was obtained as a yellow solid from initial precipitate collected. The pyrrole intermediate 2-amino-5-benzoyl-1-(methylsulfonyl)-4-(4-morpholinophenyl)-4,5-dihydro-1*H*-pyrrole-3-carboxamide **12b** was also identified (0.034 g, 6%) as a white solid. Intermediate **12b** was only characterized by ^1^H NMR spectroscopy and HRMS analysis.

***R***_**f**_ = 0.38 (EtOAc/DCM/MeOH, 2.8:2:0.2); ^**1**^**H NMR (300 MHz, DMSO**-***d***_**6**_**) δ** 10.79 (br s, 1H, N*H*), 7.17–7.05 (m, 3H, 3 × Ar*H*), 7.03–6.95 (m, 2H, 2 × Ar*H*), 6.86 (d, *J* = 8.7 Hz, 2H, 2 × Ar*H*), 6.72 (br s, 1H, N*H*_2_), 6.64 (d, *J* = 8.7 Hz, 2H, 2 × Ar*H*), 6.36 (s, 2H, N*H*_2_), 4.86 (br s, 1H, N*H*_2_), 3.73–3.66 (m [app. br t], *J* = 4.8 Hz, 4H, N(CH_2_C*H*_2_)_2_O), 3.04–2.98 (m [app. br t], *J* = 4.8 Hz, 4H, N(C*H*_2_CH_2_)_2_O); ^**13**^**C NMR (75 MHz, DMSO-*d*_6_) δ** 183.7 (*C*O), 167.3, 150.3, 149.4, 139.3, 132.5, 131.0 (2 × Ar*C*), 129.3, 127.7 (2 × Ar*C*), 127.0 (2 × Ar*C*), 124.0, 121.2, 114.5 (2 × Ar*C*), 98.8, 65.9, 48.2; **HRMS***m***/***z* (ESI^+^) calcd for C_22_H_23_N_4_O_3_ [M + H]^+^ 391.1770; found, 391.1767.

#### 2-Amino-5-benzoyl-1-(methylsulfonyl)-4-(4-morpholinophenyl)-4,5-dihydro-1*H*-pyrrole-3-carboxamide **12b**

^**1**^**H NMR (300 MHz, DMSO-*d*_6_) δ** 7.76–7.63 (m, 2H, 2 × Ar*H*), 7.50–7.35 (m, 3H, 3 × Ar*H*), 7.19 (d, *J* = 8.7 Hz, 2H, 2 × Ar*H*), 6.94 (d, J = 8.7 Hz, 2H, 2 × Ar*H*), 4.21 (d, *J* = 4.2 Hz, 1H, C*H*), 3.78–3.69 (m [app. br t], *J* = 4.8 Hz, 4H, N(CH_2_C*H*_2_)_2_O), 3.15–3.07 (m [app. br t], *J* = 4.8 Hz, 4H, N(C*H*_2_CH_2_)_2_O), 2.92 (d, *J* = 2.5 Hz, 1H, C*H*), 2.11 (s, 3H, C*H*_3_). **HRMS***m***/***z* (ESI^+^) calcd for C_23_H_27_N_4_O_5_S [M + H]^+^ 471.1702; found, 471.1698.

#### 6-Benzoyl-5-(4-morpholinophenyl)-2-(pent-4-yn-1-yl)-1,7-dihydro-4*H*-pyrrolo[2,3-*d*]pyrimidin-4-one **15**

The desired product was prepared according to the general procedure **B** with some modifications. The following amounts were utilized: 2-amino-5-benzoyl-4-(4-morpholinophenyl)-1*H*-pyrrole-3-carboxamide **12** (0.050 g, 0.13 mmol, 1 equiv), Na (0.060 g, 2.6 mmol, 20 equiv) from 1.30 mM NaOEt (2 mL, 2.56 mmol) stock solution, and ethyl hex-5-ynoate **6** (0.144 g, 1.02 mmol, 8 equiv). Due to lack of solubility of **12** in reaction conditions (heating to 100 °C), DMSO (0.5 mL) was added. The reaction mixture was stirred at 80 °C for 24 h. Upon completion, reaction mixture was diluted with H_2_O (5 mL) and neutralized using 2 M HCl. Precipitate formation resulted upon neutralization. Precipitate was collected, redissolved in DCM/MeOH (10 mL/2 mL), washed with brine, and dried over MgSO_4_. Drying salts were filtered, and the solvent removed in vacuo. Purification by flash column chromatography (EtOAc/hexane/DCM/MeOH, 2:1.9:1:0.1) afforded 6-benzoyl-5-(4-morpholinophenyl)-2-(pent-4-yn-1-yl)-1,7-dihydro-4*H*-pyrrolo[2,3-*d*]pyrimidin-4-one **15** (0.038 mg, 63%) as a yellow solid.

***R***_**f**_ = 0.60 (EtOAc/DCM/MeOH, 2.8:2:0.2); **MP** decomposes at ≥250 °C; ^**1**^**H NMR (300 MHz, DMSO**-***d***_**6**_**) δ** 12.47 (br s, 1H, N*H*), 11.87 (br s, 1H, N*H*), 7.41 (d, *J* = 7.6 Hz, 2H, 2 × Ar*H*), 7.36–7.28 (m [app. t], *J* = 7.6 Hz, 1H, Ar*H*), 7.19–7.11 (m [app. t], *J* = 7.6 Hz, 2H, 2 × Ar*H*), 7.04 (d, *J* = 8.3 Hz, 2H, 2 × Ar*H*), 6.59 (d, *J* = 8.3 Hz, 2H, 2 × Ar*H*), 3.73–3.65 (m [app. br t], *J* = 4.8 Hz, 4H, N(CH_2_C*H*_2_)O), 3.03–2.95 m [app. br t], *J* = 4.8 Hz, 4H, N(C*H*_2_CH_2_)O), 2.83 (t, *J* = 2.6 Hz, 1H, CC*H*), 2.71 (t, *J* = 7.4 Hz, 2H, C*H*_2_CH_2_CH_2_CCH), 2.27 (td, *J* = 7.2, 2.6 Hz, 2H, CH_2_CH_2_C*H*_2_CCH), 1.97–1.84 (m [app. p], *J* = 7.2 Hz, 2H, CH_2_C*H*_2_CH_2_CCH); Signal at 1.73 ppm (CH_3_) indicates that some of internalized alkyne is present. ^**13**^**C NMR (75 MHz, DMSO-*d*_6_) δ** 187.6, 159.4, 158.7, 149.8, 149.8, 137.6, 131.9 (2 × Ar*C*), 131.6, 129.0 (2 × Ar*C*), 127.7 (2 × Ar*C*), 127.2, 126.8, 123.1, 113.6 (2 × Ar*C*), 104.4, 83.9, 71.8, 66.0, 48.4, 32.9, 25.7, 17.3; **HRMS***m***/***z* (ESI^+^) calcd for C_28_H_27_N_4_O_3_ [M + H]^+^ 467.2083; found, 467.2082; purity: 84%.

### General Procedure C for α-Bromination (Compounds **17**, **23**, and **28**)

To a 2-neck round-bottom flask, charged with a solution of the desired acetophenone substrate (1 equiv) in dry THF, was added phenyltrimethylammonium tribromide (PTAB, 1.00–1.08 equiv) in one portion. The resulting reaction mixture was stirred at RT (or 30 °C) for 1 h. During this time, the reaction changed from bright yellow to a lighter yellow/clear solution with white precipitate being present. Upon completion, the reaction mixture was added to cold H_2_O and extracted with aliquots of EtOAc. The combined organic extracts were then washed with a saturated solution of brine and dried over MgSO_4_. Once filtered, the solvent was removed in vacuo and column chromatography afforded the desired product along with minor brominated side products.

#### 2-Bromo-1-(*o*-tolyl)ethan-1-one **17**

The desired product was prepared according to the general procedure **C** with the following specific amounts: 2-methylacetophenone **16** (0.515 g, 3.84 mmol, 1 equiv), phenyltrimethylammonium tribromide (PTAB, 1.56 g, 4.15 mmol, 1.08 equiv), and THF (25 mL). Extraction was performed with H_2_O (15 mL) and EtOAc (3 × 10 mL). Column chromatography (EtOAc/hexane, 0.3:9.7) afforded 2-bromo-1-(*o*-tolyl)ethan-1-one **17** (0.509 g, 62%) as a white solid. Some starting material remained unreacted and coeluted with a fraction of the product. This coeluted fraction was not considered for yield calculation. The NMR spectra of 2-bromo-1-(*o*-tolyl)ethan-1-one **17** was compared to that reported in the literature and corresponded well.^13^

***R***_**f**_ = 0.57 (EtOAc/hexane, 5:95);^**1**^**H NMR (300 MHz, CDCl**_**3**_**) δ** 7.67 (dd, *J* = 8.0, 1.4 Hz, 1H, Ar*H*), 7.46–7.40 (m [app. td], *J* = 7.5, 1.4 Hz, 1H, Ar*H*), 7.32–7.27 (m, 2H, 2 × Ar*H*), 4.42 (s, 2H, C*H*_2_), 2.53 (s, 3H, C*H*_3_).

#### *N*-[2-Oxo-2-(*o*-tolyl)ethyl]methanesulfonamide **19**

A 2-neck round-bottom flask was charged with hexamethylenetetramine (0.253, 1.81 mmol, 1.1 equiv) in chlorobenzene (4 mL) and stirred at 30 °C. The solution was then treated with the dropwise addition of 2-bromo-1-(*o*-tolyl)ethan-1-one **17** (0.350 g, 1.64 mmol, 1 equiv) dissolved in chlorobenzene (4 mL). Precipitate formation was observed on addition of **17**. The resulting reaction mixture was continued to stir at 30 °C overnight. The precipitate was then collected, washed with chlorobenzene (5 mL), hexane (5 mL), and dried on the high vacuum for 5 h. The solid was then redissolved in EtOH (5 mL) and conc. HCl (0.5 mL). The reaction mixture was stirred at RT for 36 h. Upon completion, the reaction solution was filtered, reduced in vacuo, and lyophilized. The resulting yellow solid was used without further purification for the next reaction step.

To a 2-neck round-bottom flask were added the 2-methyl aminoacetophenone hydrochloride **18** crude product (0.345 g, 1.86 mmol, 1 equiv) and a mixture of acetone/H_2_O (10 mL/1.5 mL). Methanesulfonyl chloride (0.22 mL, 2.9 mmol, 1.5 equiv) was then added in one portion, and the reaction mixture was subsequently cooled to 0 °C, followed by the slow dropwise addition of Et_3_N (0.65 mL, 4.7 mmol, 2.5 equiv). The resulting solution was removed from the ice bath, and an additional volume of acetone (10 mL) was added. The reaction mixture was stirred at RT for 2 h during which time the solution changed in color from orange to yellow. Upon completion, the acetone was removed under reduced pressure. The resulting slurry was then partitioned between EtOAc (20 mL) and saturated NH_4_Cl (15 mL). The organic layer was collected and washed with NaHCO_3_ (25 mL), brine, and dried over MgSO_4_. The drying salts were filtered, and the solvent removed in vacuo. Purification was achieved by column chromatography (EtOAc/hexane, 2:3) affording the desired product *N*-[2-oxo-2-(*o*-tolyl)ethyl]methanesulfonamide as a white solid **19** (0.221 g, 59% (over three steps)).

***R***_**f**_ = 0.73 (EtOAc/Hexane, 3:2);^**1**^**H NMR (600 MHz, CDCl**_**3**_**) δ** 7.69 (dd, *J* = 8.1, 1.3 Hz, 1H, Ar*H*), 7.49–7.45 (m [app. td], *J* = 7.5, 1.3 Hz, 1H, Ar*H*), 7.33–7.29 (m [app. t], *J* = 7.5 Hz, 2H, 2 × Ar*H*), 5.40 (t, *J* = 4.9 Hz, 1H, N*H*), 4.57 (d, *J* = 4.9 Hz, 2H, C*H*_2_), 3.01 (s, 3H, C*H*_3_), 2.56 (s, 3H, C*H*_3_); ^**13**^**C NMR (151 MHz, CDCl**_**3**_**) δ** 196.0, 140.0, 133.6, 133.1, 132.8, 128.8, 126.3, 50.7, 41.0, 21.9; **HRMS***m***/***z* (ESI^+^) calcd for C_10_H_14_NO_3_S [M + H]^+^ 228.0694; found, 228.0685.

#### 2-Amino-5-(2-methylbenzoyl)-4-phenyl-1*H*-pyrrole-3-carboxamide **20**

The desired product was prepared according to the general procedure **A** with the following specific amounts: *N*-[2-oxo-2-(*o*-tolyl)ethyl]methanesulfonamide **19** (0.150 g, 0.660 mmol, 1 equiv), benzaldehyde **3** (0.091 g, 0.86 mmol, 1.3 equiv), cyanoacetamide **4** (0.072 g, 0.86 mmol, 1.3 equiv), and anhydrous granular K_2_CO_3_ (0.054 g, 0.40 mmol, 0.6 equiv) in EtOH (8 mL). The precipitate was collected and washed with DCM/MeOH. The filtrate was reduced in vacuo and purified by flash column chromatography (EtOAc/hexane/DCM/MeOH, 1.4:1.5:2:0.1). The combined precipitate and purified filtrate afforded (0.122 g, 55%) of 2-amino-5-(2-methylbenzoyl)-4-phenyl-1*H*-pyrrole-3-carboxamide **20** as an off-white solid.

***R***_**f**_ = 0.45 (EtOAc/DCM/MeOH, 2.9:2:0.1); ^**1**^**H NMR (300 MHz, DMSO-*****d***_**6**_**) δ** 10.89 (br s, 1H, N*H*), 7.09–6.95 (m, 5H, 5 × Ar*H*), 6.94–6.87 (m [app.td], *J* = 7.4, 1.6 Hz, 1H, Ar*H*), 6.85–6.76 (m [app.t], J = 7.7 Hz, 2H, 2 × Ar*H*), 6.74–6.66 (m [app.t], *J* = 7.4 Hz, 1H, Ar*H*), 6.42 (s, 2H, N*H*_2_), 4.44 (br s, 1H, N*H*), 2.09 (s, 3H, C*H*_3_). Other NH masked in region 6.84–6.60; ^**13**^**C NMR (75 MHz, DMSO-*d*_6_) δ** 184.5 (*C*O), 167.1, 149.6, 139.8, 133.9, 133.5, 132.8, 129.5 (2 × Ar*C*), 129.2, 128.0, 127.6 (2 × Ar*C*), 127.4, 127.1, 124.4, 121.6, 99.3, 18.84 (*C*H_3_); **HRMS***m***/***z* (ESI^+^) calcd for C_19_H_18_N_3_O_2_ [M + H]^+^ 320.1397; found, 320.1399.

#### 6-(2-Methylbenzoyl)-2-(pent-4-yn-1-yl)-5-phenyl-1,7-dihydro-4*H*-pyrrolo[2,3-*d*]pyrimidin-4-one **21**

The desired product was prepared according to the general procedure **B** with some modifications. The following amounts were utilized: 2-amino-5-(2-methylbenzoyl)-4-phenyl-1*H*-pyrrole-3-carboxamide **20** (0.070 g, 0.22 mmol, 1 equiv), Na (0.101 g, 4.38 mmol, 20 equiv) from 1.30 mM NaOEt (3.4 mL, 4.38 mmol) stock solution, and ethyl hex-5-ynoate **6** (0.245 g, 1.75 mmol, 8 equiv). The reaction mixture was stirred at 80 °C for 48 h. Upon completion, reaction mixture was diluted with H_2_O (10 mL) and neutralized using 2 M HCl. Precipitate formation resulted upon neutralization. Precipitate was collected and washed with hexane (10 mL). The filtrate showed the presence of unreacted starting material **20**. Purification of precipitate by flash column chromatography (EtOAc/hexane/DCM/MeOH, 2:2:0.9:0.1) afforded 6-(2-methylbenzoyl)-2-(pent-4-yn-1-yl)-5-phenyl-1,7-dihydro-4*H*-pyrrolo[2,3-*d*]pyrimidin-4-one **21** (0.062 mg, 71%) as an off-white solid.

***R***_**f**_ = 0.77 (EtOAc/DCM/MeOH, 2.8:2:0.2); **MP** 242–248 °C; ^**1**^**H NMR (300 MHz, DMSO-*****d***_**6**_**) δ** 12.59 (br s, 1H, N*H*), 11.89 (br s, 1H, N*H*), 7.08–7.01 (m, 3H, 3 × Ar*H*), 7.00–6.91 (m, 5H, 5 × Ar*H*), 6.81–6.74 (m [app.t], J = 7.8 Hz, 1H, Ar*H*), 2.83 (t, *J* = 2.6 Hz, 1H, CC*H*), 2.70 (t, *J* = 7.4 Hz, 2H, C*H*_2_CH_2_CH_2_CCH), 2.27 (td, *J* = 7.2, 2.6 Hz, 2H, CH_2_CH_2_C*H*_2_CCH), 2.22 (s, 3H, C*H*_3_), 1.97–1.85 (m [app. p], *J* = 7.2 Hz, 2H, CH_2_C*H*_2_CH_2_CCH); ^**13**^**C NMR (75 MHz, DMSO-*****d***_**6**_**) δ** 188.7 (*C*O), 159.2, 150.0, 138.5, 135.6, 132.2, 130.5 (2 × Ar*C*), 130.0, 129.7, 128.5, 128.13, 128.1, 126.5, 126.4 (2 × Ar*C*), 124.7, 105.1, 88.7, 83.8, 71.8, 33.0, 25.7, 19.2, 17.3; **HRMS***m***/***z* (ESI^+^) calcd for C_25_H_22_N_3_O_2_ [M + H]^+^ 396.1712; found, 396.1710.

#### 2-Bromo-1-(4-morpholinophenyl)ethan-1-one **23**

The desired product was prepared according to the general procedure **C** with the following specific amounts: 4-morpholinoacetophenone **22** (0.878 g, 4.28 mmol, 1 equiv), phenyltrimethylammonium tribromide (PTAB, 1.74 g, 4.62 mmol, 1.08 equiv), and THF (50 mL). The reaction mixture was stirred at 30 °C for 1 h. Extraction was performed with H_2_O (25 mL) and EtOAc (3 × 20 mL). Column chromatography (EtOAc/hexane, 2:8 to 4:6) afforded 2-bromo-1-(4-morpholinophenyl)ethan-1-one **23** (0.894 g, 74%) as a yellow solid. The NMR spectra of 2-bromo-1-(4-morpholinophenyl)ethan-1-one **23** was compared to that reported in the literature and corresponded well.^14^

***R***_**f**_ = 0.45 (EtOAc/Hexane, 2:3); ^**1**^**H NMR (300 MHz, CDCl**_**3**_**) δ** 7.91 (d, *J* = 9.0 Hz, 2H, 2 × Ar*H*), 6.88 (d, *J* = 9.0 Hz, 2H, 2 × Ar*H*), 4.36 (s, 2H, C*H*_2_), 3.89–3.82 (m [app. br t], *J* = 4.9 Hz, 4H, N(CH_2_C*H*_2_)_2_O), 3.38–3.30 (m [app. br t], *J* = 4.9 Hz, 4H, N(C*H*_2_CH_2_)_2_O); ^**13**^**C NMR (75 MHz, CDCl**_**3**_**) δ** 189.7, 154.7, 131.3, 124.48, 113.3, 66.6, 47.3, 30.8. Purity: 92.7%

#### 1-(3-Bromo-4-morpholinophenyl)ethan-1-one **28**

Following the general procedure **C** for α-bromination, 1-(3-bromo-4-morpholinophenyl)-ethan-1-one **28** was the favored product when the reaction was performed at 15 °C or lower temperatures.

Additionally, when 4-morpholinoacetophenone **22** (0.050 g, 0.24 mmol, 1 equiv) was treated with *p*-TsOH·H_2_O (0.010 g, 0.049 mmol, 0.2 equiv) and NBS (0.052 g, 0.29 mmol, 1.2 equiv) in chloroform (3 mL) at RT for 1 h, 1-(3-bromo-4-morpholinophenyl)ethan-1-one **28** was obtained in a quantitative yield as a pink oily solid. Identification of product **28** was confirmed by ^1^H NMR spectroscopy only.

***R***_**f**_ = 0.53 (EtOAc/Hexane, 2:3); ^**1**^**H NMR (300 MHz, CDCl**_**3**_**) δ** 8.15 (d, *J* = 2.1 Hz, 1H, Ar*H*), 7.86 (dd, *J* = 8.4, 2.1 Hz, 1H, Ar*H*), 7.03 (d, *J* = 8.4 Hz, 1H, Ar*H*), 3.93–3.82 (m [app. br t], *J* = 4.5 Hz, 4H, N(CH_2_C*H*_2_)_2_O), 3.18–3.07 (m [app. br t], *J* = 4.5 Hz, 4H, N(C*H*_2_CH_2_)_2_O), 2.54 (s, 3H, C*H*_3_).

#### 2-Bromo-1-(3-bromo-4-morpholinophenyl)ethan-1-one **29**

Following the general procedure **C** for α-bromination, 2-bromo-1-(3-bromo-4-morpholinophenyl)ethan-1-one **29** was obtained as a minor side-product when the reaction was performed at RT for 1 h.

Additionally, when 1-(3-bromo-4-morpholinophenyl)ethan-1-one **28** (0.050 g, 0.18 mmol, 1 equiv) was treated with phenyltrimethylammonium tribromide (PTAB, 0.066 g, 0.18 mmol, 1 equiv) and THF (5 mL) at 30 °C for 30 min, 2-bromo-1-(3-bromo-4-morpholinophenyl)ethan-1-one **29** (0.048 g, 75%) was obtained as a pink viscous oil. Identification of product **29** was confirmed by ^1^H NMR spectroscopy only.

***R***_**f**_ = 0.59 (EtOAc/Hexane, 2:3); ^**1**^**H NMR (300 MHz, CDCl**_**3**_**) δ** 8.18 (d, *J* = 2.1 Hz, 1H, Ar*H*), 7.90 (dd, *J* = 8.4, 2.1 Hz, 1H, Ar*H*), 7.05 (d, *J* = 8.4 Hz, 1H, Ar*H*), 4.36 (s, 2H, C*H*_2_), 3.92–3.85 (m [app. br t], *J* = 4.5 Hz, 4H, N(CH_2_C*H*_2_)_2_O), 3.21–3.13 (m [app. br t], *J* = 4.5 Hz, 4H, N(C*H*_2_CH_2_)_2_O).

#### *N*-[2-(4-Morpholinophenyl)-2-oxoethyl]methanesulfonamide **25**

A 2-neck round-bottom flask was charged with hexamethylenetetramine (0.258, 1.84 mmol, 1.1 equiv) in chlorobenzene (6 mL) and stirred at 30 °C. The solution was then treated with the dropwise addition of 2-bromo-1-(4-morpholinophenyl)ethan-1-one **23** (0.475 g, 1.67 mmol, 1 equiv) dissolved in chlorobenzene (6 mL). Precipitate formation was observed on addition of **23**. The resulting reaction mixture was continued to stir at 30 °C for 5 h. The precipitate was then collected, washed with chlorobenzene (5 mL), and redissolved in EtOH (10 mL) and conc. HCl (1 mL). The reaction mixture was stirred at RT for 36 h. Upon completion, the resulting yellow solution having some precipitate present was reduced in vacuo. The water-soluble crude material was then lyophilized affording (0.710 g) of yellow solid. This solid included the desired product **24**, as well as NH_4_Cl byproduct from the hexamethylenetetramine cleavage reaction. Since purification at this point was not possible, the crude product was used for the next reaction step.

To a 2-neck round-bottom flask were added the 4-morpholino-aminoacetophenone hydrochloride **24** crude product (0.710 g, 2.75 mmol, 1 equiv) and a mixture of acetone/H_2_O (15 mL/3 mL). Methanesulfonyl chloride (0.53 mL, 6.9 mmol, 2.5 equiv) was then added in one portion, and the reaction mixture was subsequently cooled to 0 °C. This was followed by the slow dropwise addition of Et_3_N (2.10 mL, 15.1 mmol, 5.5 equiv). The resulting solution was removed from the ice bath, and an additional volume of acetone (20 mL) was added. The reaction mixture was stirred at RT for 5 h. Upon complete consumption of starting material, the acetone was removed under reduced pressure. The resulting slurry was then partitioned between EtOAc (25 mL) and saturated NH_4_Cl (25 mL). The organic layer was collected and washed with NaHCO_3_ (30 mL), brine, and dried over MgSO_4_. The drying salts were filtered, and the solvent removed in vacuo. Purification was achieved by column chromatography (EtOAc/hexane, 3:2 to 4:1) affording the desired product *N*-[2-(4-morpholinophenyl)-2-oxoethyl]methanesulfonamide **25** as a white solid (0.248 g, 50% (over three steps)).

***R***_**f**_ = 0.32 (EtOAc/hexane, 3:2); ^**1**^**H NMR (300 MHz, DMSO-*****d***_**6**_**) δ** 7.86 (d, *J* = 9.0 Hz, 2H, 2 × Ar*H*), 7.23 (t, *J* = 5.7 Hz, 1H, N*H*), 7.00 (d, *J* = 9.0 Hz, 2H, 2 × Ar*H*), 4.50 (d, *J* = 5.7 Hz, 2H, C*H*_2_), 3.77–3.70 (m [app. dd], *J* = 6.0, 3.9 Hz, 4H, N(CH_2_C*H*_2_)_2_O), 3.38–3.26 (m, 7H, N(C*H*_2_CH_2_)_2_O & C*H*_3_); ^**13**^**C NMR (75 MHz, DMSO-*****d***_*6*_**) δ** 192.7 (*C*O), 154.8, 130.3 (2 × Ar*C*), 124.8, 113.4 (2 × Ar*C*), 66.3, 49.0, 47.2, 40.8; **HRMS***m***/***z* (ESI^+^) calcd for C_13_H_19_N_2_O_4_S [M + H]^+^ 299.1066; found, 299.1069.

#### 2-Amino-5-(4-morpholinobenzoyl)-4-phenyl-1*H*-pyrrole-3-carboxamide **26**

The desired product was prepared according to the general procedure **A** with the following specific amounts: *N*-[2-(4-morpholinophenyl)-2-oxoethyl]methanesulfon-amide **25** (0.440 g, 1.48 mmol, 1 equiv), benzaldehyde **3** (0.203 g, 1.92 mmol, 1.3 equiv), cyanoacetamide **4** (0.161 g, 1.92 mmol, 1.3 equiv), and anhydrous granular K_2_CO_3_ (0.102 g, 0.738 mmol, 0.5 equiv) in EtOH (20 mL). The yellow precipitate collected and washed with DCM/MeOH (3 mL/3 mL). The filtrate solvent reduced in vacuo and purified by flash column chromatography (EtOAc/DCM/MeOH, 2.9:2:0.1). The desired product **26** (0.263 g, 46%) was obtained as a yellow solid from precipitate and filtrate purification.

***R***_**f**_ = 0.35 (EtOAc/DCM/MeOH, 2.9:2.0:0.1); ^**1**^**H NMR (300 MHz, DMSO-*****d***_**6**_**) δ** 10.76 (br s, 1H, N*H*), 7.22–7.05 (m, 7H, 7 × Ar*H*), 6.66 (br s, 1H, NH_2_), 6.53 (d, *J* = 8.8 Hz, 2H, 2 × Ar*H*), 6.29 (s, 2H, N*H*_2_), 4.67 (br s, 1H, N*H*_2_), 3.73–3.62 (m [app. br t], *J* = 4.9 Hz, 4H, N(CH_2_C*H*_2_)_2_O), 3.10–2.99 (m [app. br t], *J* = 4.9 Hz, 4H, N(C*H*_2_CH_2_)_2_O); ^**13**^**C NMR (75 MHz, DMSO-*****d***_**6**_**) δ** 183.0, 167.3, 152.1, 148.7, 134.7, 130.5 (2 × Ar*C*), 130.4, 129.7 (2 × Ar*C*), 129.0, 128.0 (2 × Ar*C*), 127.4, 121.1, 112.9 (2 × Ar*C*), 98.3, 65.8, 47.6; **HRMS***m***/***z* (ESI^+^) calcd for C_22_H_23_N_4_O_3_ [M + H]^+^ 391.1770; found, 391.1758.

#### 6-(4-Morpholinobenzoyl)-2-(pent-3-yn-1-yl)-5-phenyl-1,7-dihydro-4*H*-pyrrolo[2,3-*d*]pyrimidin-4-one **30**

The desired product was prepared according to the general procedure **B** with some modifications. The following amounts were utilized: 2-amino-5-(4-morpholinobenzoyl)-4-phenyl-1*H*-pyrrole-3-carboxamide **26** (0.050 g, 0.13 mmol, 1 equiv), Na (0.060 g, 2.6 mmol, 20 equiv) from 1.30 mM NaOEt (2 mL, 2.56 mmol) stock solution, and ethyl hex-5-ynoate **6** (0.144 g, 1.02 mmol, 8 equiv). Due to lack of solubility of **26** in reaction conditions (heating to 100 °C), DMSO (1 mL) was added. The reaction mixture was stirred at 100 °C for 12 h. Upon completion, the reaction mixture was diluted with H_2_O (4 mL) and neutralized using 2 M HCl. Precipitate formation resulted upon neutralization. The precipitate was collected, redissolved in DCM/MeOH (10 mL/2 mL), washed with brine, and dried over MgSO_4_. Drying salts were filtered, and the solvent was removed in vacuo. Purification by flash column chromatography (EtOAc/hexane/DCM/MeOH, 1:1:0.4:0.1) afforded 6-(4-morpholinobenzoyl)-2-(pent-3-yn-1-yl)-5-phenyl-1,7-dihydro-4*H*-pyrrolo[2,3-*d*]pyrimidin-4-one **30** (0.050 mg, 83%) as a yellow solid.

***R***_**f**_ = 0.50 (EtOAc/Hexane/DCM/MeOH, 2:2:0.9:0.1); ^**1**^**H NMR (600 MHz, DMSO-*****d***_**6**_**) δ** 12.48 (br s, 1H, N*H*), 11.87 (br s, 1H, N*H*), 7.43 (d, *J* = 8.7 Hz, 2H, 2 × Ar*H*), 7.26 (d, *J* = 6.5 Hz, 2H, 2 × Ar*H*), 7.15–7.07 (m, 3H, 3 × Ar*H*), 6.69 (d, *J* = 8.7 Hz, 2H, 2 × Ar*H*), 3.67 (t, *J* = 4.8 Hz, 4H, N(CH_2_C*H*_2_)_2_O), 3.17 (t, *J* = 4.8 Hz, 4H, N(C*H*_2_CH_2_)_2_O), 2.79 (t, *J* = 7.4 Hz, 2H, C*H*_2_CH_2_CCCH_3_), 2.62–2.56 (m, 2H, CH_2_C*H*_2_CCCH_3_), 1.72 (t, *J* = 4.8 Hz, 3H, C*H*_3_); ^**13**^**C NMR (151 MHz, DMSO) δ** 186.0 (*C*O), 159.3, 157.0, 153.5, 149.2, 132.9, 130.9, 127.9, 127.0, 126.7, 126.4, 123.8, 103.9, 78.0, 76.6, 65.7, 46.8, 46.8, 33.5, 16.2, 3.2; **HRMS***m***/***z* (ESI^+^) calcd for C_28_H_27_N_4_O_3_ [M + H]^+^ 467.2083; found, 467.2078; purity: 99%

##### Crystal Data for **30**

@C_28.75_H_27.50_Cl_1.50_N_4_O_3.15_, *M* = 532.62, yellow shard, 0.165 × 0.106 × 0.066 mm^3^, triclinic, space group *P*-1 (no. 2), *a* = 8.8745(18), *b* = 10.836(2), *c* = 15.019(3) Å, *a* = 109.382(3), *b* = 102.360(4), γ = 95.031(4)°, *V* = 1311.0(5) Å^3^, *Z* = 2, *D*_c_ = 1.349 g cm^–3^, *F*_000_ = 557, Bruker APEX II DUO CCD, Mo Kα radiation, *l* = 0.71073 Å, *T* = 100(2) K, 2*q*_max_ = 54.3°, 29105 reflections collected, 5803 unique (*R*_int_ = 0.0510). Final *GooF* = 1.094, *R*1 = 0.0841, *wR*2 = 0.2388, *R* indices based on 4867 reflections with *I* > 2s(I) (refinement on *F*^2^), 362 parameters, 0 restraints. Lp and absorption corrections applied, *m* = 0.236 mm^–1^. Data submitted to CCDC as submission number: 2178967.

#### 6-Benzoyl-2-(pent-3-yn-1-yl)-5-phenyl-1,7-dihydro-4*H*-pyrrolo[2,3-*d*]pyrimidin-4-one **31**

The desired product was prepared according to the general procedure **A** with some modifications. The following amounts were utilized: 2-amino-5-benzoyl-4-phenyl-1*H*-pyrrole-3-carboxamide **5** (0.150 g, 0.491 mmol, 1 equiv), commercially available NaOEt (21% in EtOH) (2.4 mL, 15 equiv), and ethyl hex-5-ynoate **6** (0.551 g, 3.93 mmol, 8 equiv). DMSO (2 mL) was then added to the reaction mixture, which was stirred at 90 °C for 12 h. Upon completion, the reaction mixture was diluted with H_2_O (5 mL) and neutralized using 2 M HCl. Precipitate formation resulted upon neutralization. The precipitate was collected, redissolved in DCM/MeOH (10 mL/2 mL), washed with brine, and dried over MgSO_4_. Drying salts were filtered, and the solvent removed in vacuo. Purification by flash column chromatography (EtOAc/hexane/DCM/MeOH, 1:1:0.4:0.1) afforded 6-benzoyl-2-(pent-3-yn-1-yl)-5-phenyl-1,7-dihydro-4*H*-pyrrolo[2,3-*d*]pyrimidin-4-one **31** (0.126 mg, 67%) as an off-white solid.

***R***_**f**_ = 0.49 (EtOAc/Hexane, 3:2); **MP** decomposes at ≥254 °C; ^**1**^**H NMR (300 MHz, DMSO-*****d***_**6**_**) δ** 12.65 (br s, 1H, N*H*), 11.93 (br s, 1H, N*H*), 7.45–7.39 (d, *J* = 8.3 Hz, 2H, 2 × Ar*H*), 7.35–7.28 (m [app. t], *J* = 7.4 Hz, 1H, Ar*H*), 7.20–7.08 (m, 4H, 4 × Ar*H*), 7.06–6.98 (m, 3H, 3 × Ar*H*), 2.80 (t, *J* = 7.4 Hz, 2H, C*H*_2_CH_2_CCCH_3_), 2.65–2.55 (m, 2H, CH_2_C*H*_2_CCCH_3_), 1.72 (t, *J* = 2.5 Hz, 3H, C*H*_3_); ^**13**^**C NMR (75 MHz, DMSO-*****d***_**6**_**) δ** 187.6 (*C*O), 159.3, 157.9, 149.7, 137.5, 132.5, 131.8, 131.1 (2 × Ar*C*), 129.04 (2 × Ar*C*), 127.7 (2 × Ar*C*), 127.3, 126.8 (2 × Ar*C*), 126.7, 126.7, 104.5, 77.9, 76.6, 33.6, 16.2, 3.2. **HRMS***m***/***z* (ESI^+^) calcd for C_24_H_20_N_3_O_2_ (M+H^+^) 382.1556; found, 382.1549.

##### Crystal Data for **31**

C_48_H_38_N_6_O_4_, M = 762.84, colorless plate, 0.664 × 0.069 × 0.056 mm^3^, triclinic, space group *P*-1 (no. 2), *a* = 9.5121(8), *b* = 15.1843(12), *c* = 15.6252(12) Å, *a* = 67.376(3), *b* = 73.177(3), γ = 72.411(3)°, *V* = 1946.9(3) Å^3^, *Z* = 2, *D*_c_ = 1.301 g cm^–3^, *F*_000_ = 800, Bruker APEX DUO CCD, Mo Kα radiation, *l* = 0.71073 Å, *T* = 100(2) K, 2*q*_max_ = 54.4°, 41681 reflections collected, 8639 unique (*R*_int_ = 0.0494). Final *GooF* = 1.027, *R*1 = 0.0477, *wR*2 = 0.1049, *R* indices based on 6227 reflections with *I* > 2s(I) (refinement on *F*^2^), 541 parameters, 0 restraints. Lp and absorption corrections applied, *m* = 0.085 mm^–1^. Data submitted to CCDC as submission number: 2178968.

#### 6-(4-Morpholinobenzoyl)-2-(pent-4-yn-1-yl)-5-phenyl-1,7-dihydro-4*H*-pyrrolo[2,3-*d*]pyrimidin-4-one **27**

The desired product was prepared according to the general procedure **B** with some modifications. The following amounts were utilized: 2-amino-5-(4-morpholinobenzoyl)-4-phenyl-1*H*-pyrrole-3-carboxamide **26** (0.034 g, 0.087 mmol, 1 equiv), Na (0.020 g, 0.87 mmol, 10 equiv) from 0.65 mM NaOEt (1.3 mL, 0.87 mmol) stock solution, and ethyl hex-5-ynoate **6** (0.098 g, 0.70 mmol, 8 equiv). Due to lack of solubility of **26** in reaction conditions (heating to 100 °C), DMSO (2 mL) was added. The reaction mixture was stirred at 55 °C for 58 h. Complete consumption of starting material was not achieved even at longer reaction times. Upon completion, the reaction mixture was diluted with H_2_O (5 mL) and neutralized using 2 M HCl. The mixture was then partitioned between DCM/MeOH (10 mL/2 mL) and H_2_O (5 mL). The organic layer was collected, reduced in vacuo, and lyophilized for 5 h. Purification by flash column chromatography (EtOAc/hexane/DCM/MeOH, 1:1:0.4:0.1) and trituration with diethyl ether afforded a mixture of the internal **30** and external alkyne products **27**. Further purification by PREP TLC (EtOAc/Hexane/DCM/MeOH, 1:1:0.4:0.1) afforded 6-(4-morpholinobenzoyl)-2-(pent-4-yn-1-yl)-5-phenyl-1,7-dihydro-4*H*-pyrrolo[2,3-*d*]pyrimidin-4-one **27** (0.012 mg, 29%) (52% brsm) as a light yellow solid.

***R***_**f**_ = 0.50 (EtOAc/hexane/DCM/MeOH, 2:2:0.9:0.1); ^**1**^**H NMR (300 MHz, DMSO-*****d***_**6**_**) δ:** 12.43 (br s, 1H, N*H*), 11.84 (br s, 1 H, N*H*), 7.44 (d, *J* = 8.9 Hz, 2H, 2 × Ar*H*), 7.30–7.22 (m, 2H, 2 × Ar*H*), 7.17–7.06 (m, 3H, 3 × Ar*H*), 6.70 (d, J = 8.9, 2H, 2 × Ar*H*), 3.67 (t, *J* = 4.9 Hz, 4H, N(CH_2_C*H*_2_)_2_O), 3.17 (t, *J* = 4.9 Hz, 4H, N(C*H*_2_CH_2_)_2_O), 2.83 (t, *J* = 2.6 Hz, 1H, CC*H*), 2.71 (t, *J* = 7.4 Hz, 2H, C*H*_2_CH_2_CH_2_CCH), 2.27 (td, *J* = 7.2, 2.6 Hz, 2H, CH_2_CH_2_C*H*_2_CCH), 1.97–1.85 (m [app. p], *J* = 7.2 Hz, 2H, CH_2_C*H*_2_CH_2_CCH); ^**13**^**C NMR (300 MHz, DMSO-*****d***_**6**_**) δ** 186.5, 159.9, 158.2, 154.0, 149.8, 133.5, 131.9, 131.3, 127.5, 126.8, 124.3, 113.0, 104.4, 84.4, 72.2, 66.2, 47.3, 33.31, 26.17, 17.77. Two quaternary carbons not identified in spectra; **HRMS***m***/***z* (ESI^+^) calcd for C_28_H_27_N_4_O_3_ [M + H]^+^ 467.2083; found, 467.2078; purity: 90.5%

#### 1-[4-(Benzyloxy)phenyl]ethan-1-one **37**

A 2-neck round-bottom flask was charged with 4-hydroxyacetophenone **36** (0.500 g, 3.67 mmol, 1.0 equiv), K_2_CO_3_ (0.761 g, 5.51 mmol, 1.5 equiv), and KI (0.122 g, 0.734 mmol, 0.2 equiv). Acetone (7 mL) was then added followed by the rapid dropwise addition of benzyl bromide (0. 48 mL, 4.0 mmol, 1.1 equiv) diluted in acetone (2 mL). The reaction mixture was stirred overnight at RT under a N_2_ atmosphere. Upon completion, the solution was concentrated under reduced pressure and partitioned between EtOAc (20 mL) and H_2_O (20 mL). The aqueous layer was further extracted with EtOAc (2 × 10 mL). The combined organic layers were washed with brine, dried over MgSO_4_, filtered, and concentrated in vacuo. The yellow solid obtained (0.795 g, 96%) was used for the next reaction step without further purification. The ^1^H NMR spectrum of 1-[4-(benzyloxy)phenyl]ethan-1-one **37** was compared to that reported in the literature and corresponded well.^15^

***R***_**f**_ = 0.37 (EtOAc/hexane, 1:9); ^**1**^**H NMR (300 MHz, CDCl**_**3**_**) δ** 7.94 (d, *J* = 8.9 Hz, 2H, 2 × Ar*H*), 7.47–7.31 (m, 5H, 5 × Ar*H*), 7.01 (d, *J* = 8.9 Hz, 2H, 2 × Ar*H*), 5.14 (s, 2H, C_6_H_5_C*H*_2_), 2.55 (s, 3H, C*H*_3_).

#### 1-[4-(Benzyloxy)phenyl]-2-bromoethan-1-one **38**

The desired product was prepared according to the general procedure **C** with the following specific amounts: 1-[4-(benzyloxy)phenyl]ethan-1-one **37** (0.300 g, 1.33 mmol, 1 equiv), phenyltrimethylammonium tribromide (PTAB, 0.498 g, 1.33 mmol, 1 equiv) and THF (5 mL). Extraction performed with H_2_O (15 mL) and EtOAc (3 × 10 mL). Column chromatography (EtOAc/hexane, 1:9) afforded 1-[4-(benzyloxy)phenyl]-2-bromoethan-1-one **38** (0.318 mg, 79%) as a white solid. The NMR spectra of 1-[4-(benzyloxy)phenyl]-2-bromoethan-1-one **38** was compared to that reported in the literature and corresponded well.^16^

***R***_**f**_ = 0.48 (EtOAc/hexane, 1:9); ^**1**^**H NMR (300 MHz, CDCl**_**3**_**) δ** 7.97 (d, *J* = 8.9 Hz, 2H, 2 × Ar*H*), 7.46–7.30 (m, 5H, 5 × Ar*H*), 7.04 (d, *J* = 8.9 Hz, 2H, 2 × Ar*H*), 5.15 (s, 2H, C_6_H_5_C*H*_2_), 4.39 (s, 2H, C*H*_2_Br). ^**13**^**C NMR (75 MHz, CDCl**_**3**_**) δ** 190.0 (*C*O), 163.4, 136.1, 131.5 (2 × Ar*C*), 128.9 (2 × Ar*C*), 128.5, 127.6 (2 × Ar*C*), 127.3, 115.1 (2 × Ar*C*), 70.4, 30.8.

#### *N*-{2-[4-(Benzyloxy)phenyl]-2-oxoethyl}methanesulfonamide **39**

A 2-neck round-bottom flask was charged with hexamethylenetetramine (0.323 g, 2.31 mmol, 1.1 equiv) in anhydrous chlorobenzene (7 mL) and stirred at 30 °C. To this solution was then added 1-[4-(benzyloxy)phenyl]-2-bromoethan-1-one **38** (0.640 mg, 2.10 mmol, 1 equiv) dissolved in anhydrous chlorobenzene (5 mL) in a dropwise manner. The reaction mixture was stirred at 30 °C for a further 6 h. The formed precipitate was collected, washed with chlorobenzene (5 mL), and dried on the high vacuum pump for 2 h. This hexamethylenetetramine salt of the product was then redissolved in a mixture of EtOH (4 mL)/ concentrated HCl (0.5 mL) and stirred at room temperature under N_2_ for 2 d. Hereafter, the reaction mixture was cooled to 0 °C and the amine salt of the product appearing as a white precipitate was collected. The collected white solid was transferred to a 2-neck round-bottom flask and stirred in a mixture of acetone/H_2_O (12 mL/4 mL). Methanesulfonyl chloride (0.720 mg, 6.29 mmol, 3.0 equiv) was then added in one portion, and the reaction mixture was subsequently cooled in an ice bath, followed by the slow dropwise addition of Et_3_N (1.47 mL, 10.5 mmol, 5 equiv). The resulting solution was removed from the ice bath, and an additional volume of acetone (20 mL) was added. The reaction mixture was stirred overnight at RT after which the acetone was removed under reduced pressure. The resulting slurry was then partitioned between EtOAc (10 mL) and saturated NH_4_Cl (10 mL). The organic layer was separated and washed with NaHCO_3_ (10 mL), brine, and dried over MgSO_4_. The drying salts were filtered, and the solvent was removed in vacuo. Purification was achieved by column chromatography (DCM/MeOH, 98.5:1.5) affording the desired product **39** as a white solid (455 mg, 69% (three steps)). The NMR spectra of *N*-{2-[4-(benzyloxy)phenyl]-2-oxoethyl}methanesulfonamide **39** was compared to that reported in the literature and corresponded well.^16^

***R***_**f**_ = 0.50 (EtOAc/Hexane, 1:1); ^**1**^**H NMR (400 MHz, CDCl**_**3**_**) δ** 7.92 (d, *J* = 8.8 Hz, 2H, 2 × Ar*H*), 7.45–7.33 (m, 5H, 5 × Ar*H*), 7.05 (d, *J* = 8.8 Hz, 2H, 2 × Ar*H*), 5.37 (t, *J* = 4.8 Hz, 1H, N*H*), 5.16 (s, 2H, C_6_H_5_C*H*_2_), 4.61 (d, *J* = 4.8 Hz, 2H, C*H*_2_NH), 2.99 (s, 3H, C*H*_3_). ^**13**^**C NMR (101 MHz, CDCl**_**3**_**) δ** 191.7 (*C*O), 163.8, 135.9, 130.5 (2 × Ar*C*), 128.9 (2 × Ar*C*), 128.53, 127.6 (2 × Ar*C*), 127.1, 115.3 (2 × Ar*C*), 70.5, 48.9, 40.8.

#### *tert*-Butyl {2-[4-(Benzyloxy)phenyl]-2-oxoethyl}(methylsulfonyl)carbamate **40**

A 2-neck round-bottom flask was charged with *N*-{2-[4-(benzyloxy)phenyl]-2-oxoethyl}methanesulfonamide **39** (0.575 g, 1.81 mmol, 1 equiv) and dissolved in dry THF (30 mL). The resulting solution was treated with Boc_2_O (0.473 mg, 2.17 mmol, 1.2 equiv) and a catalytic amount of DMAP (spatula tip). The reaction mixture was then cooled in an ice bath followed by the dropwise addition of Et_3_N (0.28 mL, 2.0 mmol, 1.1 equiv) diluted in THF (2 mL). The reaction was stirred for 5 h and allowed to reach RT with time. Upon complete consumption of the starting material, the solvent was removed under reduced pressure. Purification by column chromatography (EtOAc/hexane, 1:4) afforded *tert*-butyl {2-[4-(benzyloxy)phenyl]-2-oxoethyl}(methylsulfonyl)carbamate **40** (0.656 g, 87%) as a white solid.

***R***_**f**_ = 0.60 (EtOAc/Hexane, 3:7); ^**1**^**H NMR (400 MHz, CDCl**_**3**_**) δ** 7.91 (d, *J* = 8.8 Hz, 2H, 2 × Ar*H*), 7.45–7.32 (m, 5H, 5 × Ar*H*), 7.04 (d, *J* = 8.8 Hz, 2H, 2 × Ar*H*), 5.15 (s, 2H, C*H*_2_), 5.14 (s, 2H, C*H*_2_) 3.50 (s, 3H, C*H*_3_), 1.49 (s, 9H, 3 × C*H*_3_). ^**13**^**C NMR (75 MHz, CDCl**_**3**_**) δ** 192.0 (*C*O), 163.5, 151.4, 136.1, 130.4 (2 × Ar*C*), 128.9 (2 × Ar*C*), 128.5, 127.6 (2 × Ar*C*), 127.6, 115.1 (2 × Ar*C*), 85.1, 70.4, 52.2, 41.4, 28.0 (3 × *C*); **HRMS***m***/***z* (ESI^+^) calcd for C_21_H_26_NO_6_S (M+H^+^) 420.1481; found, 420.1481.

#### *tert*-Butyl [2-(4-Hydroxyphenyl)-2-oxoethyl](methylsulfonyl)carbamate **41**

A three-neck round-bottom flask was charged with Pd/C (0.013 mg, 0.12 mmol, 15 mol %) and evacuated. *tert*-Butyl {2-[4-(benzyloxy)phenyl]-2-oxoethyl}(methylsulfonyl)carbamate **40** (0.338 g, 0.806 mmol, 1 equiv) was dissolved in EtOAc (5 mL) and added to the reaction flask via a syringe while under vacuum. The flask was then equipped with a H_2_ balloon and stirred under a H_2_ atmosphere for 8 h at RT. Upon completion, the reaction mixture was filtered over Celite. The solvent was then removed under reduced pressure and purified via column chromatography (EtOAc/hexane, 2:3) affording the desired product *tert*-butyl [2-(4-hydroxyphenyl)-2-oxoethyl](methylsulfonyl)carbamate **41** (0.210 g, 79%) as a white solid.

***R***_**f**_ = 0.33 (EtOAc/Hexane, 3:7); ^**1**^**H NMR (400 MHz, CDCl**_**3**_**) δ** 7.80 (d, *J* = 8.8 Hz, 2H, 2 × Ar*H*), 6.86 (d, *J* = 8.8 Hz, 2H, 2 × Ar*H*), 6.24 (br s, 1H, O*H*), 5.12 (s, 2H, C*H*_2_), 3.51 (s, 3H, C*H*_3_), 1.49 (s, 9H, 3 × C*H*_3_); ^**13**^**C NMR (101 MHz, CDCl**_**3**_**) δ** 191.9 (*C*O), 161.3, 151.5, 130.7 (2 × Ar*C*), 127.3, 115.9 (2 × Ar*C*), 85.5, 52.2, 41.7, 28.0 (3 × *C*); **HRMS***m***/***z* (ESI^+^) calcd for C_14_H_20_NO_6_S (M+H^+^) 330.1011; found, 330.1013.

#### 2-Ethoxyethyl 4-Methylbenzenesulfonate **43**

To a two-neck round-bottom flask was added *p*-toluenesulfonyl chloride (2.33 g, 12.2 mmol, 1.1 equiv) and DCM (10 mL). The resulting solution was cooled to 0 °C and then treated with 2-ethoxyethanol **42** (1.00 g, 11.1 mmol, 1 equiv), followed by the dropwise addition of Et_3_N (1.36 g, 13.3 mmol, 1.2 equiv) diluted in DCM (2 mL). The reaction mixture was stirred overnight and allowed to warm to RT with time. Precipitate formation was observed. The reaction mixture was diluted with DCM (15 mL) and partitioned with H_2_O (20 mL). The organic layer was collected, washed with NaHCO_3_ (15 mL), brine, and dried over MgSO. The drying salts were filtered, and the solvent removed in vacuo. Purification via column chromatography ((EtOAc/hexane, 1:4) afforded 2-ethoxyethyl 4-methylbenzenesulfonate **43** (2.21 g, 81%) as a clear oil. The NMR spectra of **43** was compared to that reported in the literature and corresponded well.

^**1**^**H NMR (300 MHz, DMSO-*****d***_**6**_**) δ** 7.78 (d, *J* = 8.4 Hz, 2H, 2 × Ar*H*), 7.48 (d, *J* = 8.4 Hz, 2H, 2 × Ar*H*), 4.13–4.08 (m, 2H, C*H*_2_), 3.54–3.49 (m, 2H, CH_2_), 3.35 (q, *J* = 7.0 Hz, 2H, C*H*_2_CH_3_), 2.42 (s, 3H, C*H*_3_), 1.03 (t, *J* = 7.0 Hz, 3H, CH_2_C*H*_3_).

### General Procedure D for Coupling of Solubilizing Groups (Compounds **44–46**)

A two-neck round-bottom flask was charged with a solution of *tert*-butyl [2-(4-hydroxyphenyl)-2-oxoethyl](methylsulfonyl)carbamate **41** (1 equiv) in acetone. The resulting solution was then treated with the specific solubilizing group (1.5/2.5 equiv), K_2_CO_3_ (2.0/3.5 equiv), and a catalytic amount of KI. The reaction mixture was heated under reflux for 24 h until complete consumption of starting material. Solvent was removed under reduced pressure, and the reaction mixture was partitioned between DCM and H_2_O. The aqueous layer was further extracted with DCM after which the organic layers were combined, washed with brine, and dried over MgSO_4_. The drying salts were filtered, and the solvent removed in vacuo. Purification via column chromatography afforded the desired products **44**, **45**, and **46**.

#### *tert*-Butyl {2-[4-(2-Ethoxyethoxy)phenyl]-2-oxoethyl}(methylsulfonyl)carbamate **44**

The desired product was prepared according to the general procedure **D** with the following specific amounts: *tert*-butyl [2-(4-hydroxyphenyl)-2-oxoethyl](methylsulfonyl)-carbamate **41** (0.250 g, 0.759 mmol, 1 equiv), 2-ethoxyethyl 4-methylbenzenesulfonate **43** (0.278 g, 1.14 mmol, 1.5 equiv), K_2_CO_3_ (0.210 g, 1.52 mmol, 2 equiv), and KI (spatula tip) in acetone (25 mL). Extraction was performed with H_2_O (25 mL) and DCM (2 × 25 mL). Column chromatography (EtOAc/hexane, 3:7) afforded *tert*-butyl {2-[4-(2-ethoxyethoxy)phenyl]-2-oxoethyl}(methylsulfonyl)carbamate **44** (0.196 g, 64%) as a colorless viscous oil.

***R***_**f**_ = 0.45 (EtOAc/Hexane, 3:7); ^**1**^**H NMR (300 MHz, CDCl**_**3**_**) δ** 7.89 (d, *J* = 9.0 Hz, 2H, 2 × Ar*H*), 6.98 (d, *J* = 9.0 Hz, 2H, 2 × Ar*H*), 5.13 (s, 2H, CH_2_), 4.19 (dd, *J* = 5.5, 4.1 Hz, 2H, OC*H*_2_CH_2_OCH_2_CH_3_), 3.81 (dd, *J* = 5.5, 4.1 Hz, 2H, OCH_2_C*H*_2_OCH_2_CH_3_), 3.60 (q, *J* = 6.9 Hz, 2H, C*H*_2_CH_3_) 3.49 (s, 3H, CH_3_), 1.48 (s, 9H, 3 × C*H*_3_), 1.24 (t, *J* = 6.9, 3H, CH_2_C*H*_3_); ^**13**^**C NMR (75 MHz, CDCl**_**3**_**) δ** 192.0 (*C*O), 163.7, 151.4, 130.4 (2 × Ar*C*), 127.5, 114.9 (2 × Ar*C*), 85.0, 68.8, 67.9, 67.1, 52.2, 41.5, 28.0 (3 × *C*H_3_), 15.3; **HRMS***m***/***z* (ESI^+^) calcd for C_18_H_28_NO_7_S (M+H^+^) 402.1586; found, 402.1590.

#### *tert*-Butyl (Methylsulfonyl){2-[4-(2-morpholinoethoxy)phenyl]-2-oxoethyl}carbamate **45**

The desired product was prepared according to the general procedure **D** with the following specific amounts: *tert*-butyl [2-(4-hydroxyphenyl)-2-oxoethyl](methylsulfonyl)-carbamate **41** (0.200 g, 0.607 mmol, 1 equiv), 2-morpholinoethyl chloride hydrochloride (0.282 g, 1.52 mmol, 2.5 equiv), K_2_CO_3_ (0.294 g, 2.13 mmol, 3.5 equiv), and KI (spatula tip) in acetone (15 mL). Extraction was performed with H_2_O (20 mL) and DCM (2 × 20 mL). Column chromatography (EtOAc/hexane/DCM/MeOH, 2:2:0.9:0.1) afforded *tert*-butyl (methylsulfonyl){2-[4-(2-morpholinoethoxy)phenyl]-2-oxoethyl}carbamate **45** (0.229 g, 85%) as a yellow viscous oil.

***R***_**f**_ = 0.32 (EtOAc/Hexane/DCM/MeOH, 2:2:0.9:0.1); ^**1**^**H NMR (300 MHz, CDCl**_**3**_**) δ** 7.90 (d, *J* = 8.9, 2H, 2 × Ar*H*), 6.96 (d, *J* = 8.9, 2H, 2 × Ar*H*), 5.14 (s, 2H, C*H*_2_), 4.18 (t, *J* = 5.7 Hz, 2H, NCH_2_C*H*_2_O), 3.78–3.69 (m, 4H, N(CH_2_C*H*_2_)_2_O), 3.49 (s, 3H, C*H*_3_), 2.83 (t, *J* = 5.7 Hz, 2H, NC*H*_2_CH_2_O), 2.64–2.53 (m, 4H, N(C*H*_2_CH_2_)_2_O), 1.48 (s, 9H, 3 × C*H*_3_); ^**13**^**C NMR (75 MHz, CDCl**_**3**_**) δ** 192.0 (*C*O), 163.5, 151.4, 130.4 (2 × Ar*C*), 127.6, 114.8 (2 × Ar*C*), 85.1, 67.0, 66.3, 57.5, 54.3, 52.2, 41.5, 28.0 (3 × *C*); **HRMS***m***/***z* (ESI^+^) calcd for C_20_H_31_N_2_O_7_S (M+H^+^) 443.1852; found, 443.1861.

#### *tert*-Butyl (Methylsulfonyl)(2-oxo-2-{4-[2-(pyrrolidin-1-yl)ethoxy]phenyl}ethyl)carbamate **46**

The desired product was prepared according to the general procedure **D** with the following specific amounts: *tert*-butyl [2-(4-hydroxyphenyl)-2-oxoethyl](methylsulfonyl)carbamate **41** (0.260 g, 0.789 mmol, 1 equiv), 2-pyrrolidinoethyl hydrochloride (0.336 g, 1.97 mmol, 2.5 equiv), K_2_CO_3_ (0.382 g, 2.76 mmol, 3.5 equiv), and KI (spatula tip) in acetone (20 mL). Extraction was performed with H_2_O (25 mL) and DCM (2 × 25 mL). Column chromatography (DCM/MeOH, 9:1) afforded *tert*-butyl (methylsulfonyl)(2-oxo-2-{4-[2-(pyrrolidin-1-yl)ethoxy]phenyl}ethyl)carbamate **46** (0.260 g, 77%) as a colorless viscous oil.

***R***_**f**_ = 0.43 (DCM/MeOH, 9:1); ^**1**^**H NMR (300 MHz, CDCl**_**3**_**) δ** 7.90 (d, *J* = 8.9 Hz, 2H, 2 × Ar*H*), 6.98 (d, *J* = 8.9 Hz, 2H, 2 × Ar*H*), 5.13 (s, 2H, CH_2_), 4.30 (t, *J* = 5.7 Hz, 2H, NCH_2_C*H*_2_O), 3.49 (s, 3H, CH_3_), 3.09 (t, *J* = 5.7 Hz, 2H, NC*H*_2_CH_2_O), 2.88–2.78 (m, 4H, N(C*H*_2_CH_2_)_2_), 1.98–1.86 (m, 4H, N(CH_2_C*H*_2_)_2_), 1.48 (s, 9H, 3 × C*H*_3_); ^**13**^**C NMR (75 MHz, CDCl**_**3**_**) δ** 192.0 (*C*O), 163.2, 151.3, 130.5 (2 × Ar*C*), 127.7, 114.8 (2 × Ar*C*), 85.1, 66.6, 54.9, 54.7, 52.2, 41.5, 28.0 (3 × *C*H_3_), 23.6.

#### General Procedure E for *tert*-Butoxycarbonyl (Boc) Deprotection (Compounds **47–49**)

To a 1-neck round-bottom flask was added a solution of *tert*-butoxycarbonyl protected compound (**44**, **45**, or **46**) in DCM. The reaction solution was cooled to 0 °C and treated with the dropwise addition of TFA. The resulting reaction mixture was then stirred at RT for 3–4 h. Upon completion, DCM and TFA were removed under reduced pressure. Purification via column chromatography afforded the desire products (**47**, **48**, or **49**).

#### *N*-{2-[4-(2-Ethoxyethoxy)phenyl]-2-oxoethyl}methanesulfonamide **47**

The desired product was prepared according to the general procedure **E** with the following specific amounts: *tert*-butyl {2-[4-(2-ethoxyethoxy)phenyl]-2-oxoethyl}(methylsulfonyl)carbamate **44** (0.190 g, 0.473 mmol), DCM (5 mL), and TFA (0.7 mL). Column chromatography (EtOAc/hexane, 1:1) afforded *N*-{2-[4-(2-ethoxyethoxy)phenyl]-2-oxoethyl}methanesulfonamide **47** (0.120 g, 83%) as a white solid.

***R***_**f**_ = 0.35 (EtOAc/Hexane, 1:1); ^**1**^**H NMR (300 MHz, CDCl**_**3**_**) δ** 7.90 (d, *J* = 9.0, 2H, 2 × Ar*H*), 6.99 (d, *J* = 9.0, 2H, 2 × Ar*H*), 5.43 (t, *J* = 4.8 Hz, 1H, N*H*), 4.60 (d, *J* = 4.8 Hz, 2H, C*H*_2_), 4.19 (dd, *J* = 5.5, 4.1 Hz, 2H, OC*H*_2_CH_2_OCH_2_CH_3_), 3.81 (dd, *J* = 5.5, 4.1 Hz, 2H, OCH_2_C*H*_2_OCH_2_CH_3_), 3.60 (q, *J* = 6.9 Hz, 2H, C*H*_2_CH_3_), 2.98 (s, 3H), 1.24 (t, *J* = 6.9 Hz, 3H, CH_2_C*H*_3_); ^**13**^**C NMR (75 MHz, CDCl**_**3**_**) δ** 191.7 (*C*O), 164.0, 130.4 (2 × Ar*C*), 127.0, 115.0 (2 × ArC), 68.7, 68.0, 67.1, 48.9, 40.8, 15.2; **HRMS***m***/***z* (ESI^+^) calcd for C_13_H_20_NO_5_S (M+H^+^) 302.1062; found, 302.1063.

#### *N*-{2-[4-(2-Morpholinoethoxy)phenyl]-2-oxoethyl}methanesulfonamide **48**

The desired product was prepared according to the general procedure **E** with the following specific amounts: *tert*-butyl (methylsulfonyl){2-[4-(2-morpholinoethoxy)phenyl]-2-oxoethyl}carbamate **45** (0.150 g, 0.339 mmol), DCM (3 mL), and TFA (0.5 mL). Column chromatography (DCM/MeOH, 9:1) afforded *N*-{2-[4-(2-ethoxyethoxy)phenyl]-2-oxoethyl}methanesulfonamide **48** (0.101 g, 87%) as an off-white solid.

***R***_**f**_ = 0.45 (DCM/MeOH, 9:1); ^**1**^**H NMR (300 MHz, CDCl**_**3**_**) δ** 7.91 (d, *J* = 9.0, 2H, 2 × Ar*H*), 6.98 (d, *J* = 9.0, 2H, 2 × Ar*H*), 5.42 (s, 1H, N*H*), 4.60 (d, *J* = 4.3 Hz, 2H, C*H*_2_), 4.19 (t, *J* = 5.7 Hz, 2H, NCH_2_C*H*_2_O), 3.80–3.67 (m, 4H, N(CH_2_C*H*_2_)_2_O), 3.00 (s, 3H, CH_3_), 2.83 (t, *J* = 5.7 Hz, 2H, NC*H*_2_CH_2_O), 2.63–2.53 (m, 4H, N(C*H*_2_CH_2_)_2_O).^**13**^**C NMR (75 MHz, CDCl**_**3**_**) δ** 191.7 (*C*O), 163.8, 130.4 (2 × Ar*C*), 127.0, 114.9 (2 × Ar*C*), 67.0 (2 × *C*), 66.4, 57.5, 54.2 (2 × *C*), 48.9, 40.8. **HRMS***m***/***z* (ESI^+^) calcd for C_15_H_23_N_2_O_5_S (M+H^+^) 343.1328; found, 343.1336.

#### *N*-(2-Oxo-2-{4-[2-(pyrrolidin-1-yl)ethoxy]phenyl}ethyl)methanesulfonamide **49**

The desired product was prepared according to the general procedure E with the following specific amounts: *tert*-butyl (methylsulfonyl)(2-oxo-2-{4-[2-(pyrrolidin-1-yl)ethoxy]phenyl}ethyl)carbamate **46** (0.260 g, 0.610 mmol), DCM (6 mL), and TFA (1 mL). Column chromatography (DCM/MeOH, 9:1) afforded *N*-{2-[4-(2-ethoxyethoxy)phenyl]-2-oxoethyl}methanesulfonamide **49** (0.125 g, 63%) as an off-white solid.

***R***_**f**_ = 0.33 (DCM/MeOH, 9:1); ^**1**^**H NMR (300 MHz, CDCl**_**3**_**) δ** 7.91 (d, *J* = 9.0, 2H, 2 × Ar*H*), 6.99 (d, *J* = 9.0, 2H, 2 × Ar*H*), 5.41 (s, 1H, N*H*), 4.60 (s, 2H, C*H*_2_), 4.22 (t, *J* = 5.7 Hz, 2H, NCH_2_C*H*_2_O), 2.99 (s, 3H, CH_3_), 2.98 (t, *J* = 5.7 Hz, 2H, NC*H*_2_CH_2_O), 2.72–2.68 (m, 4H, N(C*H*_2_CH_2_)_2_), 1.91–1.80 (m, 4H, N(CH_2_C*H*_2_)_2_); ^**13**^**C NMR (75 MHz, CDCl**_**3**_**) δ** 191.7 (*C*O), 130.5 (2 × Ar*C*), 127.0, 115.0 (2 × Ar*C*), 67.4, 54.9 (2 × *C*), 54.9, 48.9, 40.8, 23.6 (2 × *C*); **HRMS***m***/***z* (ESI^+^) calcd for C_15_H_23_N_2_O_4_S (M+H^+^) 327.1379; found, 327.1389.

#### 2-Amino-5-[4-(2-ethoxyethoxy)benzoyl]-4-phenyl-1*H*-pyrrole-3-carboxamide 50

The desired product was prepared according to the general procedure A with the following specific amounts: *N*-{2-[4-(2-ethoxyethoxy)phenyl]-2-oxoethyl}methanesulfonamide **47** (0.077 g, 0.26 mmol, 1 equiv), benzaldehyde **3** (0.035 g, 0.33 mmol, 1.3 equiv), cyanoacetamide **4** (0.028 g, 0.33 mmol, 1.3 equiv), and K_2_CO_3_ (0.021 g, 0.15 mmol, 0.6 equiv) in EtOH (3 mL). The precipitate was purified via column chromatography (EtOAc/hexane/DCM/MeOH, 2:1.5:1.4:0.1) affording 2-amino-5-[4-(2-ethoxyethoxy)benzoyl]-4-phenyl-1*H*-pyrrole-3-carboxamide **50** (0.054 g, 54%) as a yellow solid.

***R***_**f**_ = 0.28 (EtOAc/Hexane/DCM/MeOH, 2:2:0.9:0.1); ^**1**^**H NMR (300 MHz, DMSO-*d*_6_) δ** 10.85 (br s, 1H, N*H*), 7.27–7.01 (m, 7H, 7 × Ar*H*), 6.68 (br s, 1H, N*H*_2_), 6.52 (d, *J* = 9.0 Hz, 2H, 2 × Ar*H*), 6.52 (s, 2H, N*H*_2_), 4.64 (br s, 1H, N*H*_2_), 4.00–3.93 (m, 2H, OC*H*_2_CH_2_OCH_2_CH_3_), 3.65–3.60 (m, 2H, OCH_2_C*H*_2_OCH_2_CH_3_), 3.48 (q, *J* = 6.9 Hz, 2H, C*H*_2_CH_3_), 1.12 (t, *J* = 6.9 Hz, 3H, CH_2_C*H*_3_); ^**13**^**C NMR (75 MHz, DMSO*-d*_6_) δ** 183.5 (*C*O), 167.7, 160.1, 149.5, 135.0, 132.0, 131.5 (2 × Ar*C*), 131.0 (2 × Ar*C*), 130.5, 128.4 (2 × Ar*C*), 127.9, 121.5, 113.5 (2 × Ar*C*), 99.0, 68.6, 67.7, 66.1, 15.6 (*C*H_3_); **HRMS***m***/***z* (ESI^+^) calcd for C_22_H_24_N_3_O_4_ (M+H^+^) 394.1767; found, 394.1769.

#### 2-Amino-5-[4-(2-morpholinoethoxy)benzoyl]-4-phenyl-1*H*-pyrrole-3-carboxamide **51**

The desired product was prepared according to the general procedure **A** with the following specific amounts: *N*-{2-[4-(2-morpholinoethoxy)phenyl]-2-oxoethyl}methanesulfonamide **48** (0.105 g, 0.307 mmol, 1 equiv), benzaldehyde **3** (0.042 g, 0.40 mmol, 1.3 equiv), cyanoacetamide **4** (0.034 g, 0.40 mmol, 1.3 equiv), and K_2_CO_3_ (0.025 g, 0.18 mmol, 0.6 equiv) in EtOH (6 mL). The precipitate and filtrate were purified separately via column chromatography (EtOAc/DCM/MeOH, 1.5:3.3:0.2) affording 2-amino-5-[4-(2-morpholinoethoxy)benzoyl]-4-phenyl-1*H*-pyrrole-3-carboxamide **51** (0.049 g, 38%) as a yellow solid.

***R***_**f**_ = 0.26 (EtOAc/DCM/MeOH, 1.5:3.3:0.2); ^**1**^**H NMR (300 MHz, DMSO-*d*_6_) δ** 10.83 (br s, 1H, N*H*), 7.19–7.01 (m, 7H, 7 × Ar*H*), 6.70 (br s, 1H, N*H*_2_), 6.51 (d, *J* = 8.7 Hz, 2H, 2 × Ar*H*), 6.33 (s, 2H, N*H*_2_), 4.64 (br s, 1H, N*H*_2_), 3.98 (t, *J* = 5.7 Hz, 2H, OC*H*_2_CH_2_N), 3.63–3.52 (m, 4H, N(CH_2_C*H*_2_)_2_O), 2.61 (t, *J* = 5.7 Hz, 2H, OCH_2_C*H*_2_N), 2.47–2.39 (m, 4H, N(C*H*_2_CH_2_)_2_O); ^**13**^**C NMR (75 MHz, DMSO-*d*_6_) δ** 183.1 (*C*O), 167.2, 159.6, 149.0, 134.5, 131.5, 131.1, 130.6 (2 × Ar*C*), 130.0 (2 × Ar*C*), 128.0 (2 × Ar*C*), 127.4, 121.1, 113.1 (2 × Ar*C*), 98.6, 66.2 (2 × *C*), 65.4, 56.7, 53.6 (2 × *C*); **HRMS***m***/***z* (ESI^+^) calcd for C_24_H_27_N_4_O_4_ (M+H^+^) 435.2032; found, 435.2032.

#### 2-Amino-4-phenyl-5-{4-[2-(pyrrolidin-1-yl)ethoxy]benzoyl}-1*H*-pyrrole-3-carboxamide **52**

The desired product was prepared according to the general procedure A with the following specific amounts: *N*-(2-oxo-2-{4-[2-(pyrrolidin-1-yl)ethoxy]phenyl}ethyl)methanesulfonamide **49** (0.095 g, 0.29 mmol, 1 equiv), benzaldehyde **3** (0.040 g, 0.38 mmol, 1.3 equiv), cyanoacetamide **4** (0.031 g, 0.38 mmol, 1.3 equiv), and K_2_CO_3_ (0.024 g, 0.18 mmol, 0.6 equiv) in EtOH (4 mL). The precipitate and filtrate were purified separately via column chromatography (DCM/MeOH, 9.2:0.8–8.5:1.5) affording 2-amino-4-phenyl-5-{4-[2-(pyrrolidin-1-yl)ethoxy]benzoyl}-1*H*-pyrrole-3-carboxamide **52** (0.053 g, 43%) as a yellow solid.

***R***_**f**_ = 0.18 (DCM/MeOH, 9:1); ^**1**^**H NMR (300 MHz, DMSO-*d*_6_) δ** 10.83 (br s, 1H, N*H*), 7.20–7.04 (m, 7H, 7 × Ar*H*), 6.71 (br s, 1H, N*H*_2_), 6.52 (d, *J* = 8.7 Hz, 2H, 2 × Ar*H*), 6.33 (s, 2H, N*H*_2_), 4.66 (br s, 1H, N*H*_2_), 3.98 (t, *J* = 5.7 Hz, 2H, OC*H*_2_CH_2_N), 2.78 (t, *J* = 5.7 Hz, 2H, OCH_2_C*H*_2_N), 2.63–2.53 (m, 4H, N(C*H*_2_CH_2_)_2_), 1.79–1.62 (m, 4H, N(CH_2_C*H*_2_)_2_). ^**13**^**C NMR (75 MHz, DMSO-*d*_6_) δ** 183.0 (*C*O), 167.2, 159.5, 149.0, 134.5, 131.5, 131.1, 130.6 (2 × Ar*C*), 130.0 (2 × Ar*C*), 128.0 (2 × Ar*C*), 127.4, 121.1, 113.1 (2 × Ar*C*), 98.6, 66.4, 53.9 (2 × *C*), 53.9, 23.1 (2 × *C*). **HRMS***m***/***z* (ESI^+^) calcd for C_24_H_27_N_4_O_3_ (M+H^+^) 419.2083; found, 419.2085.

#### 6-[4-(2-Ethoxyethoxy)benzoyl]-2-(pent-4-yn-1-yl)-5-phenyl-1,7-dihydro-4*H*-pyrrolo[2,3-*d*]pyrimidin-4-one **33**

The desired product was prepared according to the general procedure B with the following specific amounts: 2-amino-5-[4-(2-ethoxyethoxy)benzoyl]-4-phenyl-1*H*-pyrrole-3-carboxamide **50** (0.062 g, 0.16 mmol, 1 equiv), Na (0.036 g, 1.6 mmol, 10 equiv), 0.65 mM NaOEt (2.42 mL, 1.58 mmol), and ethyl hex-5-ynoate **6** (0.221 g, 1.58 mmol, 10 equiv). Upon completion, the reaction mixture was diluted with H_2_O (3 mL) and neutralized using 2 M HCl. The precipitate was then collected and purified by column chromatography (EtOAc/hexane/DCM/MeOH, 1.5:2:1.4:0.1) to afford 6-[4-(2-ethoxyethoxy)benzoyl]-2-(pent-4-yn-1-yl)-5-phenyl-1,7-dihydro-4*H*-pyrrolo[2,3-*d*]pyrimidin-4-one **33** (0.047 g, 64%) as a light yellow solid.

***R***_**f**_ = 0.41 (EtOAc/Hexane/DCM/MeOH, 1.5:2:1.4:0.1); **MP** 198–202 °C; ^**1**^**H NMR (300 MHz, DMSO-*d*_6_) δ** 12.53 (br s, 1H, N*H*), 11.89 (br s, 1H, N*H*), 7.45 (d, *J* = 9.0 Hz, 2H, 2 × Ar*H*), 7.28–7.16 (m, 2H, 2 × Ar*H*), 7.14–7.03 (m, 3H, 2 × Ar*H*), 6.70 (d, *J* = 9.0 Hz, 2H, 2 × Ar*H*), 4.06–4.00 (m, 2H, OC*H*_2_CH_2_OCH_2_CH_3_), 3.66–3.61 (m, 2H, OCH_2_C*H*_2_OCH_2_CH_3_), 3.47 (q, *J* = 6.9 Hz, 2H, C*H*_2_CH_3_), 2.83 (t, *J* = 2.6 Hz, 1H, CH_2_CH_2_CH_2_CC*H*), 2.72 (t, *J* = 7.2 Hz, 2H, C*H*_2_CH_2_CH_2_CCH), 2.32–2.24 (m, 2H, CH_2_CH_2_C*H*_2_CC*H*), 2.00–1.84 (m, 2H, CH_2_C*H*_2_CH_2_CCH), 1.11 (q, *J* = 6.9 Hz, 3H, CH_2_C*H*_3_); ^**13**^**C NMR (75 MHz, DMSO**-*d*_6_**) δ** 186.3 (*C*O), 161.5, 159.3, 158.3, 149.5, 132.8, 131.5 (2 × Ar*C*), 131.1 (2 × Ar*C*), 130.0, 127.5, 126.9 (2 × Ar*C*), 126.5, 125.3, 113.6 (2 × Ar*C*), 104.2, 83.9, 71.8, 68.0, 67.4, 65.7, 32.9, 25.7, 17.3, 15.1; **HRMS***m***/***z* (ESI^+^) calcd for C_28_H_28_N_3_O_4_ (M+H^+^) 470.2080; found, 470.2078; purity: 98%.

#### 6-[4-(2-Morpholinoethoxy)benzoyl]-2-(pent-4-yn-1-yl)-5-phenyl-1,7-dihydro-4*H*-pyrrolo[2,3-*d*]pyrimidin-4-one **34**

The desired product was prepared according to the general procedure B with the following specific amounts: 2-amino-5-[4-(2-morpholinoethoxy)benzoyl]-4-phenyl-1H-pyrrole-3-carboxamide **51** (0.054 g, 0.12 mmol, 1 equiv), Na (0.029 g, 1.2 mmol, 10 equiv), 0.65 mM NaOEt (1.91 mL, 1.24 mmol), and ethyl hex-5-ynoate **6** (0.174 g, 1.243 mmol, 10 equiv). Upon completion, the reaction mixture was diluted with H_2_O (3 mL) and neutralized using 2 M HCl. The precipitate was then collected and purified by column chromatography (EtOAc/DCM/MeOH, 1.5:3.3:0.2) to afford 6-[4-(2-morpholinoethoxy)benzoyl]-2-(pent-4-yn-1-yl)-5-phenyl-1,7-dihydro-4*H*-pyrrolo[2,3-*d*]pyrimidin-4-one **34** (0.043 g, 68%) as an off-white solid.

***R***_**f**_ = 0.43 (EtOAc/DCM/MeOH, 1.5:3.3:0.2); **MP** 207–210 °C; ^**1**^**H NMR (300 MHz, DMSO-*d*_6_) δ** 12.53 (br s, 1H, N*H*), 11.89 (br s, 1H, N*H*), 7.44 (d, *J* = 8.7 Hz, 2H, 2 × Ar*H*), 7.24–7.16 (m, 2H, 2 × Ar*H*), 7.11–7.04 (m, 3H, 3 × Ar*H*), 6.69 (d, *J* = 8.7 Hz, 2H, 2 × Ar*H*), 4.04 (t, *J* = 5.7 Hz, 2H, OC*H*_2_CH_2_N), 3.61–3.52 (m, 4H, N(CH_2_C*H*_2_)_2_O), 2.83 (t, *J* = 2.6 Hz, 1H, CH_2_CH_2_CH_2_CC*H*), 2.71 (t, *J* = 7.5 Hz, 2H, C*H*_2_CH_2_CH_2_CCH), 2.62 (t, *J* = 5.7 Hz, 2H, OCH_2_C*H*_2_N), 2.47–2.39 (m, 4H, N(C*H*_2_CH_2_)_2_O), 2.27 (td, *J* = 7.1, 2.6 Hz, 2H, CH_2_CH_2_C*H*_2_CCH), 1.97–1.85 (m, 2H, CH_2_C*H*_2_CH_2_CCH); ^**13**^**C NMR (75 MHz, DMSO-*d*_6_) δ** 186.3 (*C*O), 161.5, 159.3, 158.4, 149.5, 132.8, 131.5 (2 × Ar*C*), 131.1 (2 × Ar*C*), 129.9, 127.4, 126.9 (2 × Ar*C*), 126.5, 125.3, 113.7 (2 × Ar*C*), 104.2, 83.9, 71.8, 66.1 (2 × *C*), 65.6, 56.7, 53.6 (2 × *C*), 32.9, 25.7, 17.3; **HRMS***m***/***z* (ESI^+^) calcd for C_30_H_31_N_4_O_4_ (M+H^+^) 511.2345; found, 511.2345; purity: 95%.

#### 2-(Pent-4-yn-1-yl)-5-phenyl-6-{4-[2-(pyrrolidin-1-yl)ethoxy]benzoyl}-1,7-dihydro-4*H*-pyrrolo[2,3-*d*]pyrimidin-4-one **35**

The desired product was prepared according to the general procedure B with the following specific amounts: 2-amino-4-phenyl-5-{4-[2-(pyrrolidin-1-yl)ethoxy]benzoyl}-1*H*-pyrrole-3-carboxamide **52** (0.040 g, 0.096 mmol, 1 equiv), Na (0.022 g, 0.96 mmol, 10 equiv), 0.65 mM NaOEt (1.46 mL, 0.956 mmol), and ethyl hex-5-ynoate **6** (0.134 g, 0.956 mmol, 10 equiv). Upon completion, the reaction mixture was diluted with H_2_O (3 mL) and neutralized using 2 M HCl. Precipitate was then collected and purified by column chromatography (DCM/MeOH, 9:1) to afford 2-(pent-4-yn-1-yl)-5-phenyl-6-{4-[2-(pyrrolidin-1-yl)ethoxy]benzoyl}-1,7-dihydro-4*H*-pyrrolo[2,3-*d*]pyrimidin-4-one **35** (0.029 g, 62%) as an off-white solid.

**R**_**f**_ = 0.22 (DCM/MeOH, 9:1); **MP** 240–244 °C; ^**1**^**H NMR (300 MHz, DMSO-*d*_6_) δ** 12.53 (br s, 1H, N*H*), 11.89 (br s, 1H, N*H*), 7.44 (d, *J* = 8.7 Hz, 2H, 2 × Ar*H*), 7.24–7.16 (m, 2H, 2 × Ar*H*), 7.11–7.04 (m, 3H, 3 × Ar*H*), 6.69 (d, *J* = 8.7 Hz, 2H, 2 × Ar*H*), 4.03 (t, *J* = 5.7 Hz, 2H, OC*H*_2_CH_2_N), 2.83 (t, *J* = 2.6 Hz, 1H, CH_2_CH_2_CH_2_CC*H*), 2.79–2.67 (m, 4H, OC*H*_2_CH_2_N/CH_2_CH_2_C*H*_2_CCH), 2.55–2.50 (m, 4H, N(C*H*_2_CH_2_)_2_), 2.27 (td, *J* = 7.1, 2.6 Hz, 2H, CH_2_CH_2_C*H*_2_CCH), 1.97–1.84 (m, 2H, CH_2_C*H*_2_CH_2_CCH), 1.73–1.63 (m, 4H, N(CH_2_C*H*_2_)_2_); ^**13**^**C NMR (75 MHz, DMSO-*d*_6_) δ** 186.3 (*C*O), 161.5, 159.3, 158.4, 149.5, 132.8, 131.5 (2 × Ar*C*), 131.1 (2 × Ar*C*), 129.9, 127.5, 126.9 (2 × Ar*C*), 126.5, 125.3, 113.7 (2 × Ar*C*), 104.2, 83.9, 71.8, 66.8, 54.0 (2 × *C*), 32.9, 25.7, 23.1 (2 × *C*), 17.3; **HRMS***m***/***z* (ESI^+^) calcd for C_30_H_31_N_4_O_3_ (M+H^+^) 495.2396; found, 495.2394; purity: 97%.

#### 2-Amino-5-[4-(2-ethoxyethoxy)benzoyl]-4-(3-fluorophenyl)-1*H*-pyrrole-3-carboxamide **56**

The desired product was prepared according to the general procedure **A** with the following specific amounts: *N*-{2-[4-(2-ethoxyethoxy)phenyl]-2-oxoethyl}methanesulfonamide **47** (0.085 g, 0.28 mmol, 1 equiv), 3-fluorobenzaldehyde **53** (0.046 g, 0.37 mmol, 1.3 equiv), cyanoacetamide **4** (0.031 g, 0.37 mmol, 1.3 equiv), and K_2_CO_3_ (0.023 g, 0.17 mmol, 0.6 equiv) in EtOH (3 mL). The precipitate was purified via column chromatography (EtOAc/hexane/DCM/MeOH, 2:2:0.9:0.1) affording 2-amino-5-[4-(2-ethoxyethoxy)benzoyl]-4-(3-fluorophenyl)-1*H*-pyrrole-3-carboxamide **56** (0.067 g, 58%) as a yellow solid.

***R***_**f**_ = 0.32 (EtOAc/Hexane/DCM/MeOH, 2:2:0.9:0.1); ^**1**^**H NMR (400 MHz, DMSO-*d*_6_) δ** 10.90 (br s, 1H, N*H*), 7.25–7.20 (m, 1H, 1 × Ar*H*), 7.16 (d, *J* = 8.7 Hz, 2H, 2 × Ar*H*), 7.02–6.92 (m, 2H, 2 × Ar*H*), 6.90–6.84 (m, 1H, 1 × Ar*H*), 6.58 (d, *J* = 8.7 Hz, 2H, 2 × Ar*H*), 6.30 (s, 2H, N*H*_2_), 4.03–3.97 (m, 2H, OC*H*_2_CH_2_OCH_2_CH_3_), 3.67–3.61 (m, 2H, OCH_2_C*H*_2_OCH_2_CH_3_), 3.48 (q, *J* = 7.0 Hz, 2H, C*H*_2_CH_3_), 1.13 (t, *J* = 7.0 Hz, 3H, CH_2_C*H*_3_); ^**13**^**C NMR (151 MHz, DMSO-*d*_6_) δ** 183.0 (CO), 167.0, [162.3, 160.7] (d, J = 244.5 Hz, ArC–F), 159.8, 148.9, 136.8 (d, *J* = 8.3 Hz), 131.6, 129.9 (2 × Ar*C*), [129.9, 129.8]* (d, *J* = 8.5 Hz) 129.6, 126.8, 121.1, [117.6, 117.5] (d, *J* = 21 Hz), [114.3, 114.2] (d, *J* = 21 Hz), 113.1 (2 × Ar*C*), 98.6, 68.1, 67.3, 65.7, 15.1; **HRMS***m***/***z* (ESI^+^) calcd for C_22_H_23_FN_3_O_4_ (M+H^+^) 412.1673; found, 412.1686.

#### 2-Amino-4-(3,5-difluorophenyl)-5-[4-(2-ethoxyethoxy)benzoyl]-1*H*-pyrrole-3-carboxamide **57**

The desired product was prepared according to the general procedure A with the following specific amounts: *N*-{2-[4-(2-ethoxyethoxy)phenyl]-2-oxoethyl}methanesulfonamide **47** (0.040 g, 0.13 mmol, 1 equiv), 3,5-difluorobenzaldehyde **54** (0.025 g, 0.17 mmol, 1.3 equiv), cyanoacetamide **4** (0.014 g, 0.17 mmol, 1.3 equiv), and K_2_CO_3_ (0.011 g, 0.078 mmol, 0.6 equiv) in EtOH (2 mL). The precipitate was purified via column chromatography (EtOAc/hexane/DCM/MeOH, 2:2:0.9:0.1) affording 2-amino-4-(3,5-difluorophenyl)-5-[4-(2-ethoxyethoxy)benzoyl]-1*H*-pyrrole-3-carboxamide **57** (0.035 g, 63%) as a yellow solid.

***R***_**f**_ = 0.43 (EtOAc/hexane/DCM/MeOH, 2:2:0.9:0.1); ^**1**^**H NMR (600 MHz, DMSO*-****d*_6_**) δ** 10.94 (br s, 1H, N*H*), 7.19 (d, *J* = 8.4 Hz, 2H, 2 × Ar*H*), 7.09–6.97 (m, 1H, Ar*H*), 6.81–6.76 (m, 2H, 2 × Ar*H*), 6.64 (d, *J* = 8.4 Hz, 2H, 2 × Ar*H*), 6.25 (s, 2H, N*H*_2_), 4.04–4.00 (m, 2H, OC*H*_2_CH_2_OCH_2_CH_3_), 3.67–3.64 (m, 2H, OCH_2_C*H*_2_OCH_2_CH_3_), 3.49 (q, *J* = 7.0 Hz, 2H, C*H*_2_CH_3_), 1.13 (t, *J* = 7.0 Hz, 3H, CH_2_C*H*_3_); ^**13**^**C NMR (151 MHz, DMSO-*d*_6_) δ** 183.0, 166.8, [162.6, 162.5, 160.9, 160.8] (dd, *J* = 246.9, 13.9 Hz, 2 × Ar*C*-F), 159.9, 148.7, 137.9 (t, *J* = 10.3 Hz), 131.7, 129.9 (2 × Ar*C*), 128.5, 121.0, [114.2, 114.1, 114.0] (dd, *J* = 25.2, 5.3 Hz, 2 × Ar*C*C–F), 113.1 (2 × Ar*C*), 102.8 (t, *J* = 25.2 Hz), 98.8, 68.1, 67.3, 65.7, 15.1; **HRMS***m***/***z* (ESI^+^) calcd for C_22_H_22_F_2_N_3_O_4_ (M+H^+^) 430.1578; found, 430.1580.

#### 2-Amino-5-[4-(2-ethoxyethoxy)benzoyl]-4-(6-fluoropyridin-2-yl)-1H-pyrrole-3-carboxamide **58**

The desired product was prepared according to the general procedure A with the following specific amounts: *N*-{2-[4-(2-ethoxyethoxy)phenyl]-2-oxoethyl}methanesulfonamide **47** (0.040 g, 0.13 mmol, 1 equiv), 6-fluoropicolinaldehyde **55** (0.021 g, 0.17 mmol, 1.3 equiv), cyanoacetamide **4** (0.014 g, 0.17 mmol, 1.3 equiv), and K_2_CO_3_ (0.011 g, 0.078 mmol, 0.6 equiv) in EtOH (2 mL). Purification via column chromatography (EtOAc/hexane/DCM/MeOH, 2:2:0.9:0.1) proved to be very challenging with multiple side products present. The crude mixture (42%, orange solid) was thus used for the next step without further purification.

#### 6-[4-(2-Ethoxyethoxy)benzoyl]-5-(3-fluorophenyl)-2-(pent-4-yn-1-yl)-1,7-dihydro-4*H*-pyrrolo[2,3-*d*]pyrimidin-4-one **59**

The desired product was prepared according to the general procedure B with the following specific amounts: 2-amino-5-[4-(2-ethoxyethoxy)benzoyl]-4-(3-fluorophenyl)-1*H*-pyrrole-3-carboxamide **56** (0.060 g, 0.15 mmol, 1 equiv), Na (0.034 g, 1.46 mmol, 10 equiv), 0.65 mM NaOEt (2.2 mL, 1.46 mmol), and ethyl hex-5-ynoate **6** (0.205 g, 1.46 mmol, 10 equiv). Upon completion, the reaction mixture was diluted with H_2_O (3 mL) and neutralized using 2 M HCl. Precipitate was then collected and purified by column chromatography (EtOAc/hexane/DCM/MeOH, 1.5:2:1.4:0.1) to afford 6-[4-(2-ethoxyethoxy)benzoyl]-5-(3-fluorophenyl)-2-(pent-4-yn-1-yl)-1,7-dihydro-4*H*-pyrrolo[2,3-*d*]pyrimidin-4-one **59** (0.053 g, 75%) as a light yellow solid.

***R***_**f**_ = 0.40 (EtOAc/hexane/DCM/MeOH, 4:4:1.5:0.5); **MP** 224–227 °C; ^**1**^**H NMR (600 MHz, DMSO-*d*_6_) δ** 12.62 (br s, 1H, N*H*), 11.94 (br s, 1H, N*H*), 7.47 (d, *J* = 8.9 Hz, 2H, 2 × Ar*H*), 7.12–7.05 (m, 2H, 2 × Ar*H*), 6.97 (d, *J* = 7.7 Hz, 1H, Ar*H*), 6.924–6.90 (m, 1H, Ar*H*), 6.75 (d, *J* = 8.9 Hz, 2H, 2 × Ar*H*), 4.07–4.03 (m, 2H, OC*H*_2_CH_2_OCH_2_CH_3_), 3.67–3.62 (m, 2H, OCH_2_C*H*_2_OCH_2_CH_3_), 3.47 (q, *J* = 7.0 Hz, 2H, C*H*_2_CH_3_), 2.82 (t, *J* = 2.6 Hz, 1H, CH_2_CH_2_CH_2_CC*H*), 2.72 (t, *J* = 7.5 Hz, 2H, C*H*_2_CH_2_CH_2_CCH), 2.28 (td, *J* = 7.2, 2.6 Hz, 2H, CH_2_CH_2_C*H*_2_CC*H*), 1.95–1.88 (m app. p, *J* = 7.2 Hz, 2H, CH_2_C*H*_2_CH_2_CCH), 1.11 (t, *J* = 7.0 Hz, 3H, CH_2_C*H*_3_); ^**13**^**C NMR (151 MHz, DMSO-*d*_6_) δ** 186.1, [161.9, 160.3] (d, *J* = 242.2 Hz, ArC–F), 161.7, 159.3, 158.5, 149.5, [135.2, 135.1] (d, *J* = 8.6 Hz), 131.5 (2 × ArC), 130.0, [128.7, 128.6] (d, *J* = 8.5 Hz), 127.7, 127.2, 123.7 (d, *J* = 2.1 Hz), [117.7, 117.6] (d, *J* = 22 Hz), 113.7 (2 × ArC), [113.4, 113.2] (d, *J* = 20.8 Hz), 104.2, 83.8, 71.7, 68.2, 67.5, 65.7, 32.9, 25.7, 17.3, 15.1; **HRMS***m***/***z* (ESI^+^) calcd for C_28_H_27_FN_3_O_4_ (M+H^+^) 489.2069; found, 489.2043; purity: 94%.

#### 5-(3,5-Difluorophenyl)-6-[4-(2-ethoxyethoxy)benzoyl]-2-(pent-4-yn-1-yl)-1,7-dihydro-4*H*-pyrrolo[2,3-*d*]pyrimidin-4-one **60**

The desired product was prepared according to the general procedure B with the following specific amounts: 2-amino-4-(3,5-difluorophenyl)-5-[4-(2-ethoxyethoxy)benzoyl]-1*H*-pyrrole-3-carboxamide **57** (0.050 g, 0.12 mmol, 1 equiv), Na (0.027 g, 1.16 mmol, 10 equiv), 0.65 mM NaOEt (1.8 mL, 1.16 mmol), and ethyl hex-5-ynoate **6** (0.163 g, 1.16 mmol, 10 equiv). Upon completion, the reaction mixture was diluted with H_2_O (3 mL) and neutralized using 2 M HCl. Precipitate was then collected and purified by column chromatography (EtOAc/Hexane/DCM/MeOH, 1.5:2:1.4:0.1) to afford 5-(3,5 difluorophenyl)-6-[4-(2-ethoxyethoxy)benzoyl]-2-(pent-4-yn-1-yl)-1,7-dihydro-4*H*-pyrrolo[2,3-*d*]pyrimidin-4-one **60** (0.032 g, 55%) as a light yellow solid.

***R***_**f**_ = 0.52 (EtOAc/Hexane/DCM/MeOH, 4:4:1.5:0.5); **MP** 220–225 °C; ^**1**^**H NMR (600 MHz, DMSO-*d*_6_) δ** 12.71 (br s, 1H, N*H*), 11.98 (br s, 1H, N*H*), 7.49 (d, *J* = 8.8 Hz, 2H, 2 × Ar*H*), 6.99–6.88 (m, 3H, 3 × Ar*H*), 6.80 (d, *J* = 8.8 Hz, 2H, 2 × Ar*H*), 4.10–4.06 (m, 2H, OC*H*_2_CH_2_OCH_2_CH_3_), 3.68–3.64 (m, 2H, OCH_2_C*H*_2_OCH_2_CH_3_), 3.48 (q, *J* = 7.2 Hz, 2H, C*H*_2_CH_3_), 2.82 (t, *J* = 2.6 Hz, 1H, CH_2_CH_2_CH_2_CC*H*), 2.73 (t, *J* = 7.2 Hz, 2H, C*H*_2_CH_2_CH_2_CCH), 2.28 (td, *J* = 7.2, 2.6 Hz, 2H, CH_2_CH_2_C*H*_2_CC*H*), 1.95–1.87 (m app. p, *J* = 7.2 Hz, 2H, CH_2_C*H*_2_CH_2_CCH), 1.12 (t, *J* = 7.2 Hz, 2H, CH_2_C*H*_3_); ^**13**^**C NMR (151 MHz, DMSO**-*d*_6_**) δ** 186.0, [162.0, 161.9, 160.4, 160.3] (dd, J = 244.5, 13.6 Hz, 2 × Ar*C*–F), 161.8, 159.2, 158.7, 149.5, 136.4 (t, J = 10.7 Hz), 131.5 (2 × ArC), 130.0, 128.0, 122.6, [114.2, 114.1] (dd, J = 25.5, 5.0 Hz, 2 × Ar*C*C–F), 113.8 (2 × ArC), 104.2, 101.9 (t, J = 25.5 Hz), 83.8, 71.8, 68.0, 67.5, 65.70, 32.9, 25.7, 17.3, 15.0; **HRMS***m***/***z* (ESI^+^) calcd for C_28_H_25_F_2_N_3_O_4_ (M+H^+^) 506.1891, found 506.1892; Purity: 95%

#### 6-[4-(2-Ethoxyethoxy)benzoyl]-5-(6-fluoropyridin-2-yl)-2-(pent-4-yn-1-yl)-1,7-dihydro-4*H*-pyrrolo[2,3-*d*]pyrimidin-4-one **61**

The desired product was prepared according to the general procedure B with the following specific amounts: crude mixture containing 2-amino-4-(3,5-difluorophenyl)-5-[4-(2-ethoxyethoxy)benzoyl]-1*H*-pyrrole-3-carboxamide **58** (0.060 g, 0.15 mmol, 1 equiv), Na (0.033 g, 1.45 mmol, 10 equiv), 0.65 mM NaOEt (2.3 mL, 1.16 mmol), and ethyl hex-5-ynoate **6** (0.203 g, 1.45 mmol, 10 equiv). Upon completion, the reaction mixture was diluted with H_2_O (3 mL) and neutralized using 2 M HCl. The precipitate was then collected and purified by column chromatography (EtOAc/hexane/DCM/MeOH, 1.5:2:1.4:0.1) to afford 6-[4-(2-ethoxyethoxy)benzoyl]-5-(6-fluoropyridin-2-yl)-2-(pent-4-yn-1-yl)-1,7-dihydro-4*H*-pyrrolo[2,3-*d*]pyrimidin-4-one **61** (0.023 g, 31%) as a light orange solid, which proved to be unstable over time.

***R***_**f**_ = 0.37 (EtOAc/Hexane/DCM/MeOH, 4:4:1.5:0.5); ^**1**^**H NMR (400 MHz, DMSO-*d*_6_) δ** 12.80 (br s, 1H, N*H*), 12.06 (br s, 1H, N*H*), 8.19 (d, *J* = 7.7 Hz, 1H, 2 × Ar*H*), 7.66–7.62 (m, 1H, Ar*H*), 7.50 (d, *J* = 7.7 Hz, 2H, 2 × Ar*H*), 7.23 (d, *J* = 7.9 Hz, 1H, Ar*H*), 6.80 (d, 2H, 2 × Ar*H*), 4.11–4.04 (m, 2H, OC*H*_2_CH_2_OCH_2_CH_3_), 3.69–3.62 (m, 2H, OCH_2_C*H*_2_OCH_2_CH_3_), 3.48 (q, *J* = 7.0 Hz, 2H, C*H*_2_CH_3_), 2.83 (t, *J* = 2.6 Hz, 1H, CH_2_CH_2_CH_2_CC*H*), 2.73 (t, *J* = 7.5 Hz, 2H, C*H*_2_CH_2_CH_2_CCH), 2.28 (td, *J* = 7.1, 2.6 Hz, 2H, CH_2_CH_2_C*H*_2_CC*H*), 1.92 (p, *J* = 7.3 Hz, 2H, CH_2_C*H*_2_CH_2_CCH), 1.11 (t, *J* = 7.0 Hz, 3H, CH_2_C*H*_3_); ^**13**^**C NMR (101 MHz, DMSO-*d*_6_) δ** 187.1, 161.6, 159.5, 158.0, 152.7, 149.6, 139.3, 138.8, 130.9 (2 × ArC), 130.5, 130.1, 125.1, 124.3, 120.8, 113.8 (2 × ArC), 103.3, 83.8, 71.8, 68.1, 67.5, 65.7, 32.81, 25.6, 17.3, 15.1. Compound **61** proved to be unstable and was not investigated any further.

##### Crystallography

Single-crystal X-ray intensity data were collected on a Bruker 3-circle SMART Apex II X-ray diffractometer equipped with an INCOATEC IμS HB microfocus sealed tube (Mo Kα radiation λ = 0.71073 Å) fitted with a multilayer monochromator. Data were captured with a CCD (charge-coupled device) area detector. Data collection was carried out at 100 K using an Oxford Cryosystems cryostat (700 series Cryostream Plus) attached to the diffractometer. Data collection and reduction were carried out using the Bruker software package APEX3, using standard procedures.^2^ All structures were solved and refined using SHELX-2018 employed within the X-Seed environment.^3−7^ Hydrogen atoms were placed in calculated positions using riding models. Diagrams were generated using POV-Ray.^8^

### Cellular Evaluation

#### Cell Culture

Human cervical adenocarcinoma (HeLa) cells were obtained from the American Type Culture Collection (ATCC) and cultured in Dulbecco’s modified Eagle’s medium (DMEM) supplemented with 10% fetal bovine serum (FBS), 4 mM glutamine (Lonza code BE17–605E), 100 μg/mL gentamicin (Lonza code 17–5182), and penicillin–streptomycin (200 units/mL and 200 μg/mL) (Lonza code 17–602E). All cell lines were cultured in T25 flasks, maintained and grown at 37 °C, 95% humidity, and 5% CO_2_.

#### Antiproliferative Properties

The 3-(4,5-dimethylthiazol-2-yl)-2,5-diphenyltetrazolium bromide (MTT) assay was employed to evaluate the antiproliferative activities of the synthesized 7-deazahypoxathine analogues. The HeLa, PNT1A, or PANC-1 cell lines were assessed by trypsinizing and seeding 4 × 10^3^ cells per well into 96-well plates. Synthesized compounds were dissolved in DMSO at a concentration of either 50 mM or 100 mM before cell treatment. Cells were grown for 24 h and then treated with compounds at concentrations ranging from 0.04 to 100 μM and incubated for 48 h in 200 μL media. Twenty microliters of MTT reagent in serum-free medium (5 mg/mL) was added to each well and incubated further for 2 h. Media was removed and the resulting formazan crystals were resolubilized in 200 μL of DMSO. A Thermomax Molecular Device plate reader was utilized to measure *A*_490_. Experiments were performed in quadruplicate and repeated at least twice for each compound. The control comprised of cells treated with 0.1% DMSO. One micromolar phenyl arsine oxide (PAO) was utilized as a positive killing control.

For compounds **1**, **59**, and **60**, the same method was used except for the following deviations: (1) HeLa cells were plated at a seeding density of 2.5 × 10^3^ cells per well in a 96-well plate and (2) cells were treated with a concentration of compound ranging from 1 μM to 7.8 nM in a total volume of 100 μL media for 48 h. Thereafter, 10 μL of MTT in phosphate-buffered saline (PBS) (5 mg/mL) was added and incubated with the cells for 4 h. Thereafter, 100 μL of 0.1 M SDS in 0.01 M HCL was added to solubilize the formazan crystals, overnight at 37 °C. Experiments were performed in triplicate and repeated as indicated for each compound.

To directly assess apoptosis in living cells, HeLa cells were plated (six biological replicates per condition) in 96-well Imagelock Plates (Sartorius BA-04855) at a density of 1,562.5 cells/cm^2^. The following day, cells were placed in media containing IncuCyte Annexin V Dye (Sartorius 4642) supplemented with either 0.1% Dimethyl sulfoxide or test compounds (20 nM), and plates were placed in a Sartorius Incucyte S3 live cell imaging system. Plates were scanned hourly, with phase contrast and green fluorescence images were acquired at three positions per well. Raw fluorescence data was processed using Incucyte software, and Prism Graphpad was used for data visualization and statistical analysis. To track individual cell fates, combined phase contrast/fluorescence time lapse sequences were exported from multiple wells per condition, and cell fates individual cells were scored and tabulated using Graphpad Prism software.

To examine microtubule morphology in rigidin-treated cells, HeLa cells were incubated for 4 h in the presence or absence of test compound before being fixed with 3.7% formaldehyde in PBS and permeabilized with 0.1% Triton X-100 in PBS. After blocking the cells with 3% bovine serum albumin in PBS, they were probed with antibodies for the centromere marker CENP-B (Abcam, code ab25734) and tubulin (Sigma, code sc-53029), which were detected with Alexa Fluor-labeled secondary antibodies (Thermofisher). Hoechst 33342 (1 mg/mL, Thermofisher H3570) was used to stain DNA. Samples were mounted on glass slides and imaged using Andor Dragonfly 505 spinning disk confocal system mounted on an Olympus IX83 inverted microscope. Figures were created with Adobe Photoshop CS5. All drugs were resuspended in DMSO, and 0.1% DMSO served as a carrier control.

#### Tubulin Polymerization Assay

The fluorescence-based tubulin polymerization assay from Cytoskeleton, Inc. was used. Aqueous solutions of the tested agents (**1**, **33**, **34**, and **59**, paclitaxel and colchicine) were prepared at a 10× concentration in 10% DMSO. The tubulin reaction mixture was prepared per manufacturer’s instructions, kept on ice, and used within ∼15 min of preparation. A Biotek Synergy H4 hybrid multimode plate reader was preheated to 37 °C. Prior to starting the assay, a half-area black 96-well plate was preheated within the plate reader. A volume of 5 μL of each of the tested samples was pipetted into separate wells of the plate and incubated in the warm plate reader for 1 min. A volume of 45 μL of the tubulin reaction mixture was then mixed into each of the wells, thereby diluting the samples to their final concentration (**1**, **33**, **34**, and **59** at 25 μM, paclitaxel at 3 μM, colchicine at 6 μM, 1% DMSO control). The fluorescence of the samples (λ_Ex_ = 360/20 nm, λ_Em_ = 485/20 nm) was then recorded every minute for 1 h. Paclitaxel was used as a tubulin polymerization inducing control, colchicine as a tubulin polymerization inhibitor, and 1% DMSO was used as a carrier control.

#### Live Cell Imaging

HeLa cells were plated on 35 mm glass-bottomed Mattek dishes (Mattek, code P35G-1.5–14-C). Next day, transient transfection of EGFP-tubulin was performed using a Lipofectamine 2000 (Thermofisher code 11668019) according to the manufacturer’s instructions. After 6 h of transfection, cells were cultured in fresh media overnight before being treated with either 0.1% DMSO or 7.5 nM analogue **33** for 4 h. To visualize chromatin, cells were cultured in the presence the cell permeant NucSpot Live nuclear stain (Biotium code 40082) for 60 min prior to imaging. Cells were imaged live using an Andor Dragonfly 505 spinning disk confocal system mounted on an Olympus IX83 inverted microscope. Cell viability was maintained by an Oko Touch prewarmed (37 °C) temperature-controlled microscope stage supplied with 5% CO_2_. Z-stacks (10 planes at 1.36 mm intervals) were acquired every 2 min using a 60× Aprochromat silicon objective (NA 1.30) and an Andor iXon 888 EMCCD camera driven by Andor Fusion software. Fusion IMS files were converted to tiff images and avi movies using FIJI, and figures were prepared in Adobe Photoshop CS5.

#### Molecular Modeling

Molecular modeling studies were carried out using the Schrödinger modeling suite (version 2021-1). The receptor utilized in the study was obtained from the PDB (3UT5).^9^ For the purposes of docking, a glide grid was generated using the cocrystallized ligand (colchicine), and then, compound **33** was docked using Glide-SP. Nonstandard parameters employed included “use enhanced sampling” (4×) and “use expanded sampling”. The highest scoring pose (Figure 5) was selected as the most likely binding pose for compound **33**. Since the pyrrolopyrimidine core can exist in various tautomeric states, low energy tautomers were obtained using the Schrödinger QM Tautomer and Conformer predictor tool and investigated using the docking procedure described.

## Data Availability

Authors will release the atomic coordinates of these structures upon article publication.

## Supporting Information Available

^1^H NMR spectra of compound **1** (terminal alkyne) and **31**; HeLa cells programmed cell death analysis; immunolabeling studies; cell fates of cells treated with lead compounds; cytotoxicity GI_50_s of selected compounds against HeLa, PANC1, and PNT1A cells; ^1^H and ^13^C NMR spectra of the final 7-deazahypoxanthine compounds: **1**, **13**, **15**, **21**, **27**, **30**, **31**, **33–35**, and **59–61**; LCMS purity analysis of the final 7-deazahypoxanthine compounds: **13**, **15**, **21**, **27**, **30**, **31**, **33–35**, and **59–61** (PDF)
HeLa cell and control (AVI)
HeLa cell and compound **33** (AVI)
Molecular formula strings (CSV)

## Supplementary Material

jm3c02473_si_001.pdf

jm3c02473_si_002.avi

jm3c02473_si_003.avi

## Abbreviations

amu: atomic mass unit
ATR: attenuated total reflectance
ATCC: American-type culture collection
CAF: Central Analytical Facility
CCD: charge-coupled device
CCDC: Cambridge Crystallographic Data Center
DMEM: Dulbecco's modified Eagle's medium
EGFP: enhanced green fluorescent protein
FBS: fetal bovine serum
GI_50_: growth inhibitory dose 50%
MTT: 3-(4,5-dimethylthiazol-2-yl)-2,5-diphenyltetrazolium bromide
PAO: phenyl arsine oxide
PTAB: phenyltrimethylammonium tribromide
SD: standard deviation
UPLC: ultraperformance liquid chromatography
WSG: water-solubilizing group
