# Supplementary material for: Improved Rigidin-Inspired Antiproliferative Agents with Modifications on the 7-Deazahypoxanthine C7/C8 Ring Systems
Source: J Med Chem. 2024 Jun 12;67(12):9950–75. doi: 10.1021/acs.jmedchem.3c02473 (PMC11215747; doi:10.1021/acs.jmedchem.3c02473)
Supplement: Supplementary file 1 — jm3c02473_si_001.pdf [file jm3c02473_si_001.pdf]

Supplementary Information

Improved rigidin-inspired antiproliferative agents with modifications on the 7-deazahypoxanthine C7/C8 ring systems

Aletta E. van der Westhuyzen,[a] Naghmana Ashraf,[b] Daleen Conradie,[a,c] Leigh Loots,[a] Catherine H. Kaschula,[a] Stephen C. Pelly,[a,d] Liliya V. Frolova,[e] Taylor Landfair,[b] Charles B. Shuster,[b] Tania Betancourt,[e] Alexander Kornienko,[e] Willem A. L. van Otterlo*,[a]

[a]*Department of Chemistry and Polymer Science, Stellenbosch University, Stellenbosch, 7600, South Africa*
[b]*Department of Biology, New Mexico State University, Las Cruces, NM 88003, United States*
[c]*Department of Physiological Sciences, Stellenbosch University, Stellenbosch, 7600, South Africa*
[d]*Department of Chemistry, Emory University, 1515 Dickey Drive, Atlanta, GA 30322, United States*
[e] *Department of Chemistry and Biochemistry, Purdue University, 2101 East Coliseum Blvd., Fort Wayne, IN 46805, United States*
[e]*Department of Chemistry and Biochemistry, Texas State University, San Marcos, TX 78666, United States*

* wvo@sun.ac.za

CONTENT OF SI

**Page S2.**	**Fig. S1. ^{1}H NMR spectra of compound 1 (terminal alkyne) and 31**
**Page S3.**	**Fig. S2. HeLa cells programmed cell death analysis**
**Page S4.**	**Fig. S3. Immunolabeling studies**
**Page S5a.**	**Fig. S4. Cell fates of cells treated with lead compounds**
**Page S5b.**	**Movie S1-HeLa cell and Control**
**Page S5c.**	**Movie S2-HeLa cell and Compound 33**
**Page S6.**	**Table S1. Cytotoxicity GI_{50}s of selected compounds against HeLa, PANC1 and PNT1A cells**
**Pages S7-S19.**	**^{1}H & ^{13}C NMR spectra of the final 7-deazahypoxanthine compounds: 1, 13, 15, 21, 27, 30, 31, 33-35, 59-61**
**Pages S20-S25.**	**LCMS purity analysis of the final 7-deazahypoxanthine compounds: 13, 15, 21, 27, 30, 31, 33-35, 59-61**
**Page S26.**	**References**

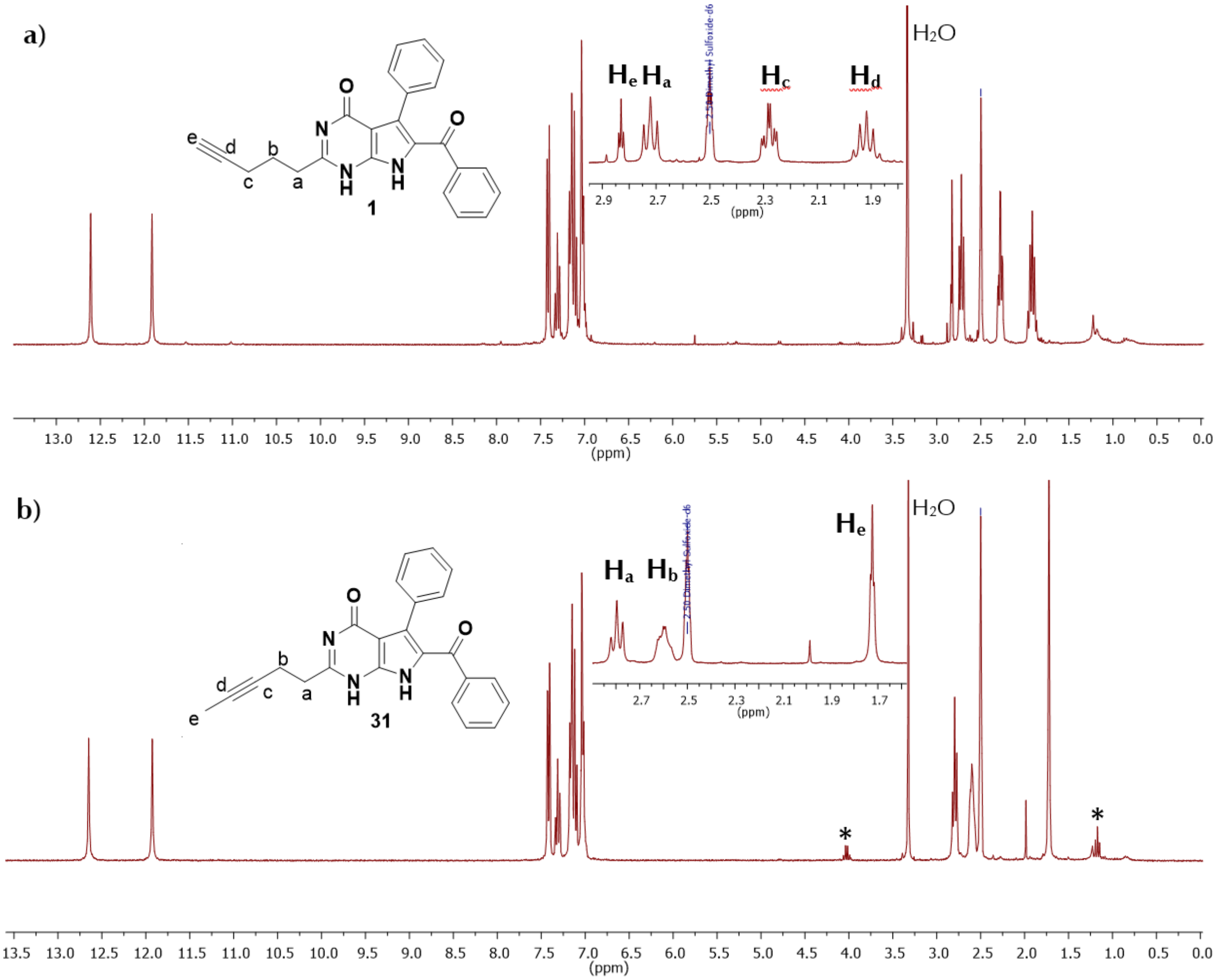

Figure S1: ^{1}H NMR spectra of a) compound **1** (terminal alkyne) and b) **31** (internal alkyne). * = ethyl acetate

HeLa cells programmed cell death analysis

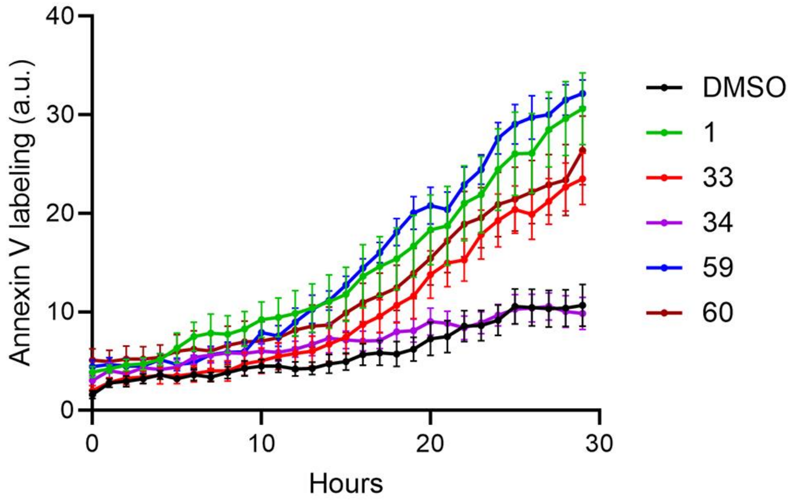

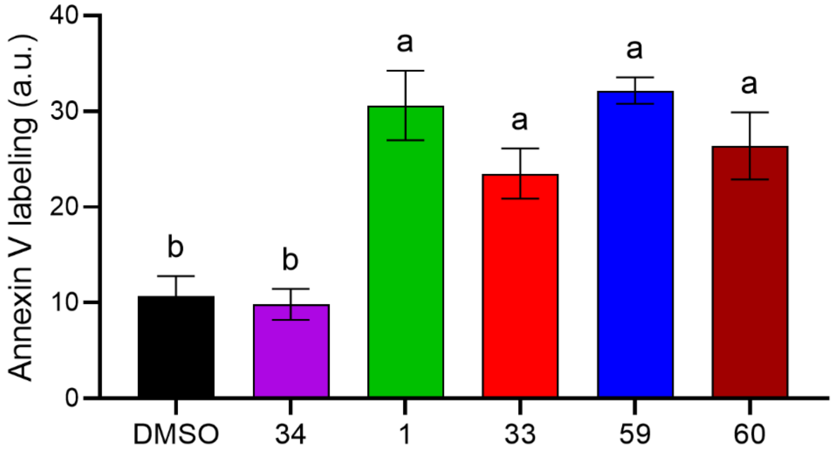

Figure S2. Real-time analysis of programmed cell death of HeLa cells treated with rigidin compounds. **(Top)** HeLa cells were cultured in the presence of 0.1% DMSO or 20 nM compounds (**1**, **33**, **34**, **59**, **60**) and green fluorescent Annexin V to mark apoptotic cells, and the increase in cell death was monitored over a 30 hour period. Error bars, SEM. (**Bottom**) Quantification of apoptosis at the 30 hour time point (6 biological replicates per condition). One-way ANOVA analysis found no significant differences amongst compounds **1**, **33**, **59** and **60**. Error bars, SEM.

Immunolabeling studies

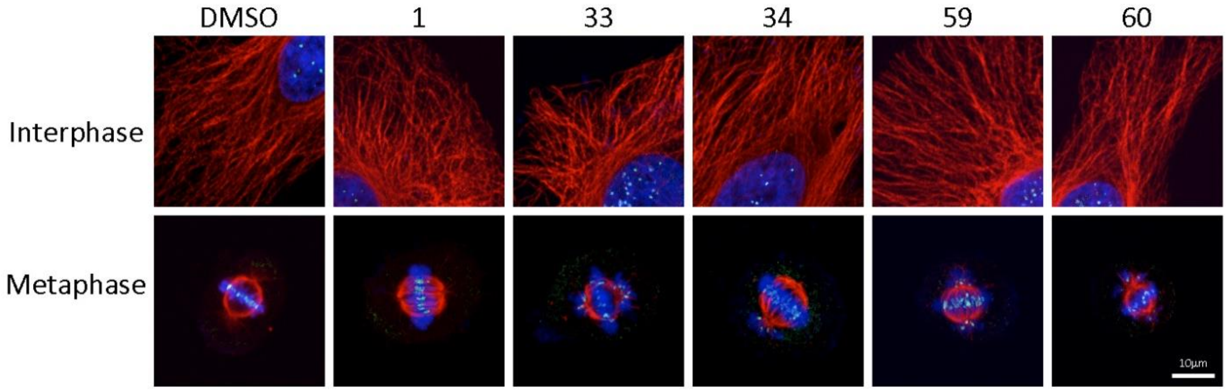

Figure S3. Microtubule organization in Hela cells treated with compounds at GI_{25}. Microtubule organization in HeLa cells during interphase and mitosis in HeLa cells treated for 4 h with compounds **1**, **33**, **34**, **59**, and **60** at each compound's GI_{25} (10nM, 7.5 nM, 225 nM, 15 nM and 20 nM respectively). Following drug treatment, cells were probed for microtubules (red), centromeres (green), and DNA (blue). Bar, 10 μm.

Cell fates of cells treated with lead compounds

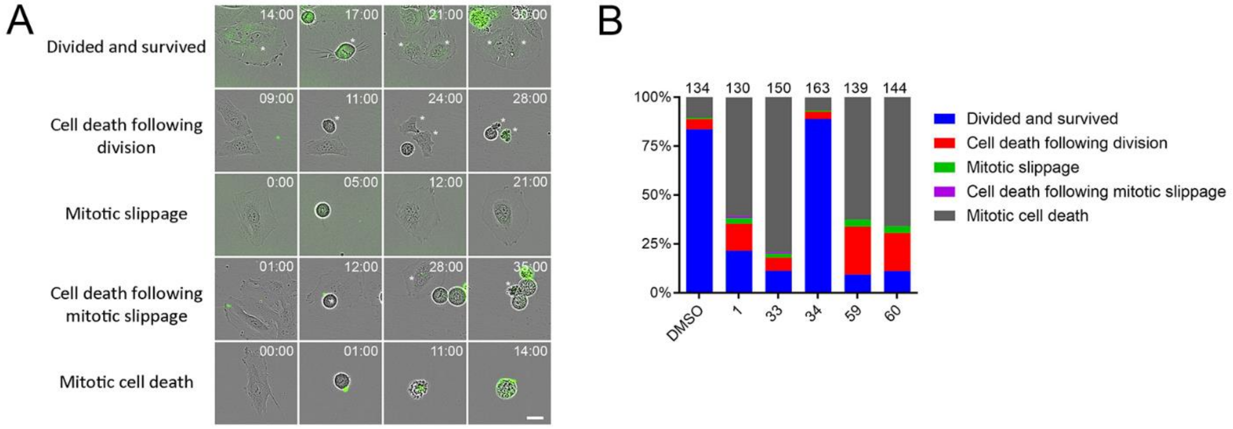

Figure S4. Representative cell fates of cells treated with lead compounds **1**, **33**, **59**, **60**. Raw image data from **Figure S2** was converted to timelapse movies (shown as overlayed phase contrast and Annexin V fluorescence [green]), and individual cells were tracked over time to determine their cell fate following mitotic arrest. Cells were binned into five possible cell fates: cells that were able to undergo cytokinesis and remained viable at least 8 hours (blue); Cells that underwent cytokinesis but had one or both daughter cells undergo apoptosis (red); cells that failed in mitosis and/or cytokinesis but slipped back out of mitosis to survive at least 8 hours (green); cells that underwent mitotic slippage only to die (magenta); and cells that died in mitosis (grey). **A)** Numbers indicate the time point for each frame within the timelapse sequence, and asterisks are placed on-or adjacent to cells to denote the cell being tracked. Bar, 20 μm. **B)** Numbers at the top of each column denotes the number of individual cells scored for each treatment.

Movie S1-HeLa cell and Control. Mitosis in a HeLa cell expressing EGFP-tubulin (green) and cultured in the presence of 0.1% DMSO and NucSpot Live 650 (magenta) Image stacks (10 planes at 1.36 mm intervals) were acquired every 2 minutes, beginning at prophase, and played back at 8 frames/sec. See separate downloadable file.

Movie S2- HeLa cell and Compound 33. Prometaphase arrest and mitotic slippage in a HeLa cell expressing EGFP-tubulin (green) and cultured in the presence of 7.5 nM compound **33** and NucSpot Live 650 (magenta) Image stacks (10 planes at 1.36 mm intervals) were acquired every 2 minutes, beginning at prophase, and played back at 20 frames/sec. See separate downloadable file.

Table S1. Selected Cytotoxicity GI_{50}s of compounds against HeLa, PANC1, and PNT1A cells.

Selected compounds were additionally tested for cytotoxicity against PANC1 and PNT1A cells using the MTT assay. The therapeutic index as an indication of toxicity, is also calculated.

Compound Number	HeLa GI_{50} (mM)	PANC1 GI_{50} mM)	PNT1A GI_{50}(mM)	Therapeutic index
**1**	0.022 ± 0.002		0.036 ± 0.009	1.6
**13**	0.142 ± 0.044			
**14**	2.30 ± 0.09			
**15**	11.3 ± 0.65			
**21**	0.062 ± 0.005		0.053 ± 0.012	0.9
**27**	0.025 ± 0.002		0.049 ± 0.020	2.0
**30**	0.043 ± 0.002			
**31**	0.047 ± 0.002			
**33**	0.015 ± 0.004	0.015 ± 0.002	0.024 ± 0.005	1.6
**34**	0.444 ± 0.030			
**35**	2.865 ± 0.103			
**59**	0.016 ± 0.004		0.041 ± 0.011	2.6
**60**	0.022 ± 0.005		0.048 ± 0.010	2.2

^{1}H & ^{13}C NMR spectra of the final 7-deazahypoxanthine compounds

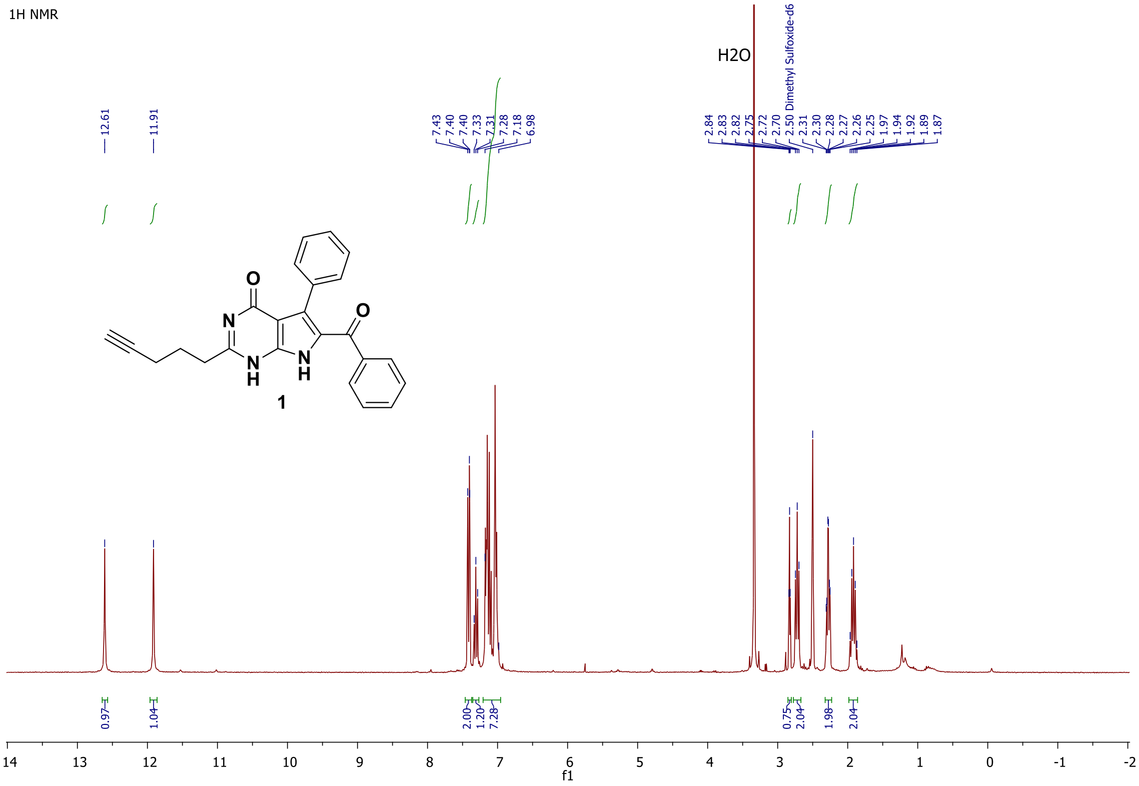

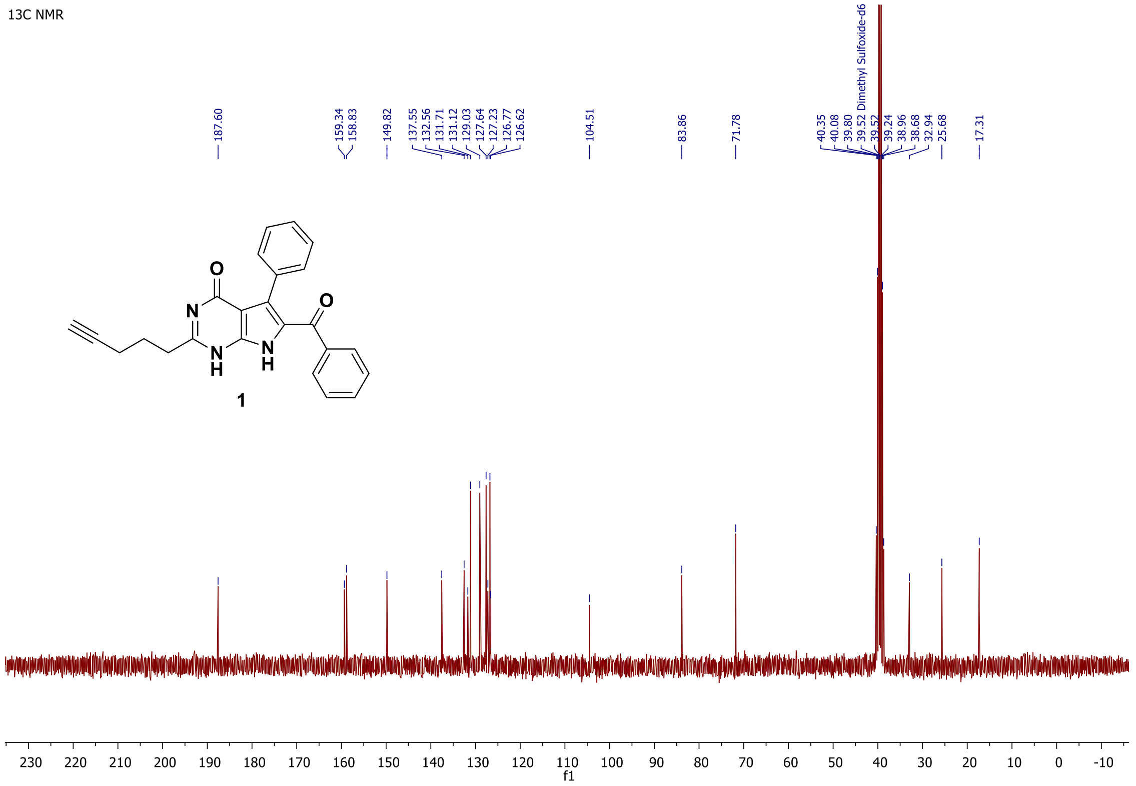

H

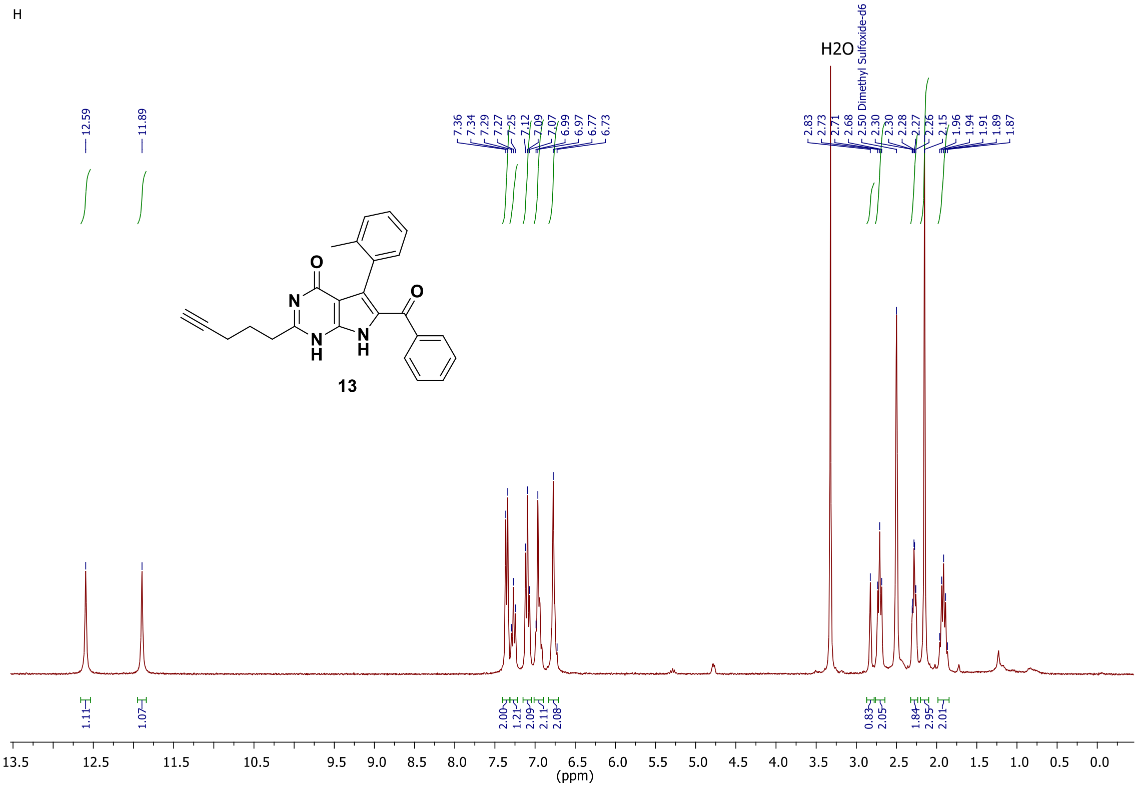
H2O
Dimethyl Sulfoxide-d6
12.59
11.89
7.36 7.34 7.29 7.27 7.25 7.12 7.09 7.07 6.99 6.97 6.77 6.73
2.83 2.73 2.71 2.68 2.50 Dimethyl Sulfoxide-d6 2.30 2.30 2.28 2.27 2.26 2.15 1.96 1.94 1.91 1.89 1.87
O
N
N
H
N
H
O
13
1.11
1.07
2.00 1.21 2.09 2.11 2.08
0.83 2.05
1.84 2.95 2.01
13.5 12.5 11.5 10.5 9.5 9.0 8.5 8.0 7.5 7.0 6.5 6.0 5.5 5.0 4.5 4.0 3.5 3.0 2.5 2.0 1.5 1.0 0.5 0.0
(ppm)

13C NMR

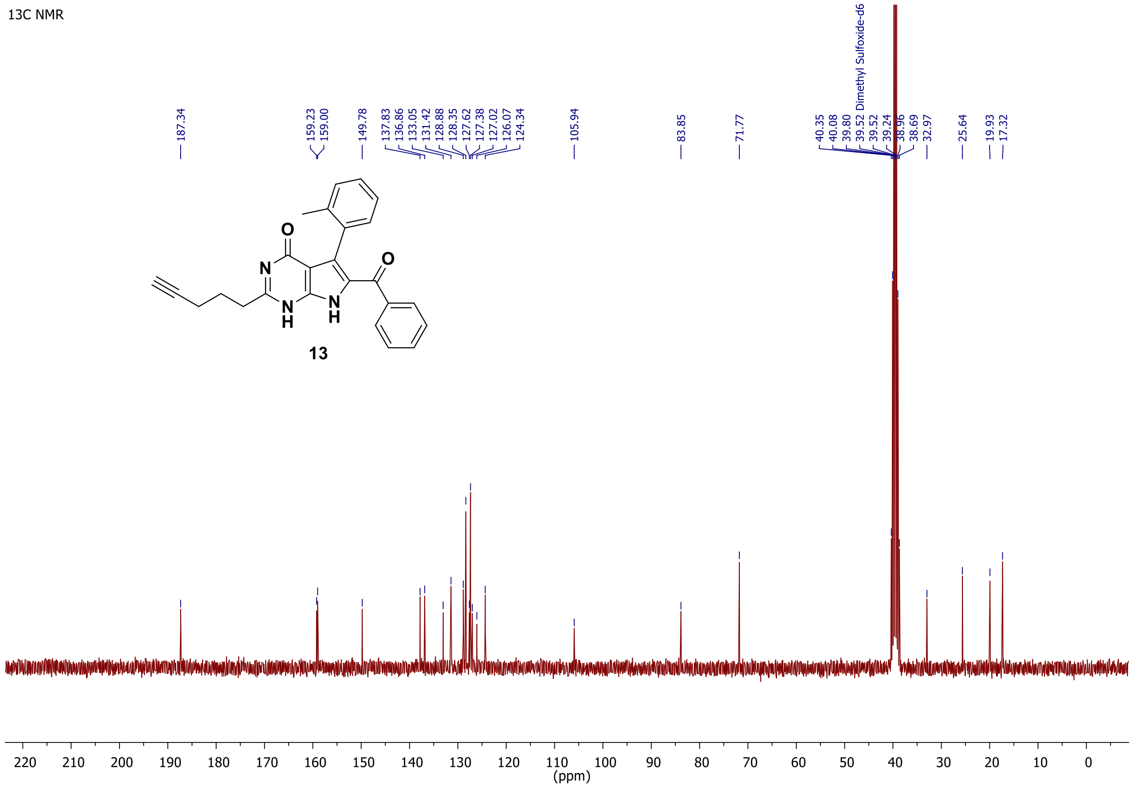
Dimethyl Sulfoxide-d6
187.34
159.23 159.00
149.78
137.83 136.86 133.05 131.42 128.88 128.35 127.62 127.38 127.02 126.07 124.34
105.94
83.85
71.77
40.35 40.08 39.80 39.52 Dimethyl Sulfoxide-d6 39.52 39.24 38.96 38.69 32.97
25.64
19.93 17.32
O
N
N
H
N
H
O
13
220 210 200 190 180 170 160 150 140 130 120 110 100 90 80 70 60 50 40 30 20 10 0
(ppm)

1H NMR

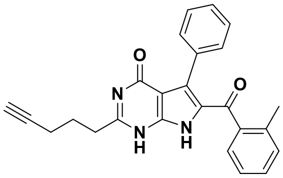

21

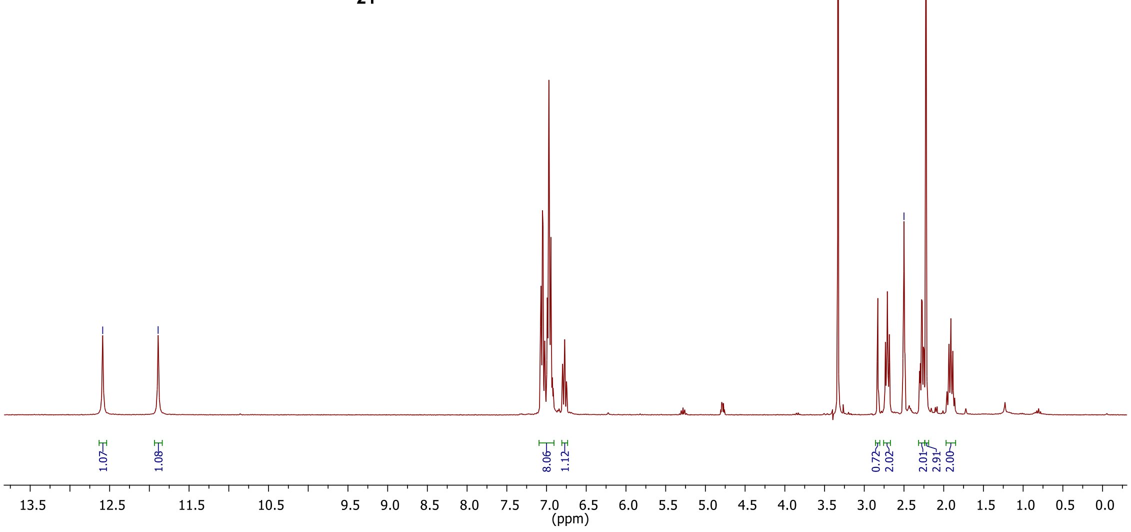

13C NMR

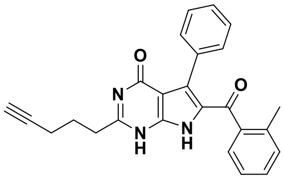

21

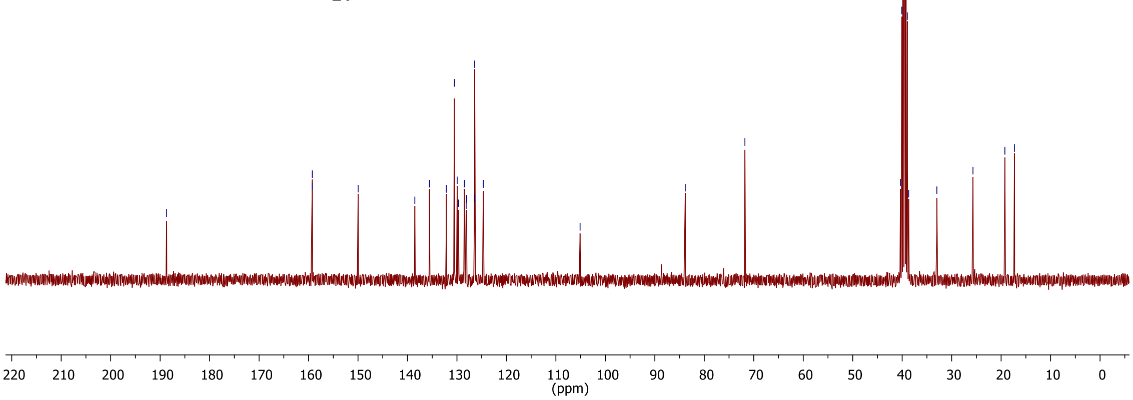

1H NMR

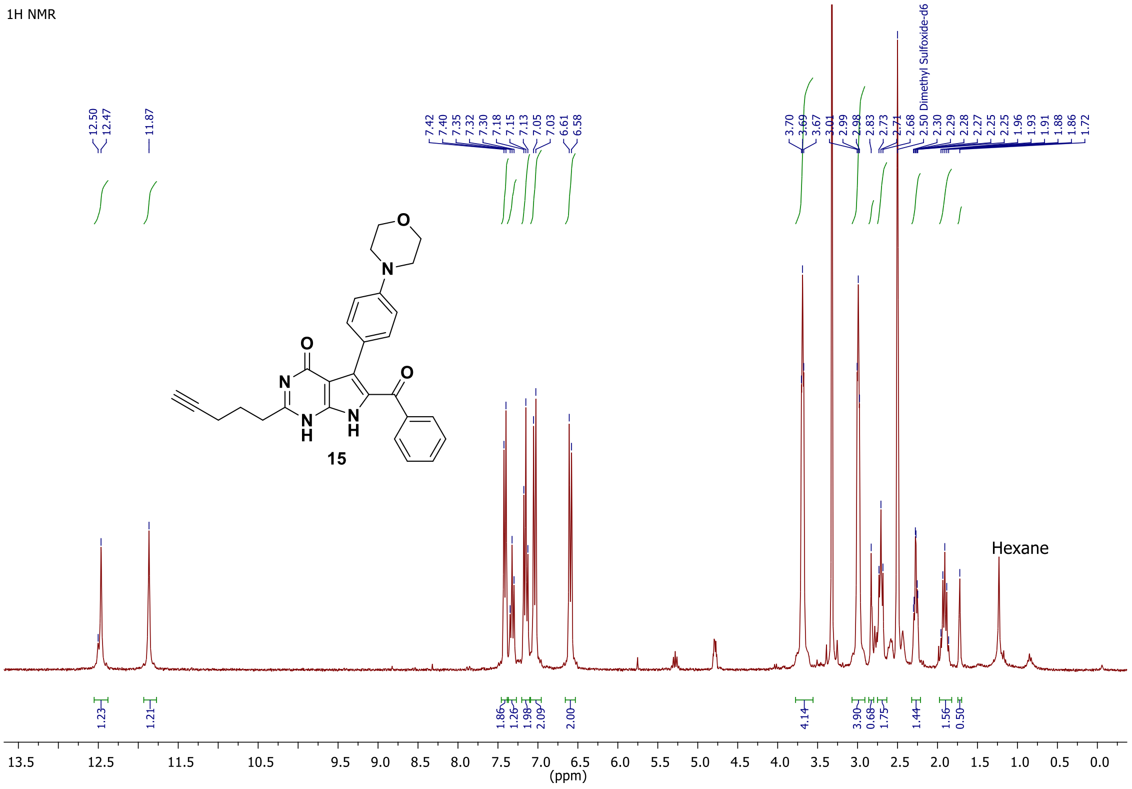

15

13C NMR

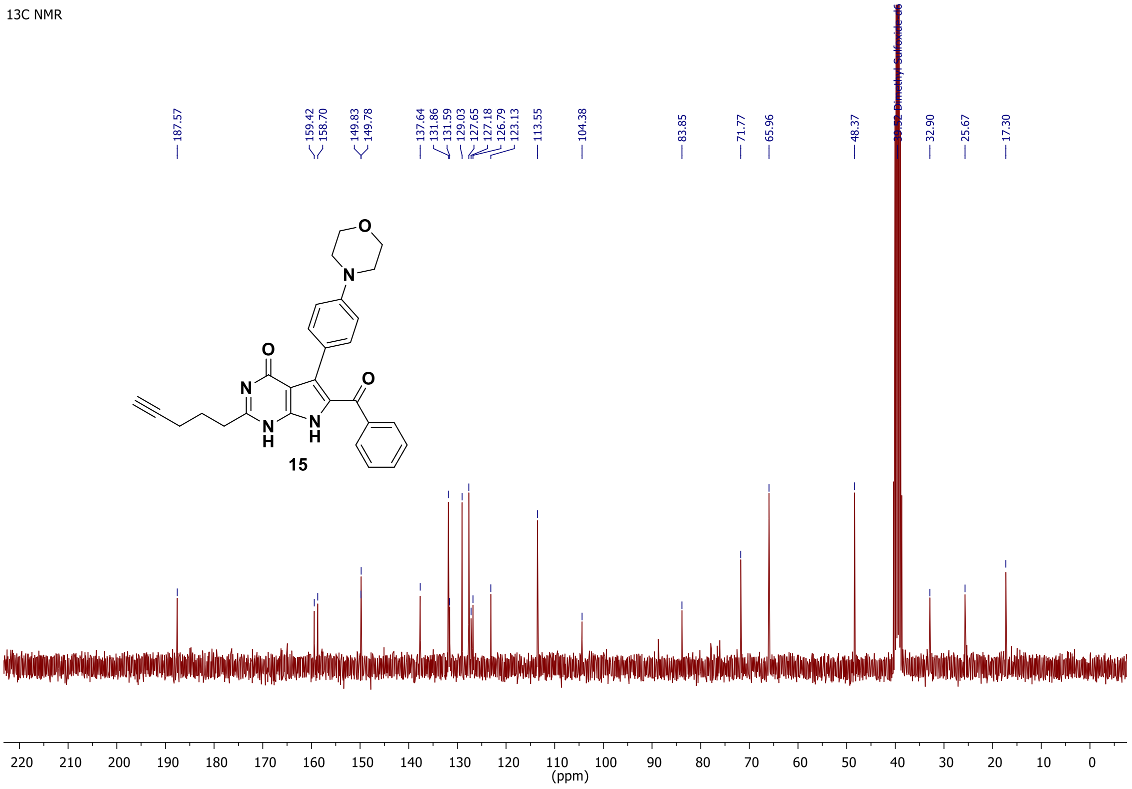

15

1H NMR

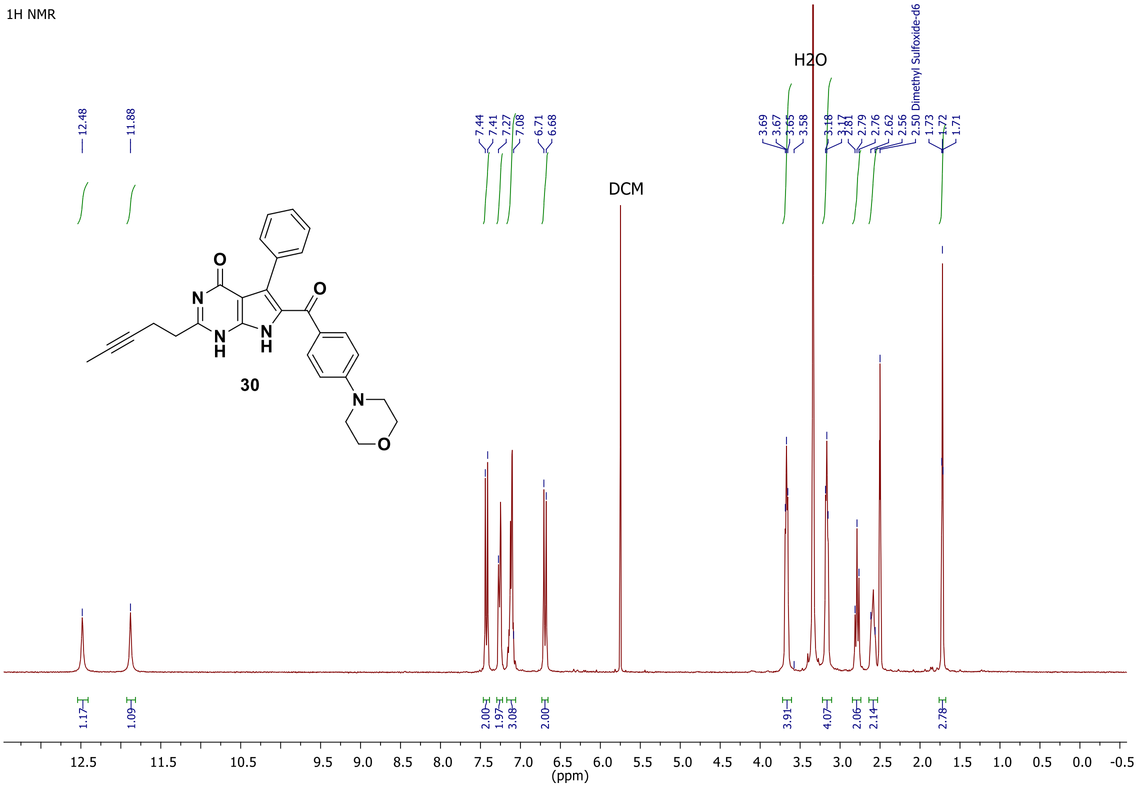

13C NMR

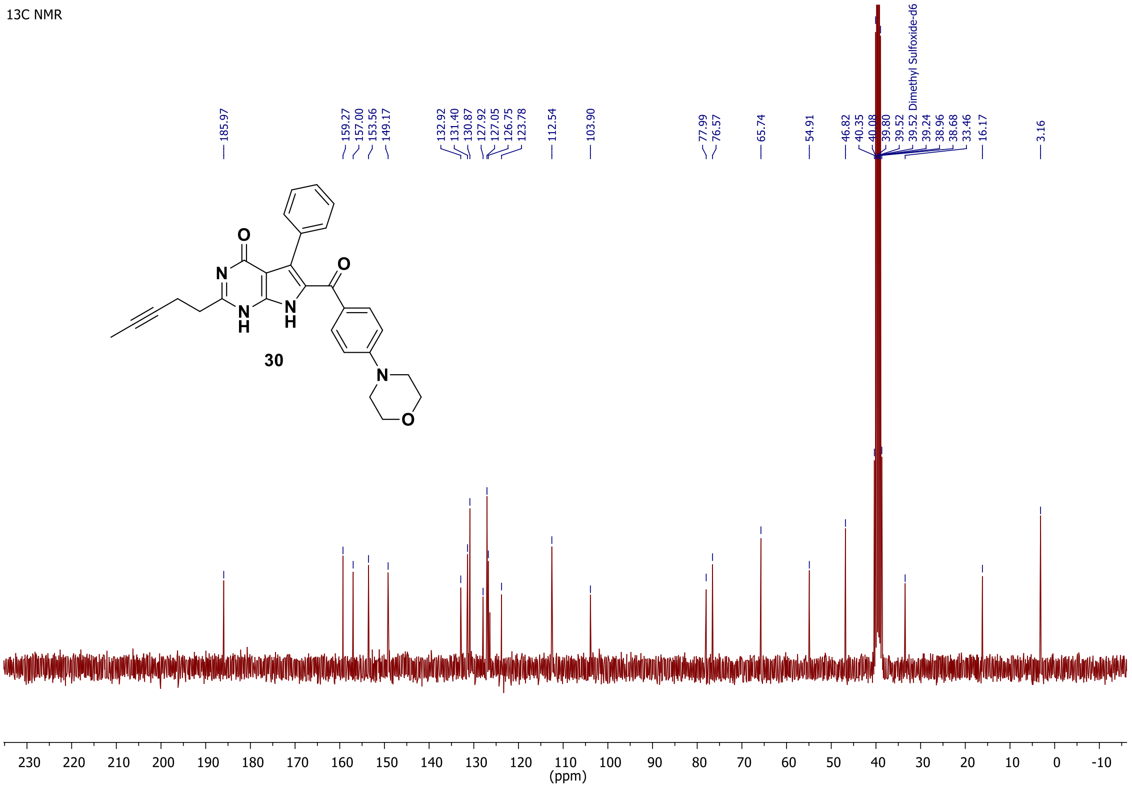

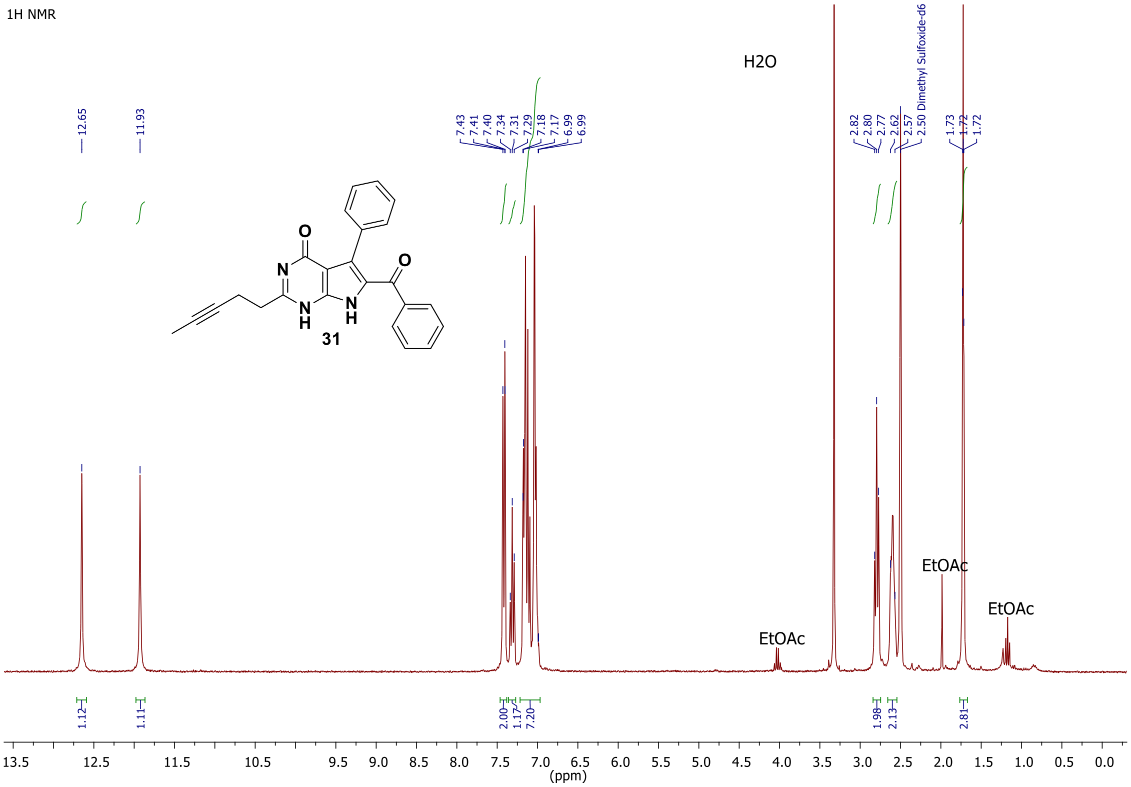
1H NMR
H2O
12.65
11.93
7.43
7.41
7.40
7.34
7.31
7.29
7.18
7.17
6.99
6.99
2.82
2.80
2.77
2.62
2.57
2.50 Dimethyl Sulfoxide-d6
1.73
1.72
1.72
O
N
N
H
N
H
O
31
EtOAc
EtOAc
EtOAc
1.12
1.11
2.00
1.17
7.20
1.98
2.13
2.81
13.5
12.5
11.5
10.5
9.5
9.0
8.5
8.0
7.5
7.0
6.5
6.0
5.5
5.0
4.5
4.0
3.5
3.0
2.5
2.0
1.5
1.0
0.5
0.0
(ppm)

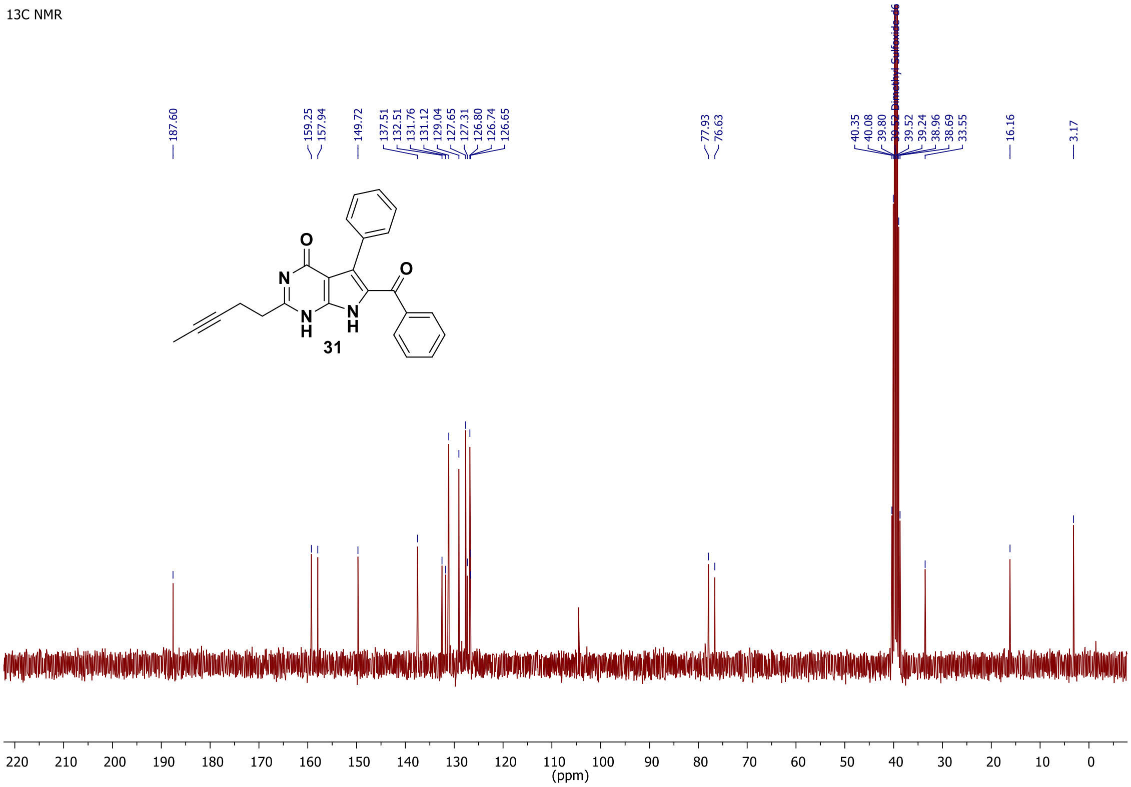
13C NMR
187.60
159.25
157.94
149.72
137.51
132.51
131.76
131.12
129.04
127.65
127.31
126.80
126.74
126.65
77.93
76.63
40.35
40.08
39.80
39.52 Dimethyl Sulfoxide-d6
39.52
39.24
38.96
38.69
33.55
16.16
3.17
O
N
N
H
N
H
O
31
220
210
200
190
180
170
160
150
140
130
120
110
100
90
80
70
60
50
40
30
20
10
0
(ppm)

1H NMR

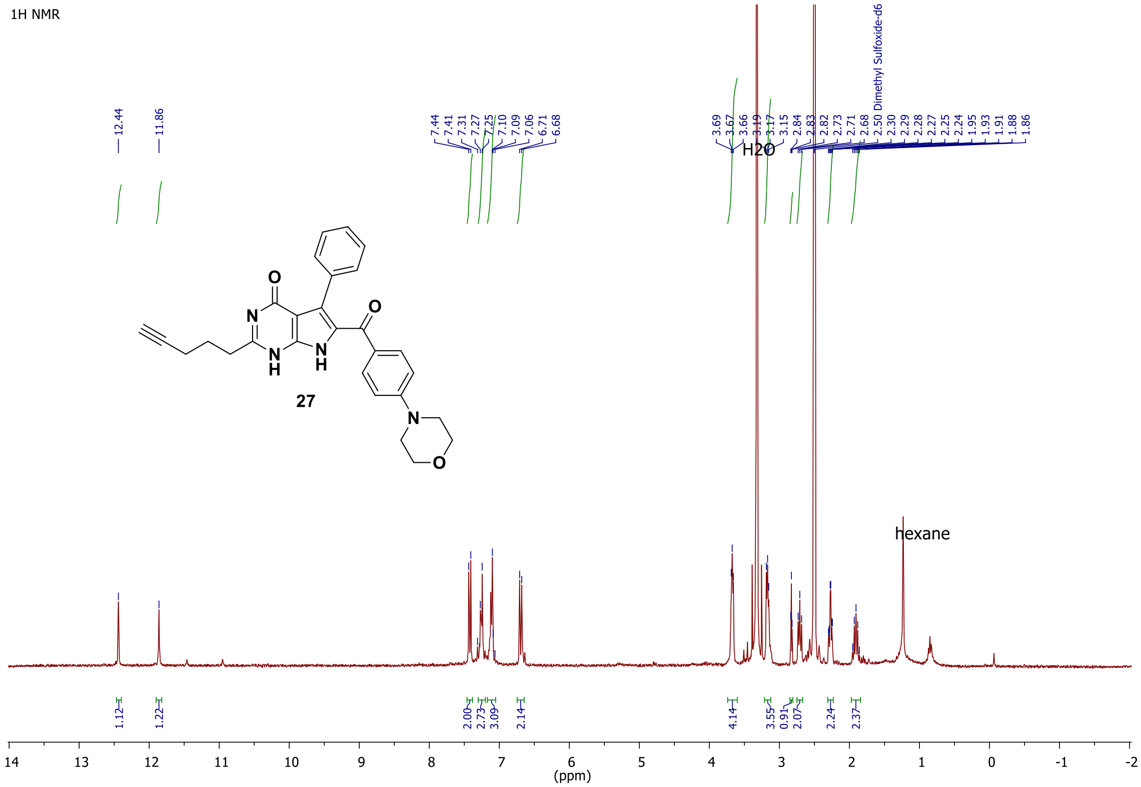

13C NMR

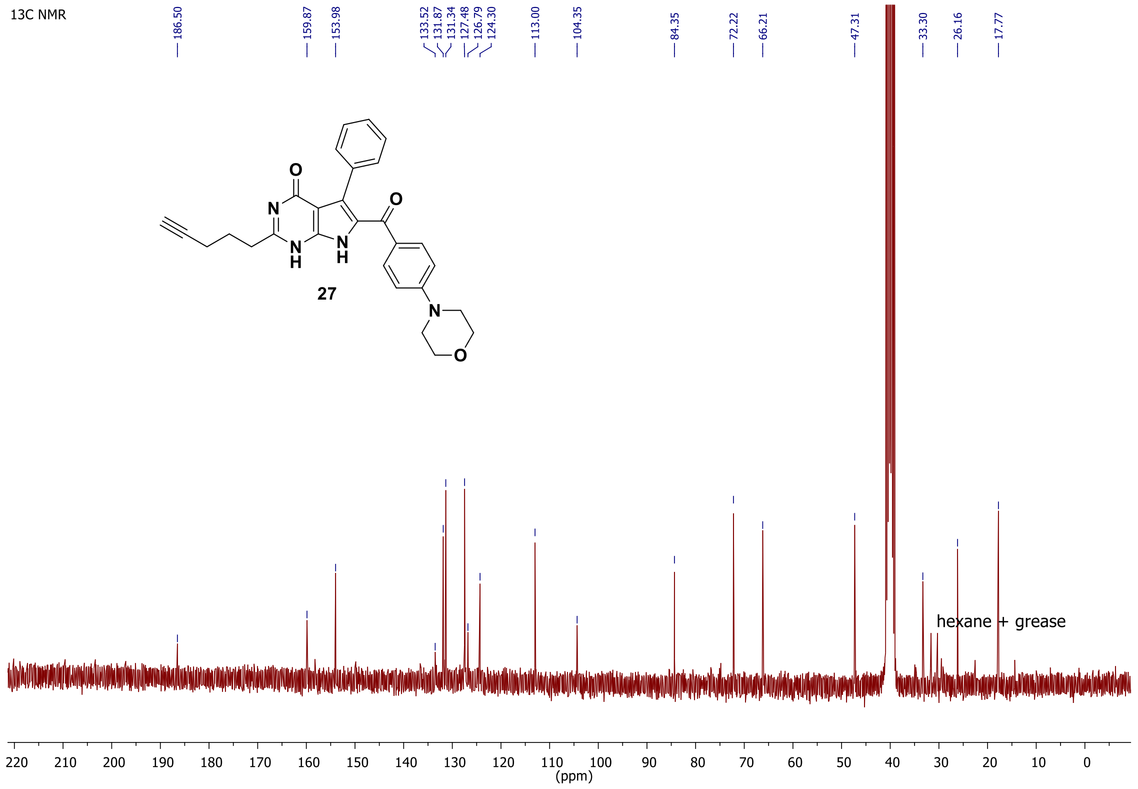

1H NMR

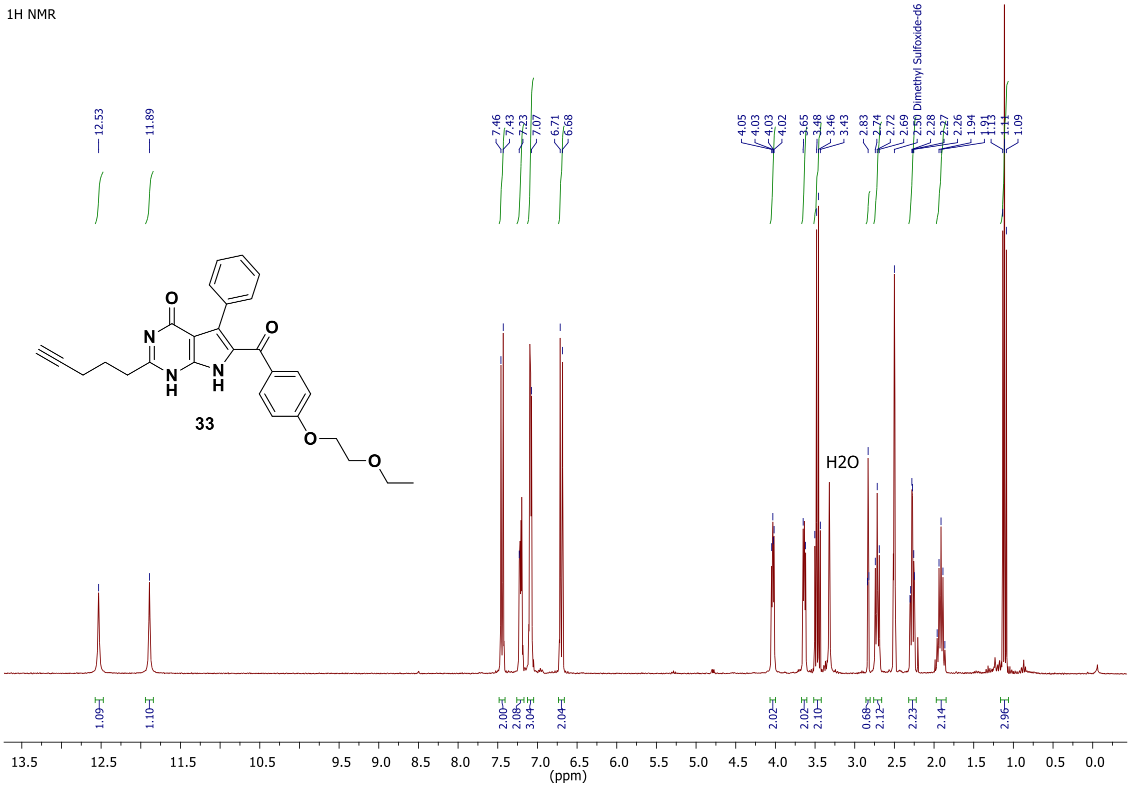

13C NMR

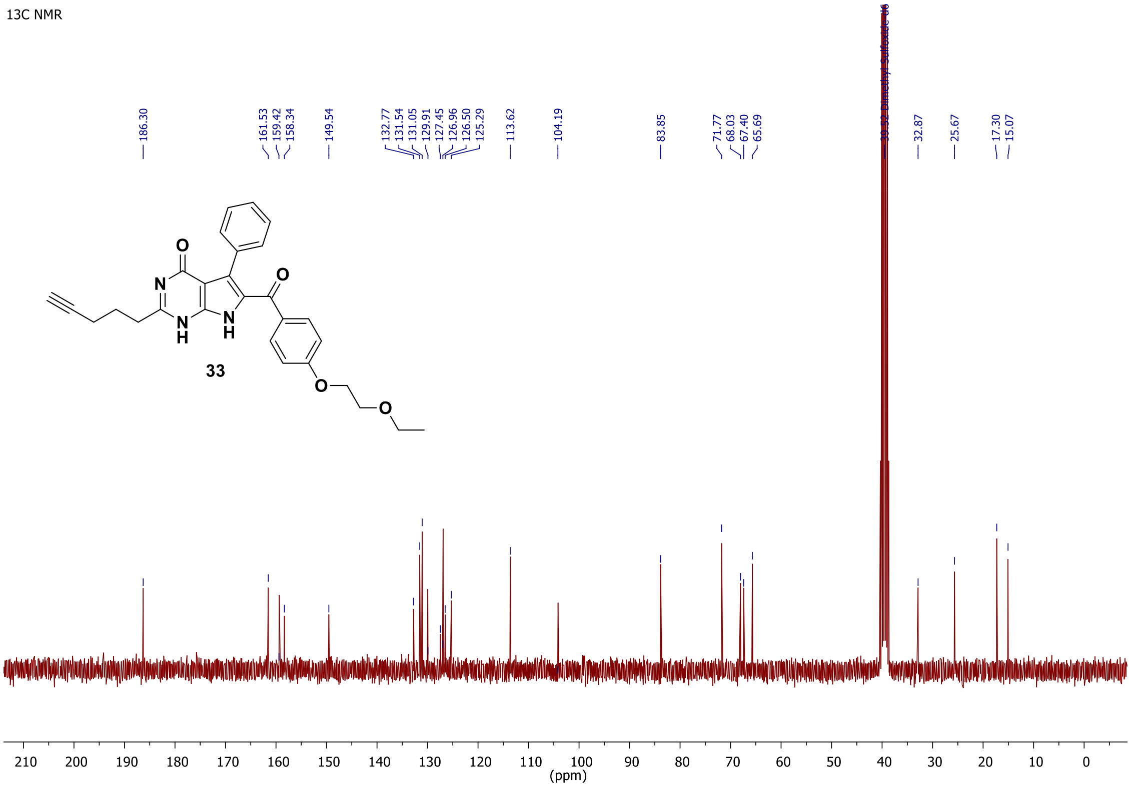

1H NMR

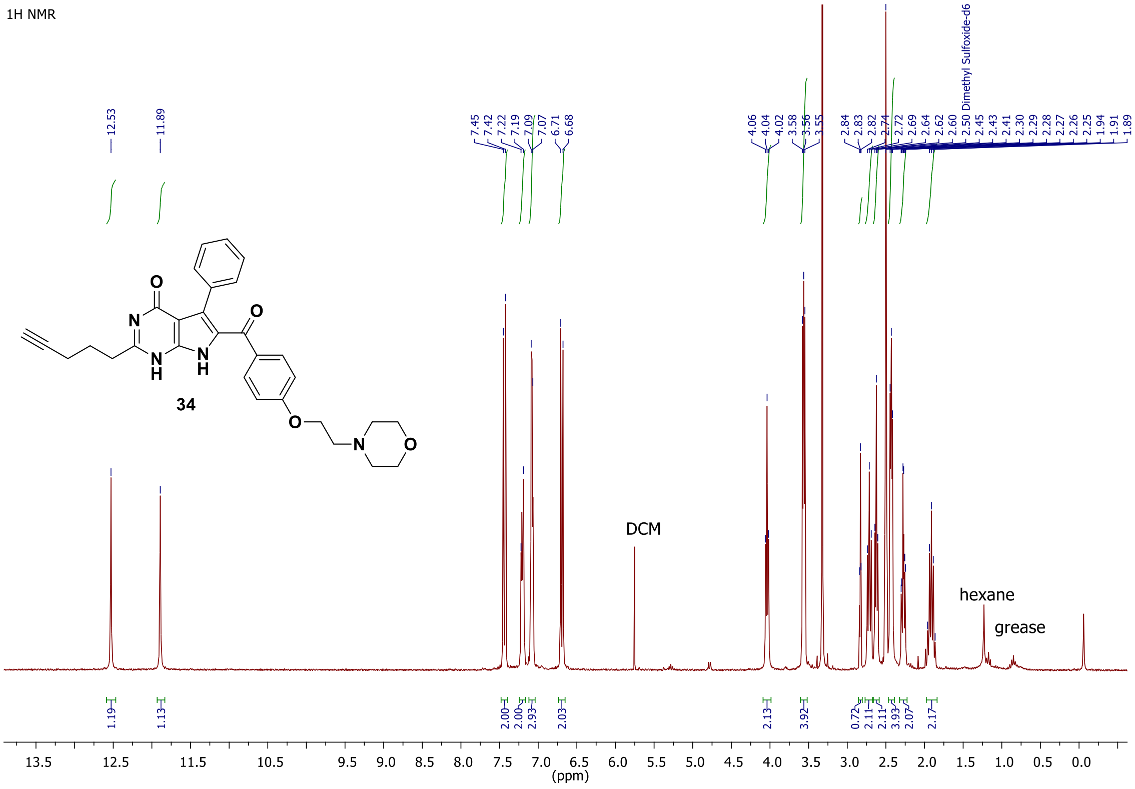

13C NMR

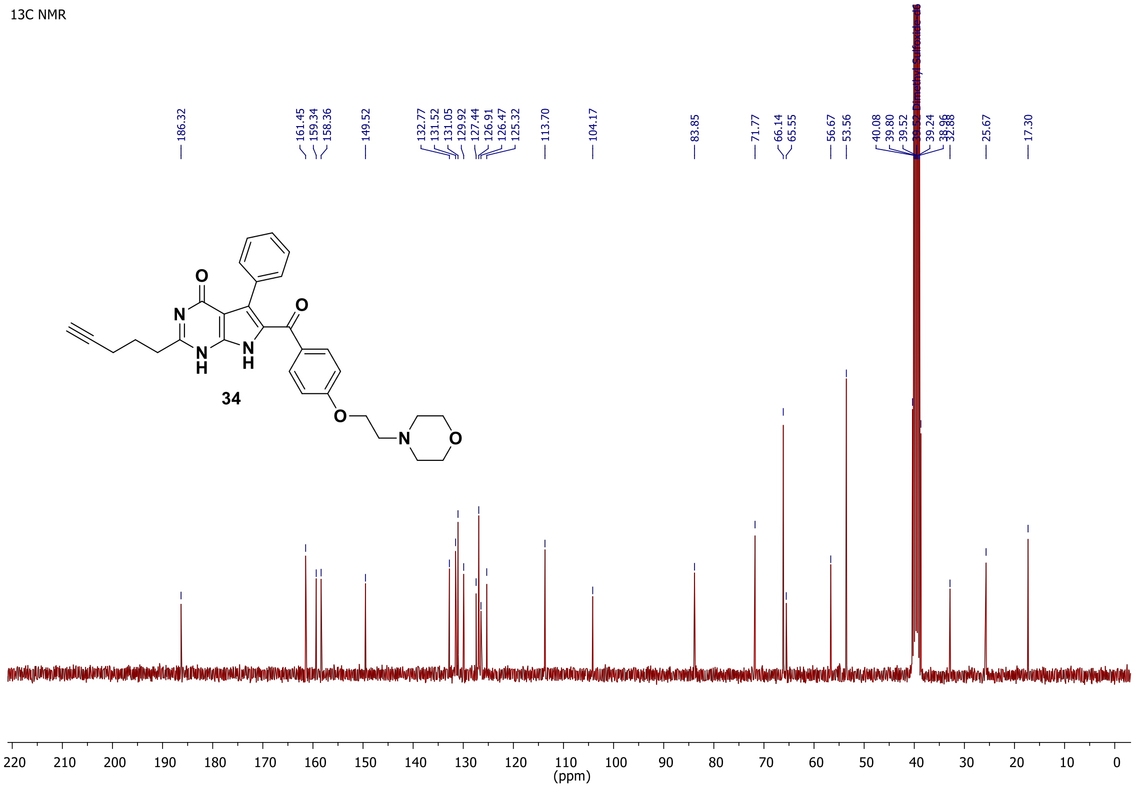

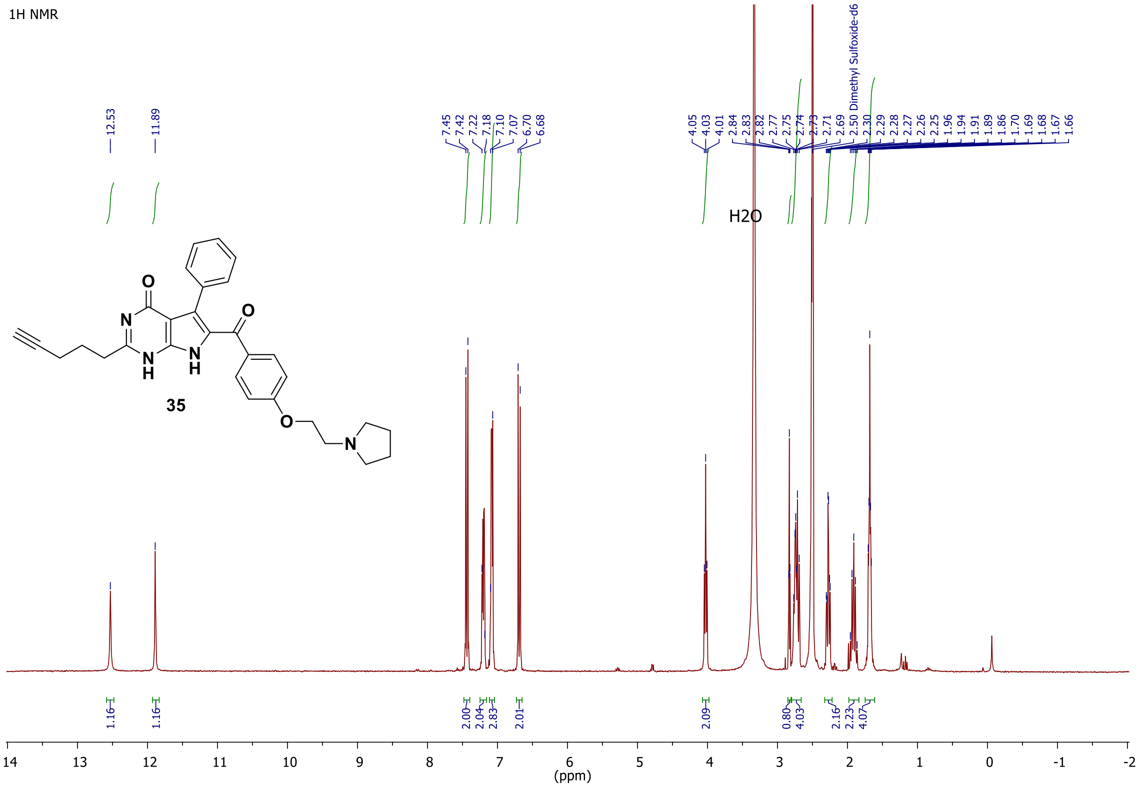
1H NMR
12.53
11.89
7.45 7.42 7.22 7.18 7.10 7.07 6.70 6.68
4.05 4.03 4.01
2.84 2.83 2.82 2.77 2.75 2.74 2.73 2.71 2.69 2.50 Dimethyl Sulfoxide-d6 2.30 2.29 2.28 2.27 2.26 2.25 1.96 1.94 1.91 1.89 1.86 1.70 1.69 1.68 1.67 1.66
H2O
O
N
N
H
H
O
O
N
35
1.16 1.16 2.00 2.04 2.83 2.01 2.09 0.80 4.03 2.16 2.23 4.07
14 13 12 11 10 9 8 7 6 5 4 3 2 1 0 -1 -2
(ppm)

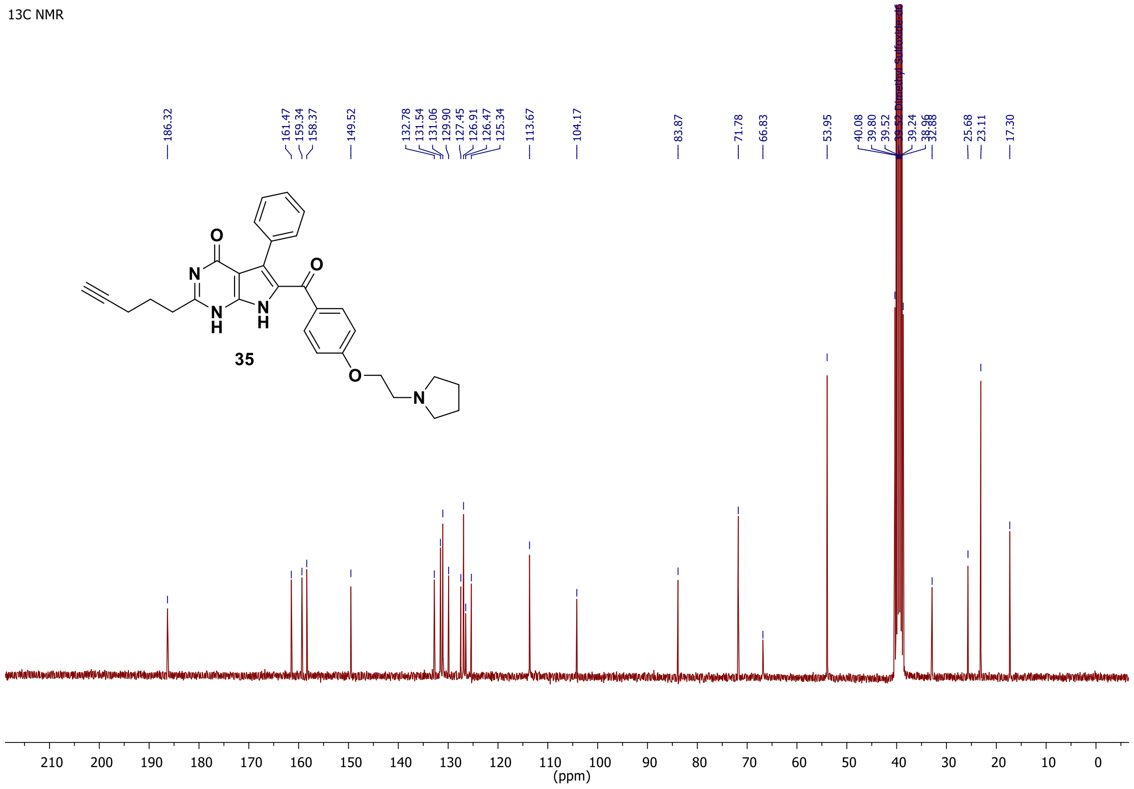
13C NMR
186.32
161.47 159.34 158.37
149.52
132.78 131.54 131.06 129.90 127.45 126.91 126.47 125.34
113.67
104.17
83.87
71.78
66.83
53.95
40.08 39.80 39.52 Dimethyl Sulfoxide-d6 39.24 38.96 32.88
25.68 23.11 17.30
O
N
N
H
H
O
O
N
35
210 200 190 180 170 160 150 140 130 120 110 100 90 80 70 60 50 40 30 20 10 0
(ppm)

1H NMR

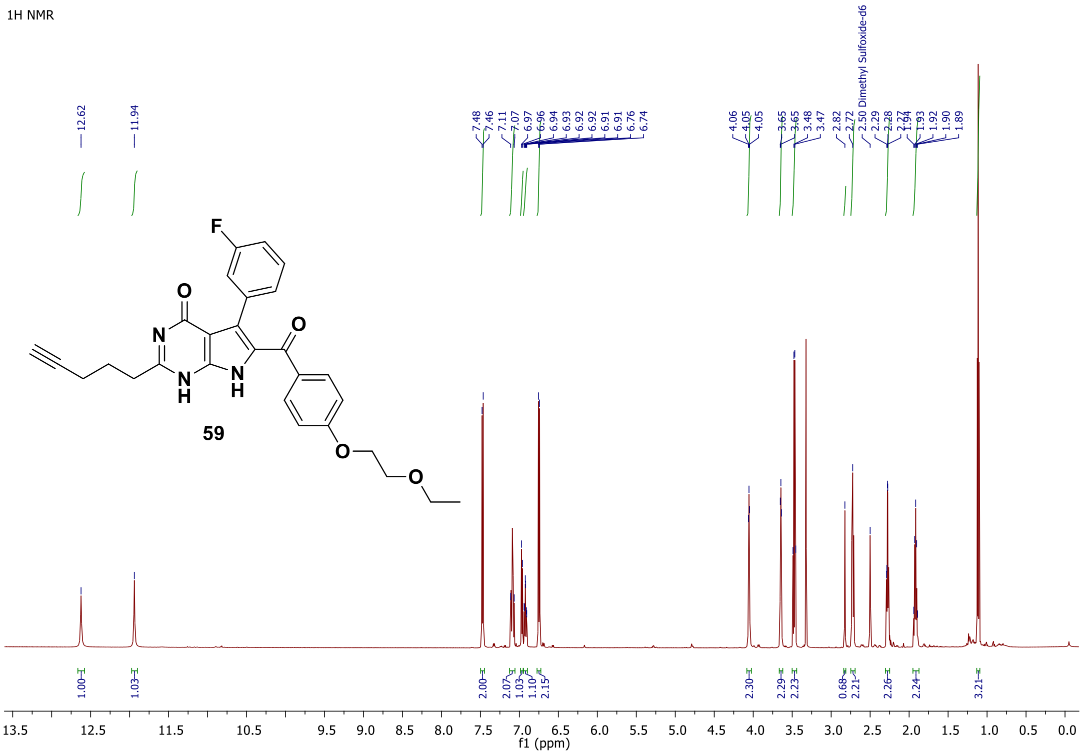

13C NMR

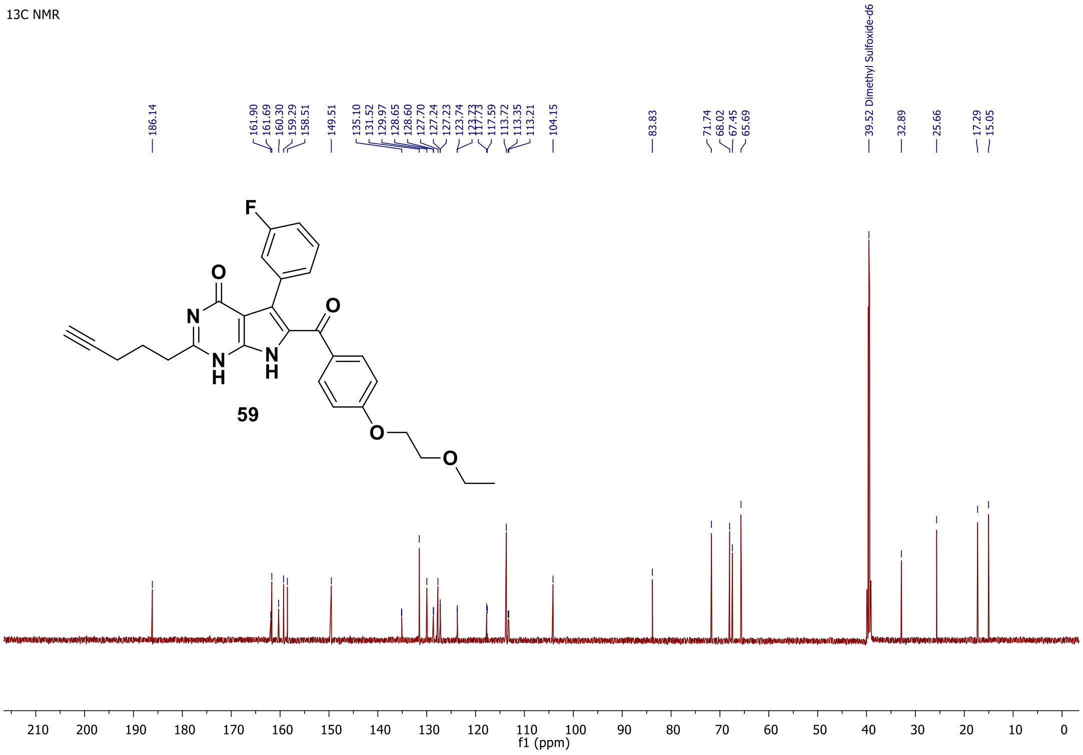

1H NMR

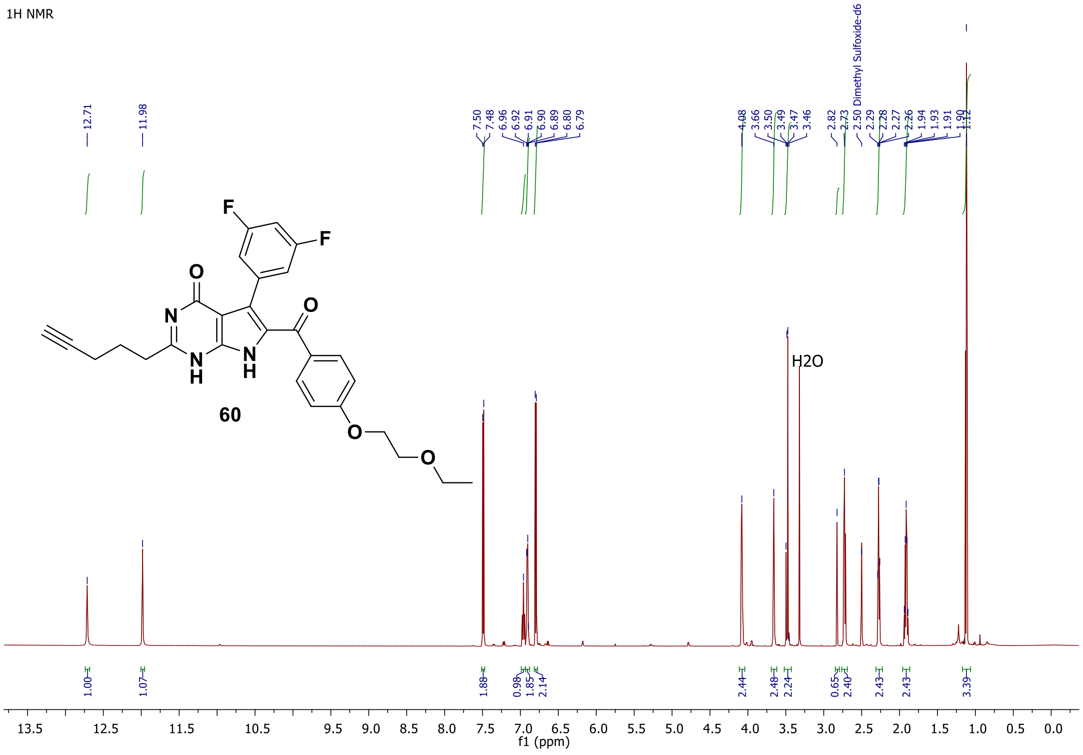

13C NMR

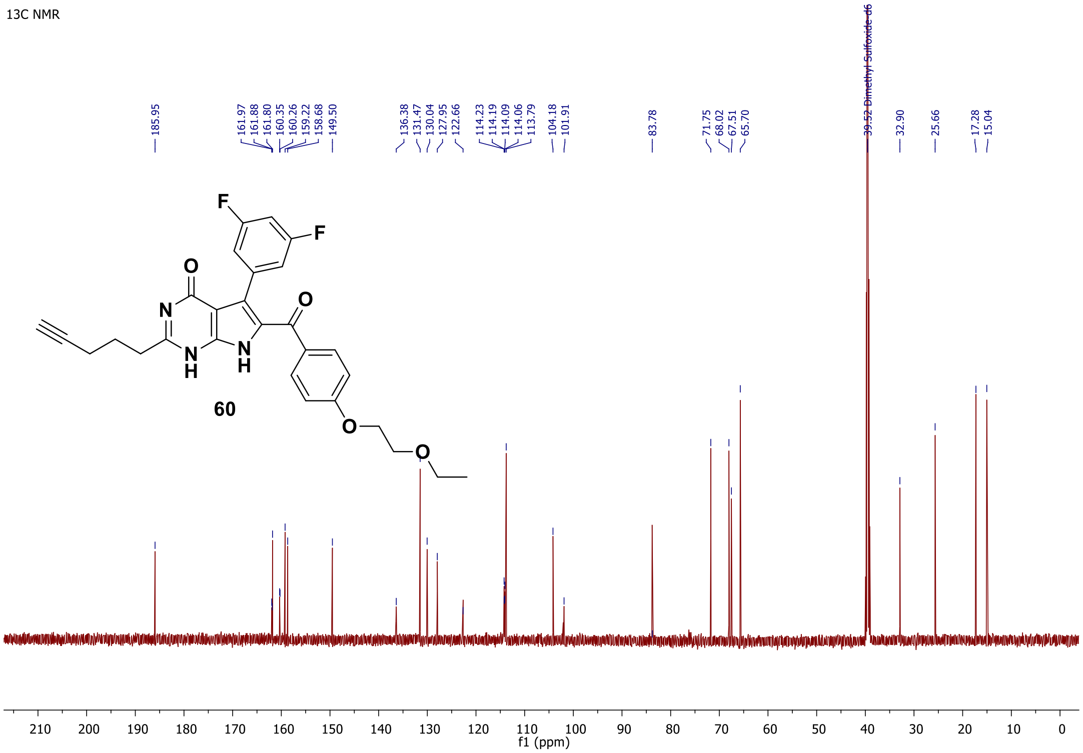

1H NMR

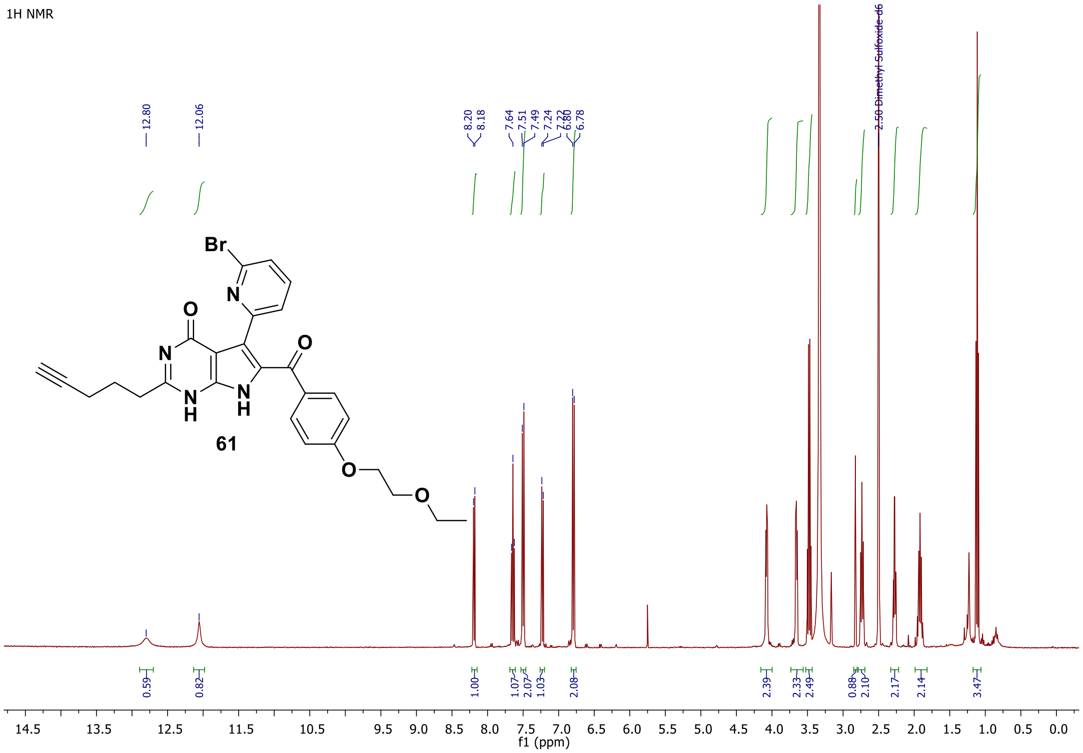

13C NMR

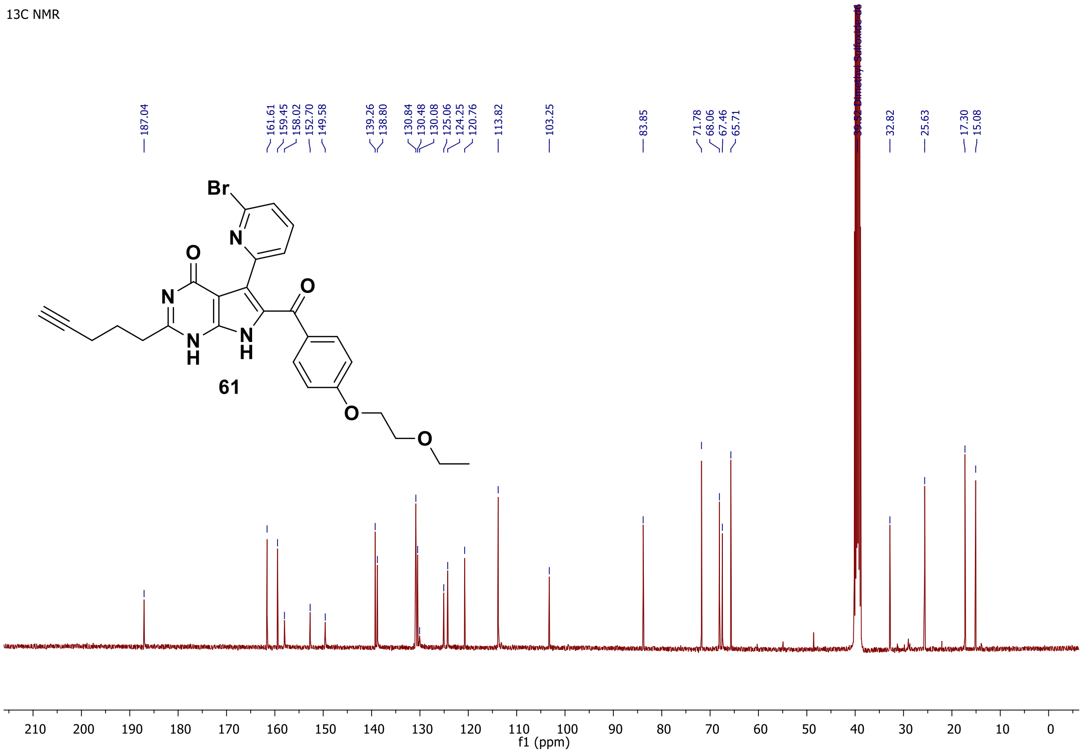

LCMS purity analysis of the final 7-deazahypoxanthine compounds

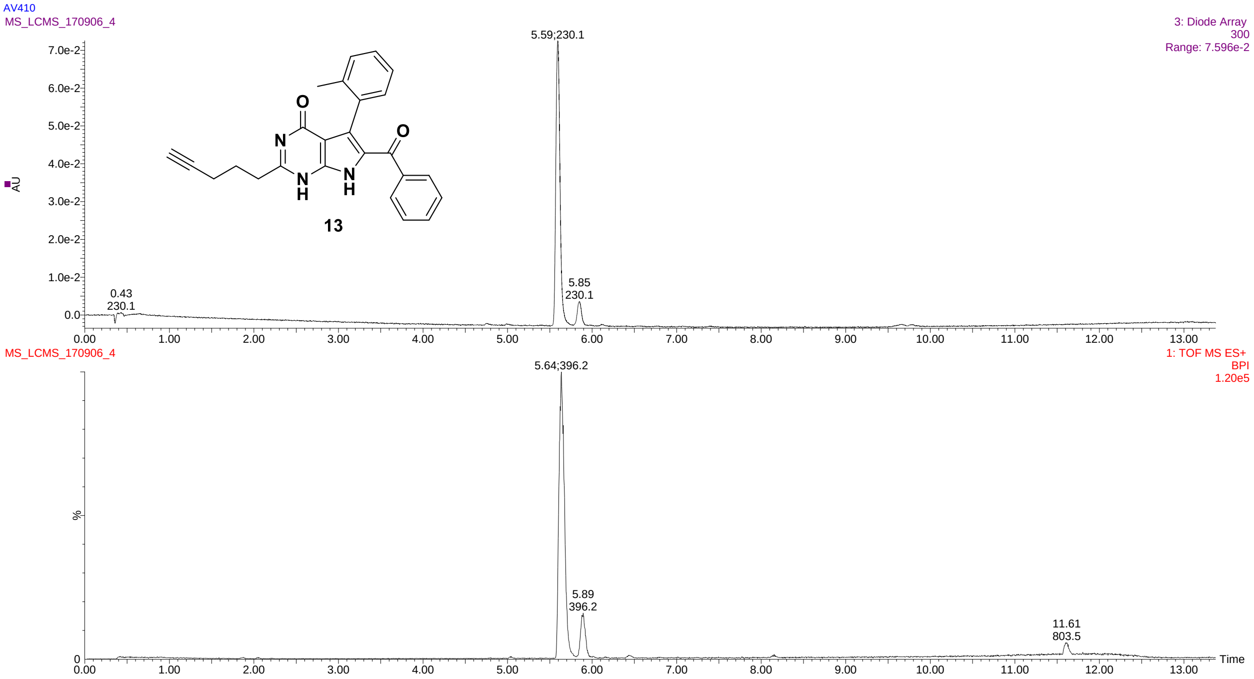

Purity: 93.5%

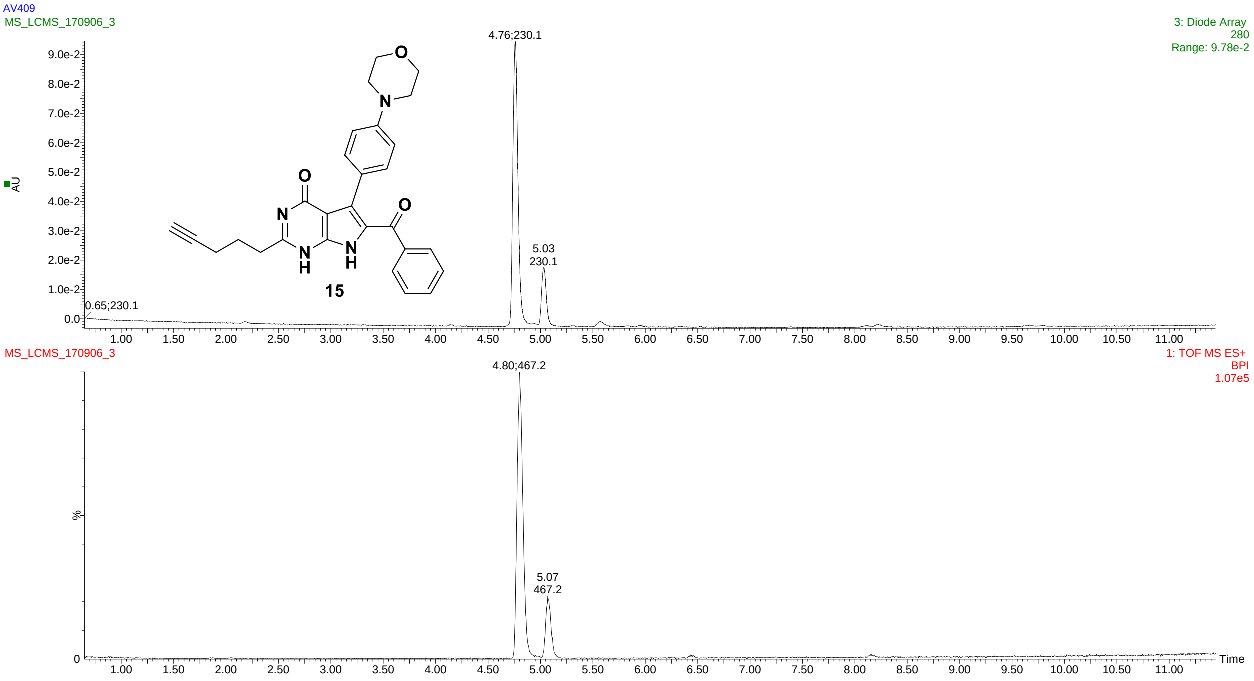

Purity: 84%

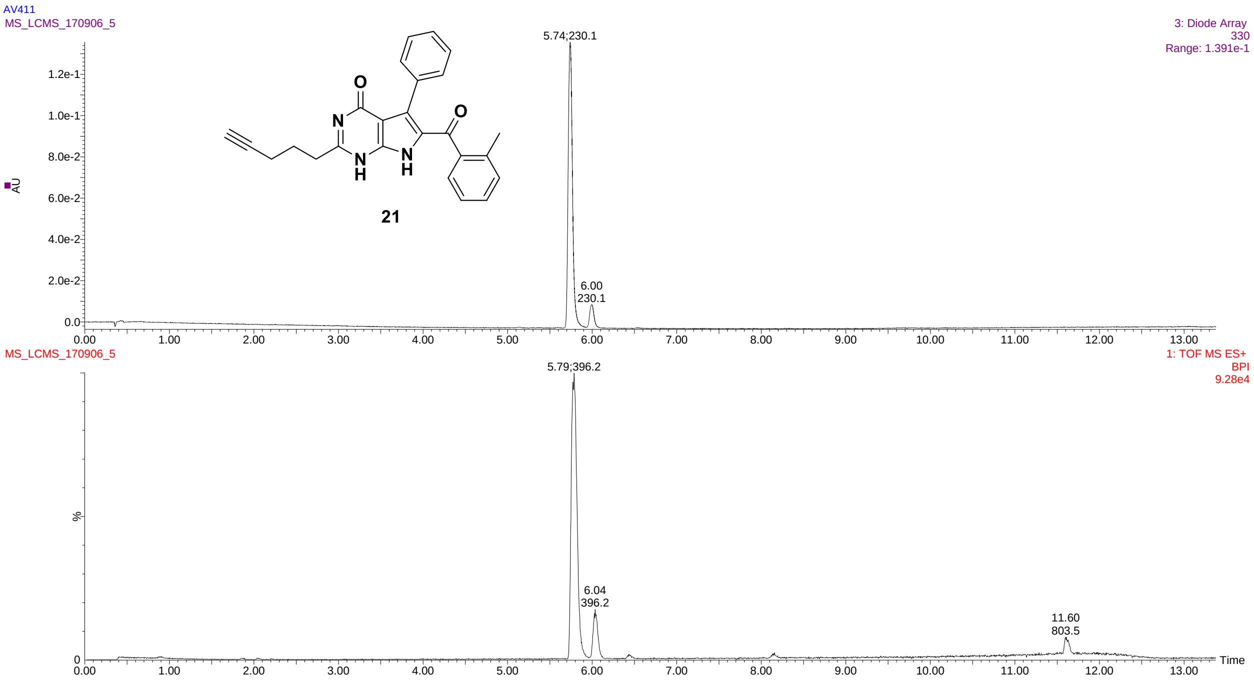

Purity: 92.7%

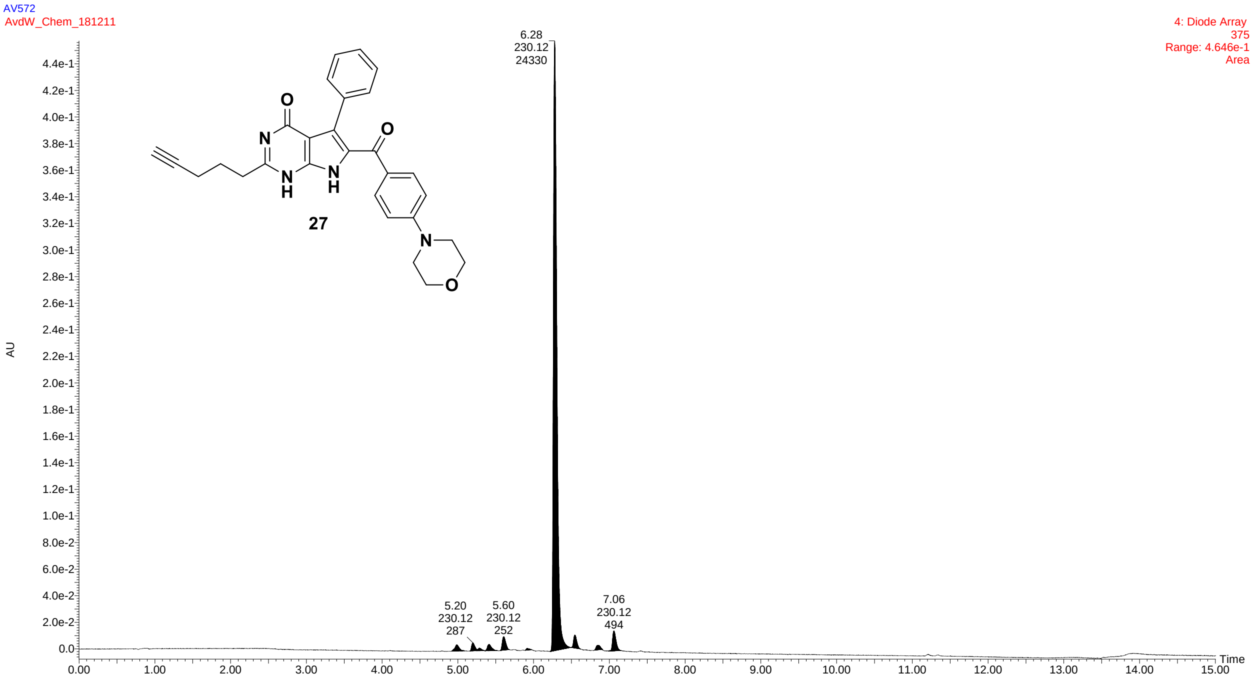

Purity: 90.5%

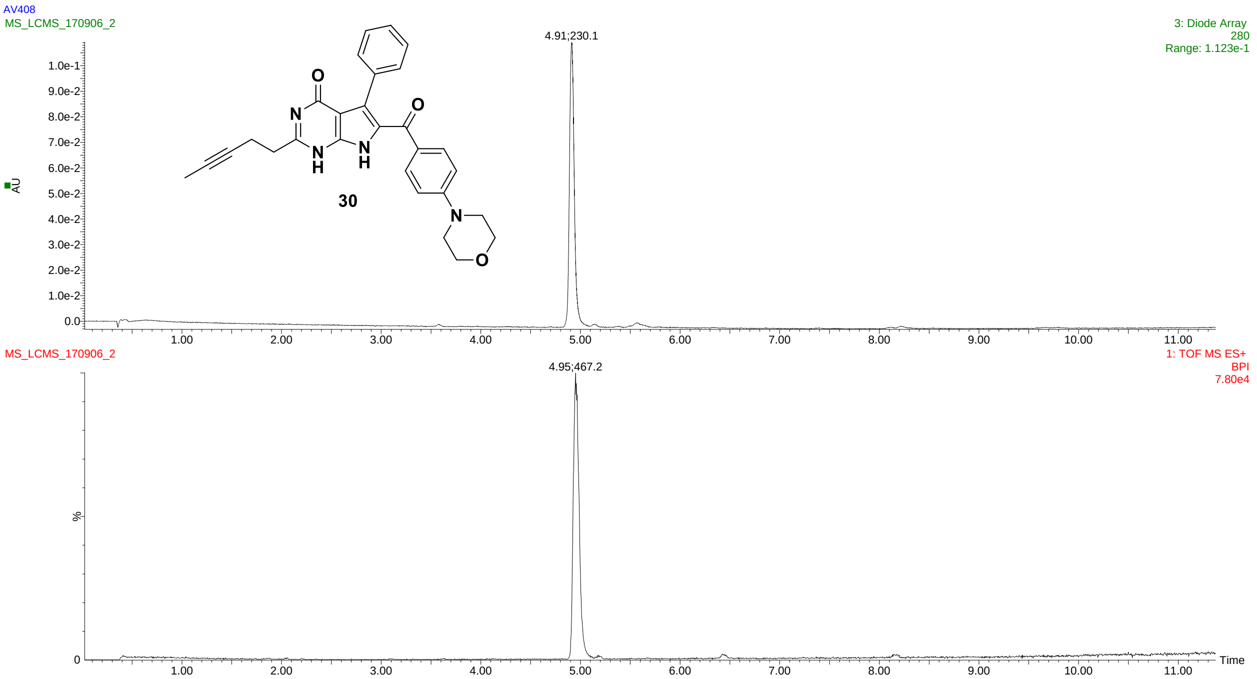

Purity: 99%

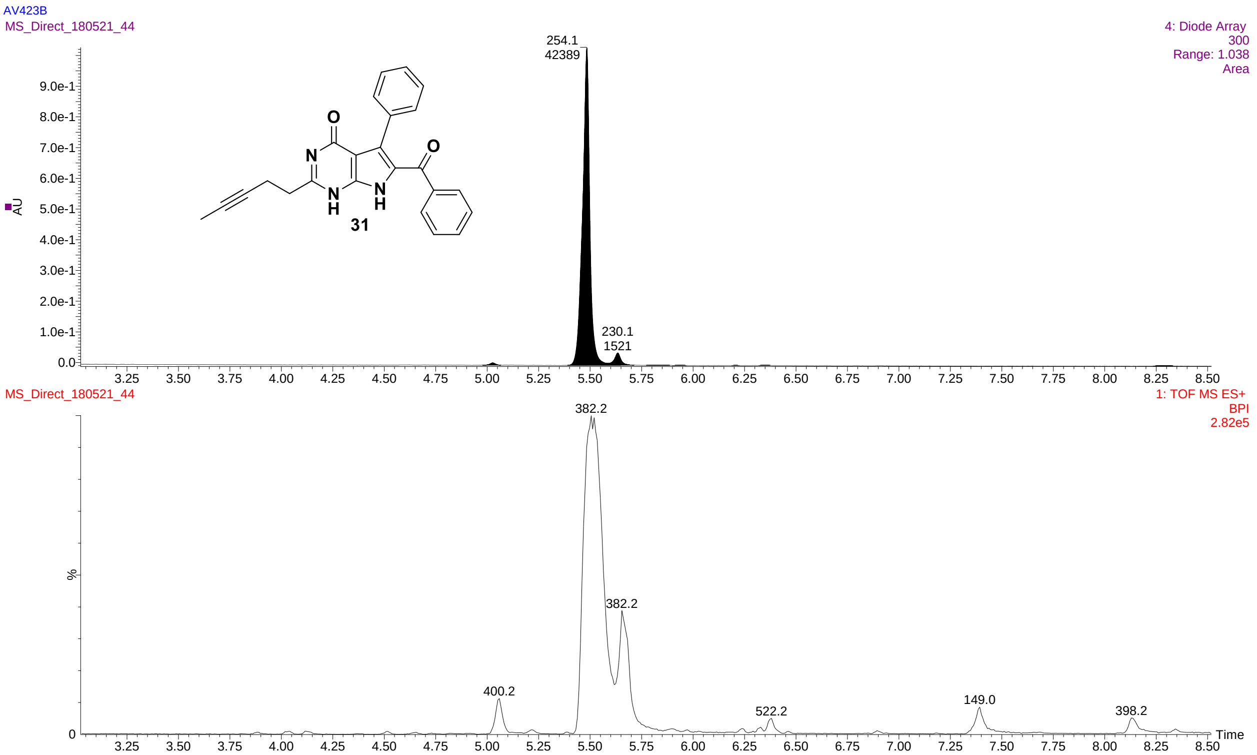

Purity: 95.4%

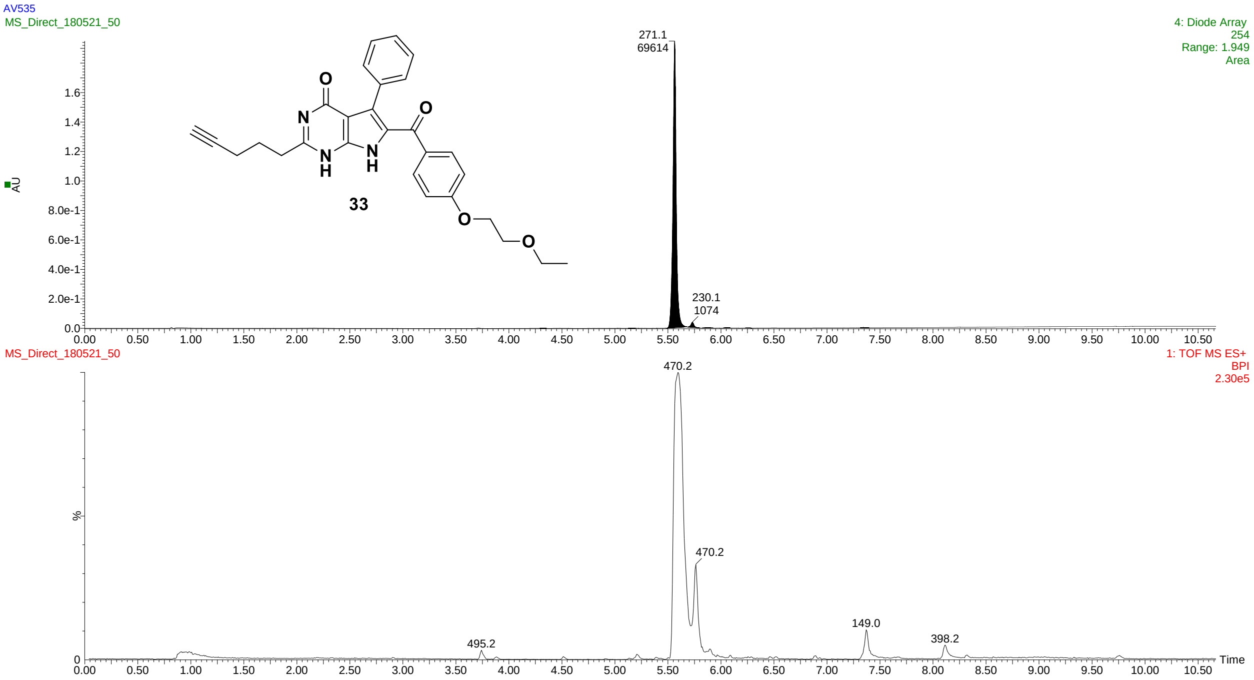

Purity: 98%

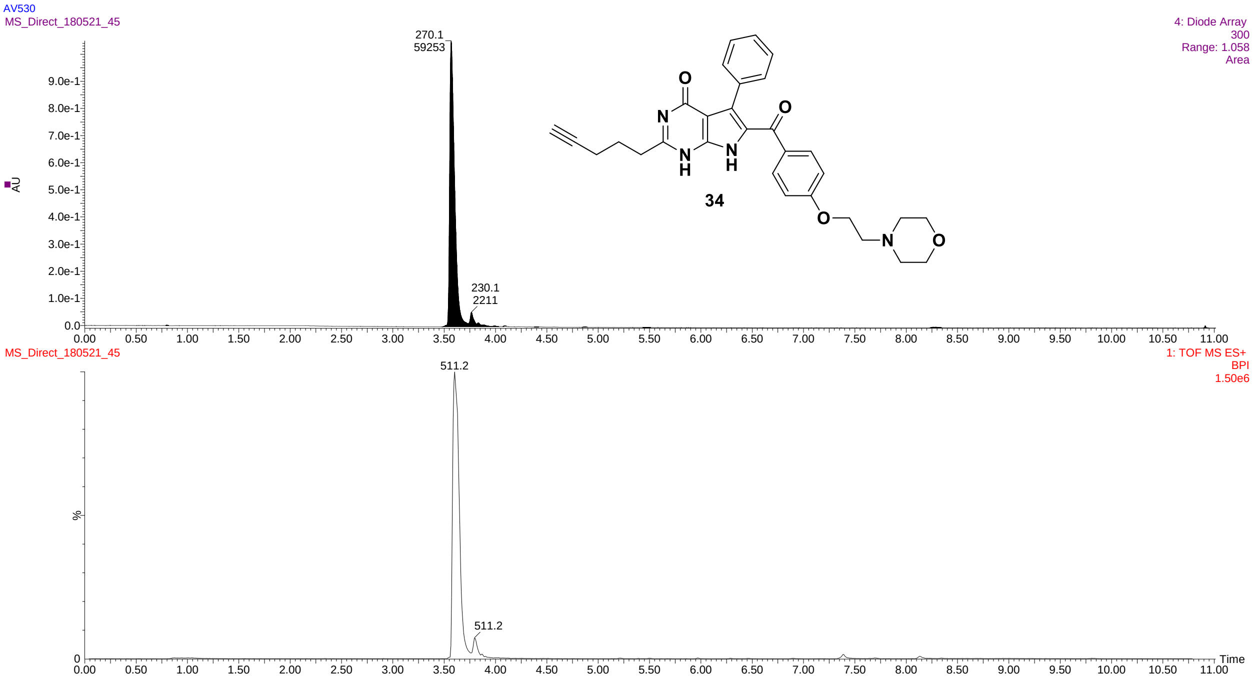

Purity: 95%

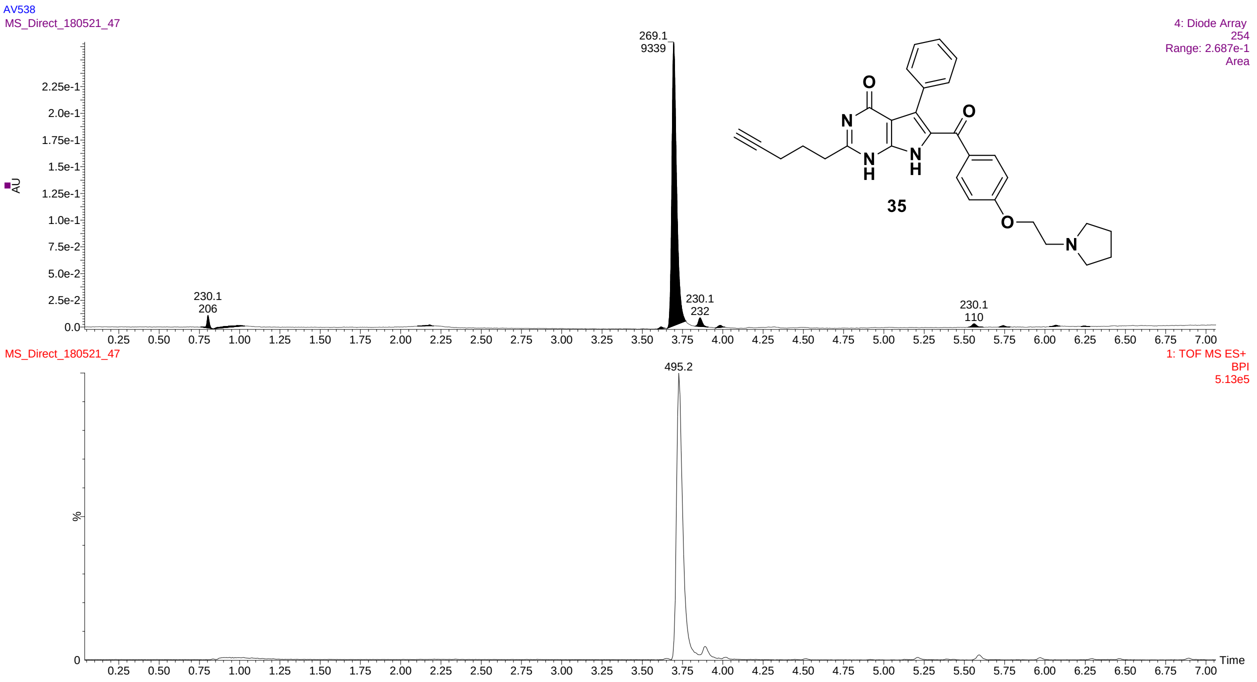

Purity: 97%

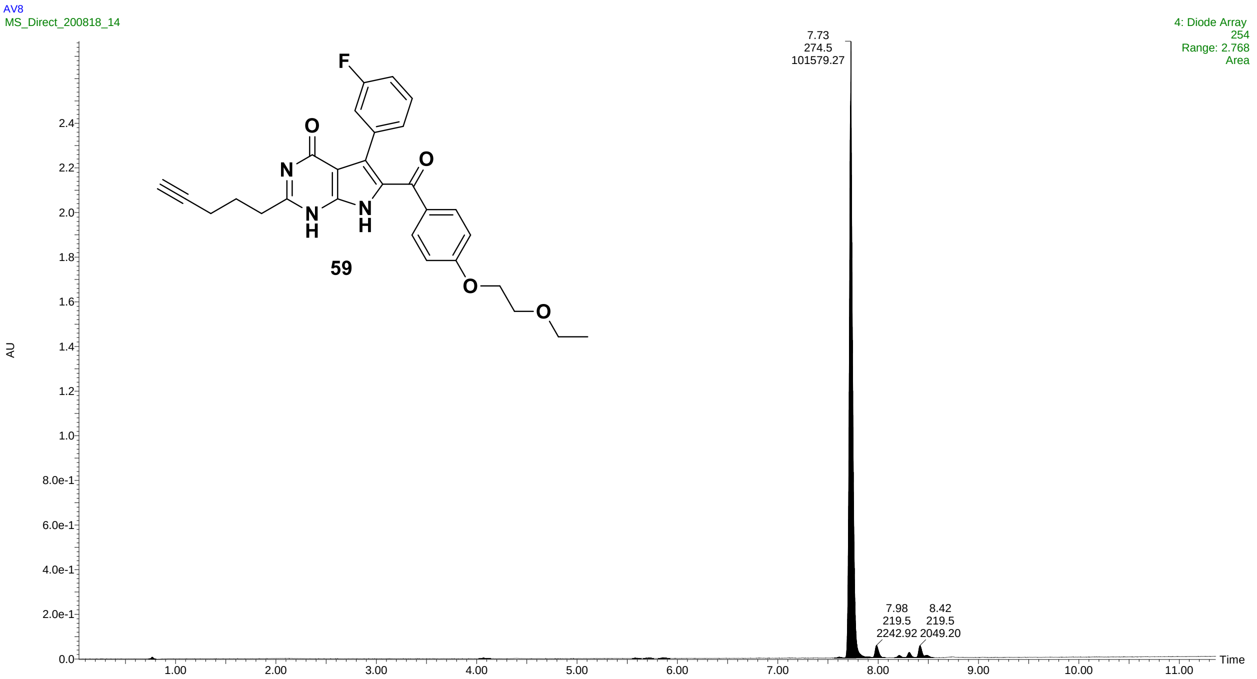

Purity: 93.7%

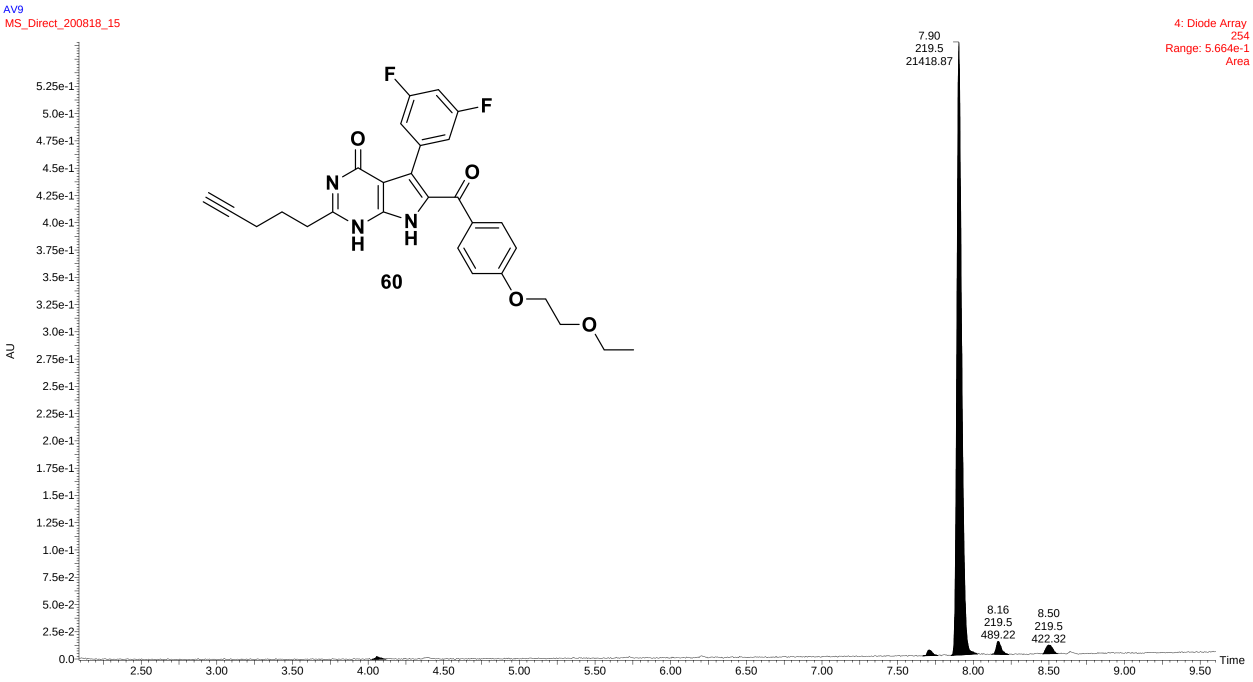

Purity: 94.6%

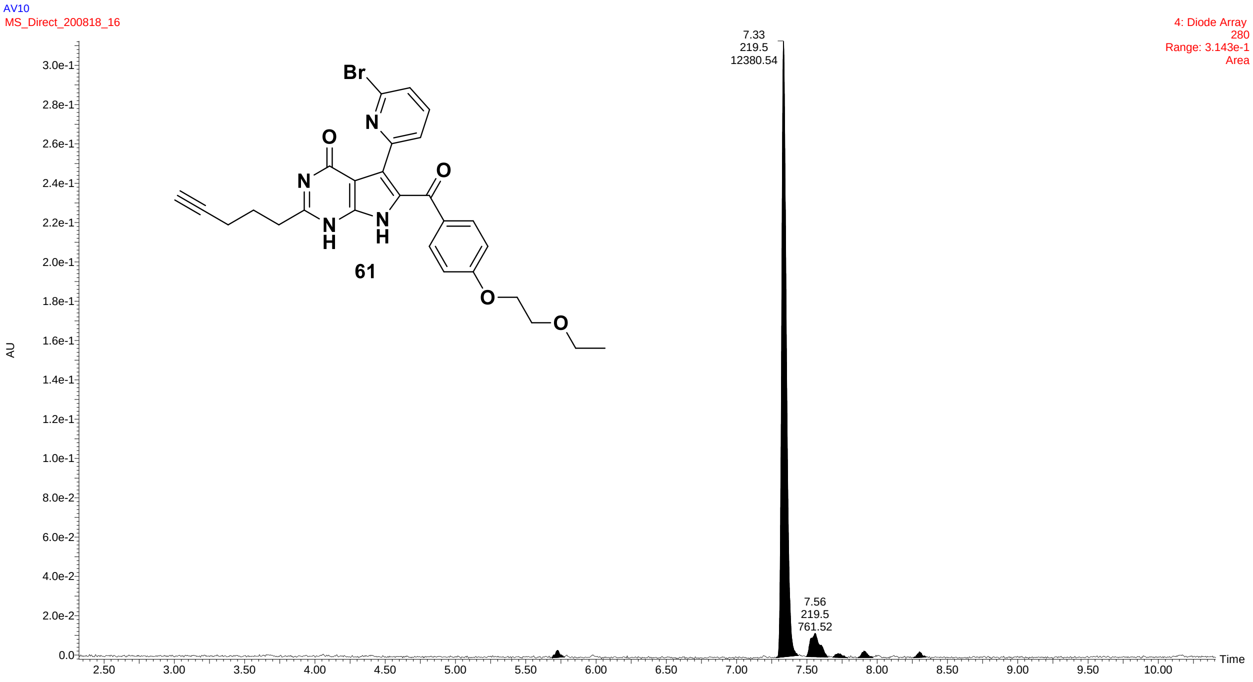

Purity: 90.6%

References:

1. Armarego, W. L. F.; Chai, C. L. L., *Purification of Laboratory Chemicals*. 7th ed.; Butterworth-Heinemann: 2013; Vol. .
2. *APEX3, SAINT, and SADABS; Bruker AXS Inc.: Madison, WI,*, 2018.
3. Sheldrick, G. M., A short history of SHELX. *Acta Crystallogr., Sect. A: Found. Crystallogr.* **2008**, *A64*, 112-122.
4. Sheldrick, G. M., SHELXT - Integrated space-group and crystal-structure determination. *Acta Crystallogr., Sect. A: Found. Crystallogr.* **2015**, *A71*, 3-8.
5. Sheldrick, G. M., Crystal structure refinement with SHELXL. *Acta Crystallogr., Sect. C: Struct. Chem.* **2015**, *C71*, 3-8.
6. Atwood, J. L.; Barbour, L. J., Molecular graphics: From science to art. *Cryst. Growth Des.* **2003**, *3*, 3-8.
7. Barbour, L. J., X-seed - A software tool for supramolecular crystallography. *J. Supramol. Chem.* **2001**, *1*, 189-191.
8. *POV-Ray for Windows*, 3.6; Persistence of Vision Raytracer Pty. Ltd.: Williamstown, 2004.
9. Ranaivoson, F. M.; Gigant, B.; Berritt, S.; Joullie, M.; Knossow, M., Structural plasticity of tubulin assembly probed by vinca-domain ligands. *Acta Crystallogr., Sect. D* **2012**, *68*, 927-934.
10. Miura, T.; Biyajima, T.; Fujii, T.; Murakami, M., Synthesis of α-Amino Ketones from Terminal Alkynes via Rhodium-Catalyzed Denitrogenative Hydration of N-Sulfonyl-1,2,3-triazoles. *J. Am. Chem. Soc.* **2012**, *134*, 194-196.
11. Duclos, S.; Stoeckli-Evans, H.; Ward, T. R., Design and Synthesis of Compartmental Ligands and their Complexes for the Production of Catalytic Antibodies. *Helv. Chim. Acta* **2001**, *84*, 3148-3161.
12. Scott, R.; Karki, M.; Reisenauer, M. R.; Rodrigues, R.; Dasari, R.; Smith, W. R.; Pelly, S. C.; van Otterlo, W. A. L.; Shuster, C. B.; Rogelj, S.; Magedov, I. V.; Frolova, L. V.; Kornienko, A., Synthetic and Biological Studies of Tubulin Targeting C2-Substituted 7-Deazahypoxanthines Derived from Marine Alkaloid Rigidins. *ChemMedChem* **2014**, *9*, 1428-1435.
13. Moriyama, K.; Hamada, T.; Nakamura, Y.; Togo, H., Catalytic dehydrogenative dual functionalization of ethers: dealkylation–oxidation–bromination accompanied by C–O bond cleavage via aerobic oxidation of bromide. *Chem. Commun.* **2017**, *53*, 6565-6568.
14. Perez, D. I.; Palomo, V.; Pérez, C.; Gil, C.; Dans, P. D.; Luque, F. J.; Conde, S.; Martínez, A., Switching Reversibility to Irreversibility in Glycogen Synthase Kinase 3 Inhibitors: Clues for Specific Design of New Compounds. *J. Med. Chem.* **2011**, *54*, 4042-4056.
15. Noji, M.; Ohno, T.; Fuji, K.; Futaba, N.; Tajima, H.; Ishii, K., Secondary Benzylation Using Benzyl Alcohols Catalyzed by Lanthanoid, Scandium, and Hafnium Triflate. *J. Org. Chem.* **2003**, *68*, 9340-9347.
16. Cao, B.; Ding, H.; Yang, R.; Wang, X.; Xiao, Q., Total synthesis of a marine alkaloid—rigidin E. *Mar. Drugs* **2012**, *10*, 1412-1421.
